# Data extraction methods for systematic review (semi)automation: A living systematic review

**DOI:** 10.12688/f1000research.51117.1

**Published:** 2021-05-19

**Authors:** Lena Schmidt, Babatunde K. Olorisade, Luke A. McGuinness, James Thomas, Julian P. T. Higgins

**Affiliations:** 1 Bristol Medical School, University of Bristol, Bristol, BS8 2PS, UK; 2 Sciome LLC, Research Triangle Park, North Carolina, 27713, USA; 3 Cardiff School of Technologies, Cardiff Metropolitan University, Cardiff, CF5 2YB, UK; 4 UCL Social Research Institute, University College London, London, WC1H 0AL, UK

**Keywords:** Data Extraction, Natural Language Processing, Reproducibility, Systematic Reviews, Text Mining

## Abstract

**Background:** The reliable and usable (semi)automation of data extraction can support the field of systematic review by reducing the workload required to gather information about the conduct and results of the included studies. This living systematic review examines published approaches for data extraction from reports of clinical studies.

**Methods:** We systematically and continually search MEDLINE, Institute of Electrical and Electronics Engineers (IEEE), arXiv, and the *dblp computer science bibliography* databases. Full text screening and data extraction are conducted within an open-source living systematic review application created for the purpose of this review. This iteration of the living review includes publications up to a cut-off date of 22 April 2020.

**Results: **In total, 53 publications are included in this version of our review. Of these, 41 (77%) of the publications addressed extraction of data from abstracts, while 14 (26%) used full texts. A total of 48 (90%) publications developed and evaluated classifiers that used randomised controlled trials as the main target texts. Over 30 entities were extracted, with PICOs (population, intervention, comparator, outcome) being the most frequently extracted. A description of their datasets was provided by 49 publications (94%), but only seven (13%) made the data publicly available. Code was made available by 10 (19%) publications, and five (9%) implemented publicly available tools.

**Conclusions:** This living systematic review presents an overview of (semi)automated data-extraction literature of interest to different types of systematic review. We identified a broad evidence base of publications describing data extraction for interventional reviews and a small number of publications extracting epidemiological or diagnostic accuracy data. The lack of publicly available gold-standard data for evaluation, and lack of application thereof, makes it difficult to draw conclusions on which is the best-performing system for each data extraction target. With this living review we aim to review the literature continually.

## 1. Introduction

In a systematic review, data extraction is the process of capturing key characteristics of studies in structured and standardised form based on information in journal articles and reports. It is a necessary precursor to assessing the risk of bias in individual studies and synthesising their findings. Interventional, diagnostic, or prognostic systematic reviews routinely extract information from a specific set of fields that can be predefined. ^1^ The most common fields for extraction in interventional reviews are defined in the PICO framework (population, intervention, comparison, outcome) and similar frameworks are available for other review types. The data extraction task can be time-consuming and repetitive when done by hand. This creates opportunities for support through intelligent software, which identify and extract information automatically. When applied to the field of health research, this semi-automation sits at the interface between evidence-based medicine (EBM) and data science, and as described in the following section, interest in its development has grown in parallel with interest in AI in other areas of computer science.

### 1.1 Related systematic reviews and overviews

This review is, to the best of our knowledge, the only living systematic review of data extraction methods. We have identified four previous reviews of tools and methods, ^2– 5^ two documents providing overviews and guidelines relevant to our topic, ^6, 7^ and an ongoing effort to list published tools for different parts of the systematic reviewing process. ^8^

A recent systematic review of machine-learning for systematic review automation, published in Portuguese, included 35 publications. The authors examined journals in which publications about systematic review automation are published, and conducted a term-frequency and citation analysis. They categorised papers by systematic review task, and provided a brief overview of data extraction methods. ^2^

In 2014, Tsafnat *et al.* provided a broad overview on automation technologies for different stages of authoring a systematic review. ^5^ O’Mara-Eves *et al*. published a systematic review focusing on text-mining approaches in 2015. ^4^ It includes a summary of methods for the evaluation of systems, such as recall, accuracy, and F1 score (the harmonic mean of recall and precision, a metric frequently used in machine-learning). The reviewers focused on tasks related to PICO classification and supporting the screening process. In the same year, Jonnalagadda, Goyal and Huffman ^3^ described methods for data extraction, focusing on PICOs and related fields.

Beller *et al.* describe principles for development and integration of tools for systematic review automation. ^6^ Marshall and Wallace ^7^ present a guide to automation technology, with a focus on availability of tools and adoption into practice. They conclude that tools facilitating screening are widely accessible and usable, while data extraction tools are still at piloting stages or require a higher amount of human input.

The systematic reviews from 2014 to 2015 present an overview of classical machine learning and natural language processing (NLP) methods applied to tasks such as data mining in the field of evidence-based medicine. At the time of publication of these documents, methods such as topic modelling (Latent Dirichlet Allocation) and support vector machines (SVM) were considered state-of-the art for language models. The age of these publications means that the latest static or contextual embedding-based and neural methods are not included. These newer methods, ^9^ however, are used in contemporary systematic review automation software which will be reviewed in the scope of this living review.

### 1.2 Aim

We aim to review published methods and tools aimed at automating or semi-automating the process of data extraction in the context of a systematic review of medical research studies. We will do this in the form of a living systematic review, keeping information up to date and relevant to the challenges faced by systematic reviewers at any time.

Our objectives in reviewing this literature are two-fold. First, we want to examine the methods and tools from the data science perspective, seeking to reduce duplicate efforts, summarise current knowledge, and encourage comparability of published methods. Second, we seek to highlight the added value of the methods and tools from the perspective of systematic reviewers who wish to use (semi) automation for data extraction, i.e., what is the extent of automation? Is it reliable? We address these issues by summarising important caveats discussed in the literature, as well as factors that facilitate the adoption of tools in practice.

## 2. Methods

### 2.1 Registration/protocol

This review was conducted following a preregistered and published protocol. ^10, 11^ PROSPERO was initially considered as platform for registration, but it is limited to reviews with health-related outcomes. Any deviations from the protocol have been described below.

### 2.2 Living review methodology

We are conducting a living review because the field of systematic review (semi) automation is evolving rapidly along with advances in language processing, machine-learning and deep-learning.

**Figure 1.**
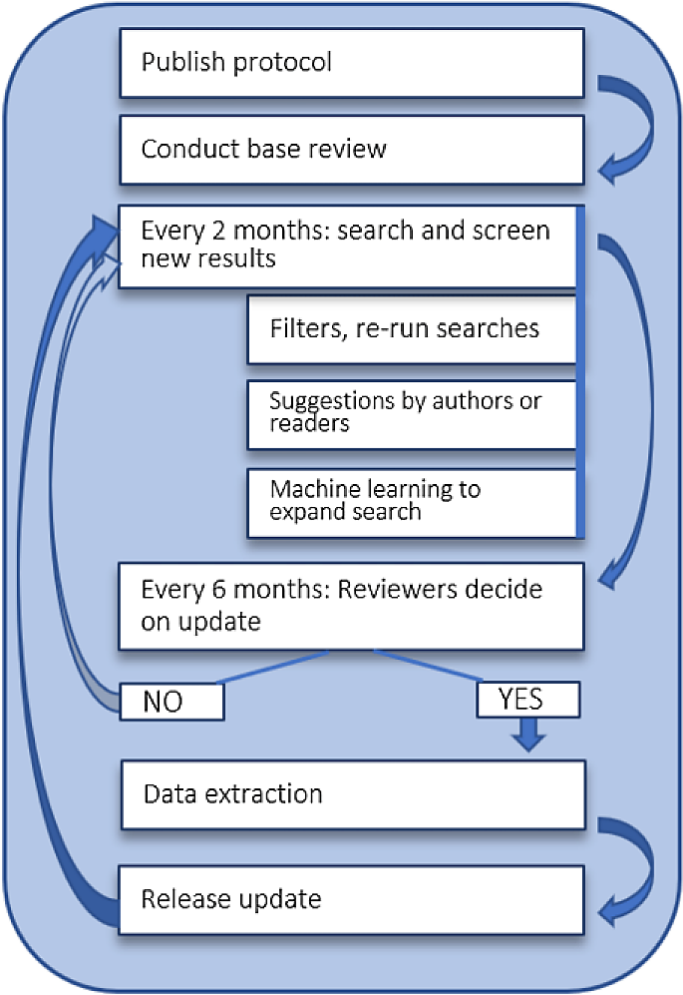
Continuous updating of the living review. This image is reproduced under the terms of a Creative Commons Attribution 4.0 International license (CC-BY 4.0) from Schmidt et al, 2020. ^11^

The process of updating started as described in the protocol ^11^ and is shown in Figure 1. In short, we will continuously update the literature search results, using the search strategies and methods described in the section ‘Search’ below. MEDLINE and arXiv search results are updated daily in a completely automated fashion. Articles from the dblp and IEEE are added every two months. All search results are automatically imported to our living review screening and data extraction web-application, which is described in the section ‘Data collection and analysis’ below.

The decision for full review updates is made every six months based on the number of new publications added to the review. For more details about this, please refer to the protocol or to the Cochrane living systematic review guidance. In between updates, the screening process and current state of the data extraction is visible via the living review website.

### 2.3 Eligibility criteria

• We included full text publications that describe an original NLP approach for extracting data related to systematic reviewing tasks. Data fields of interest (referred to here as entities or as sentences) were adapted from the Cochrane Handbook for Systematic Reviews of Interventions, ^1^ and are defined in the protocol. ^11^ We included the full range of NLP methods (e.g., regular expressions, rule-based systems, machine learning, and deep neural networks).
• Publications must describe a full cycle of the implementation and evaluation of a method. For example, they must report training and at least one measure of evaluating the performance of a data extraction algorithm.
• We included reports published from 2005 until the present day, similar to. ^3^ We would have translated non-English reports, had we found any.
• The data that the included publications use for mining must be texts from randomised controlled trials, comparative cohort studies, case control studies or comparative cross-sectional studies (e.g., for diagnostic test accuracy). The scope of data extraction methods can be applied to the full text or to abstracts within each eligible publication’s corpus. We included publications that extracted data from other study types, as long as at least one of our study types of interest was contained in the corpus.

We excluded publications reporting:

• Methods and tools related solely to image processing and importing biomedical data from PDF files without any NLP approach, including data extraction from graphs.
• Any research that focuses exclusively on protocol preparation, synthesis of already extracted data, write-up, pre-processing of text and dissemination.
• Methods or tools that provided no natural language processing approach and offered only organisational interfaces, document management, databases, or version control
• Any publications related to electronic health reports or mining genetic data.

### 2.4 Search

We searched five electronic databases, using the search methods previously described in our protocol. ^11^ In short, we searched MEDLINE via Ovid, using a search strategy developed with the help of an information specialist, and searched Web of Science Core Collection and IEEE using adaptations of this strategy, which were made by the review authors. Searches on the arXiv (computer science) and dblp were conducted on full database dumps using the search functionality described by McGuinness and Schmidt. ^12^ The full search results and further information about document retrieval are available in *Underlying data:* Appendix A and B. ^86^

Originally, we planned to include a full literature search from the Web of Science Core Collection. Due to the large number of publications retrieved via this search (n = 7822) we decided to first screen publications from all other sources, to train a machine-learning ensemble classifier, and to only add publications that were predicted as relevant for our living review. This reduced the Web of Science Core Collection publications to 547 abstracts, which were added to the studies in the initial screening step. The dataset, code and weights of trained models are available in *Underlying data:* Appendix C. ^86^ This includes plots of each model’s evaluation in terms of area under the curve (AUC), accuracy, F1, recall, and variance of cross-validation results for every metric.

In future iterations of this living review we plan to change to PubMed for searching MEDLINE. This decision was made to facilitate continuous reference retrieval.

### 2.5 Data collection and analysis

***2.5.1 Selection of studies***

Screening and data extraction were conducted as stated in the protocol. In short, we initially screened all retrieved publications using the Abstrackr tool. All abstracts were screened by two independent reviewers. Conflicting judgements were resolved by the authors who made the initial screening decisions. Full texts screening was conducted in a similar manner to abstract screening but used our web application for living systematic reviews described in the following section.

***2.5.2 Data extraction, assessment, and management***

We previously developed a web application to automate reference retrieval for living review updates (see *Software availability* ^13^), to support both abstract and full text screening for review updates, and to manage the data extraction process throughout. ^13^ For future updates of this living review we will use the web application, and not Abstrackr, for screening references. This web application is already in use by another living review. ^14^ It automates daily reference retrieval from the included sources and has a screening and data extraction interface. All extracted data are stored in a database. Figures and tables can be exported on a daily basis and the progress in between review updates is shared on our living review website. The full spreadsheet of items extracted from each included reference is available in the *Underlying data*. ^86^ As previously described in the protocol, quality of reporting and reproducibility was assessed based on a previously published checklist for reproducibility in text mining. ^15^

As planned in the protocol, a single reviewer conducted data extraction, and a random 10% of the included publications were checked by a second reviewer.

***2.5.3 Visualisation***

The creation of all figures and interactive plots on the living review website and in this review’s ‘Results’ section was automated based on structured content from our living review database (see Appendix A, *Underlying data* ^86^). We automated the export of PDF reports for each included publication. Calculation of percentages, export of extracted text, and creation of figures was also automated.

***2.5.4 Accessibility of data***

All data and code are free to access. A detailed list of sources is given in the ‘Data availability’ and ‘Software availability’ sections.

### 2.6 Changes from protocol

In the protocol we stated that data would be available via an OSF repository. Instead, the full review data are available via the Harvard Dataverse, as this repository allows us to keep an assigned DOI after updating the repository with new content for each iteration of this living review.

We also stated that we would screen all publications from the Web of Science search. Instead, we describe a changed approach in the Methods section, under ‘Search’.

We added a data extraction item for the type of information which a publication mines (e. g. P, IC, O) into the section of primary items of interest, and we moved the type of input and output format from primary to secondary items of interest. We grouped the secondary item of interest ‘Other reported metrics, such as impacts on systematic review processes (e.g., time saved during data extraction)’ with the primary item of interest ‘Reported performance metrics used for evaluation’.

The item ‘Persistence: is the dataset likely to be available for future use?’ was changed to: ‘Can data be retrieved based on the information given in the publication?’. We decided not to speculate if a dataset is likely to be available in the future and chose instead to record if the dataset was available at the time when we tried to access it.

The item ‘Can we obtain a runnable version of the software based on the information in the publication?’ was changed to ‘Is an app available that does the data mining, e.g. a web-app or desktop version?’.

In this current version of the review we did not yet contact the authors of the included publications. This decision was made due to time constraints, however reaching out to authors is planned as part of the first update to this living review.

## 3. Results

### 3.1 Results of the search

**Figure 2.**
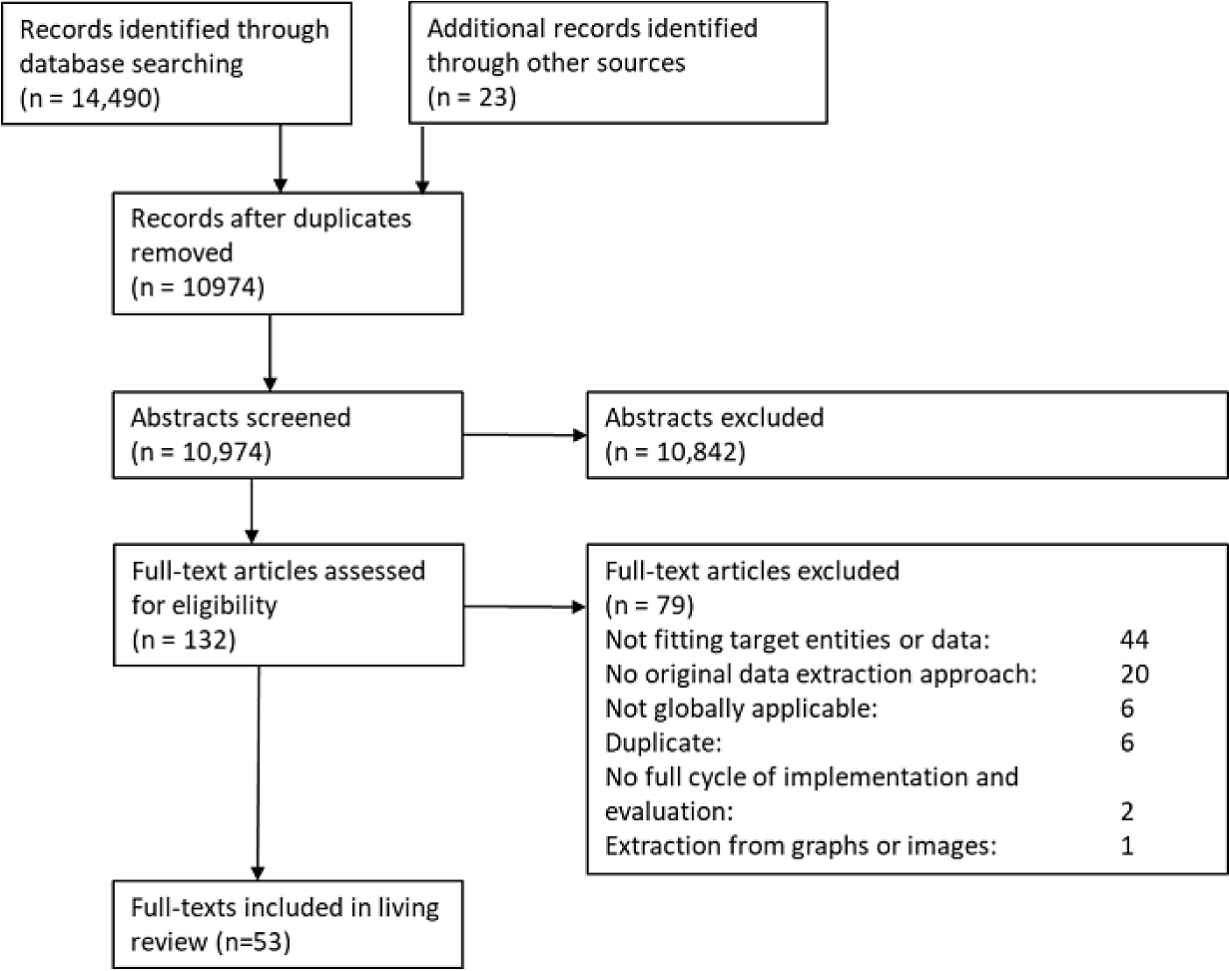
PRISMA flow diagram.

Our database searches identified 10,974 publications after duplicates were removed (see Figure 2). We identified an additional 23 publications by screening the bibliographies of included publications, in addition to reviewing the tools contained in the SRToolbox. These records were missed from our search because they were either publications from the Association for Computational Linguistics Fr (ACL, n = 7) or were otherwise not indexed or found via Ovid/MEDLINE: (n = 5). For future review updates we will adapt the search strategies and conduct searches in sources such as the ACL.

This iteration of the living review includes 53 publications, summarised in Table 1 in *Underlying data* ^86^). Twelve of these were among the additional 23 publications.

***3.1.1 Excluded publications***

In total, 79 publications were excluded at the full text screening stage, with the most common reason for exclusion being that a study did not fit target entities or target data. In most cases, this was due to the text-types mined in the publications. Electronic health records and non-trial data were common, and we created a list of datasets that would be excluded in this category (see more information in *Underlying data:* Appendix B ^86^). Some publications addressed the right kind of text but were excluded for not mining entities of interest to this review. For example, Norman, Leeflang and Névéol ^16^ performed data extraction for diagnostic test accuracy reviews, but focused on extracting the results and data for statistical analyses. Millard, Flach and Higgins ^17^ and Marshall, Kuiper and Wallace ^18^ looked at risk of bias classification, which is beyond the scope of this review. Boudin, Nie and Dawes ^19^ developed a weighing scheme based on an analysis of PICO element locations, leaving the detection of single PICO elements for future work. Luo *et al*. ^20^ extracted data from clinical trial registrations but focused on parsing inclusion criteria into event or temporal entities to aid participant selection for randomised controlled trials (RCTs).

The second most common reason for study exclusion was that they had ‘no original data extraction approach’. Rathbone *et al*., ^21^ for example, used hand-crafted Boolean searches specific to a systematic review’s PICO criteria to support the screening process of a review within Endnote. We classified this article as not having any original data extraction approach because it does not create any structured outputs specific to P, IC, or O. Malheiros *et al.* ^22^ performed visual text mining, supporting systematic review authors by document clustering and text highlighting. Similarly, Fabbri *et al.* ^23^ implemented a tool that supports the whole systematic review workflow, from protocol to data extraction, performing clustering and identification of similar publications. Other systematic reviewing tasks that can benefit from automation but were excluded from this review are listed in *Underlying data:* Appendix B. ^86^

### 3.2 Results from the data extraction: Primary items of interest

***3.2.1 Automation approaches used***

**Figure 3.**
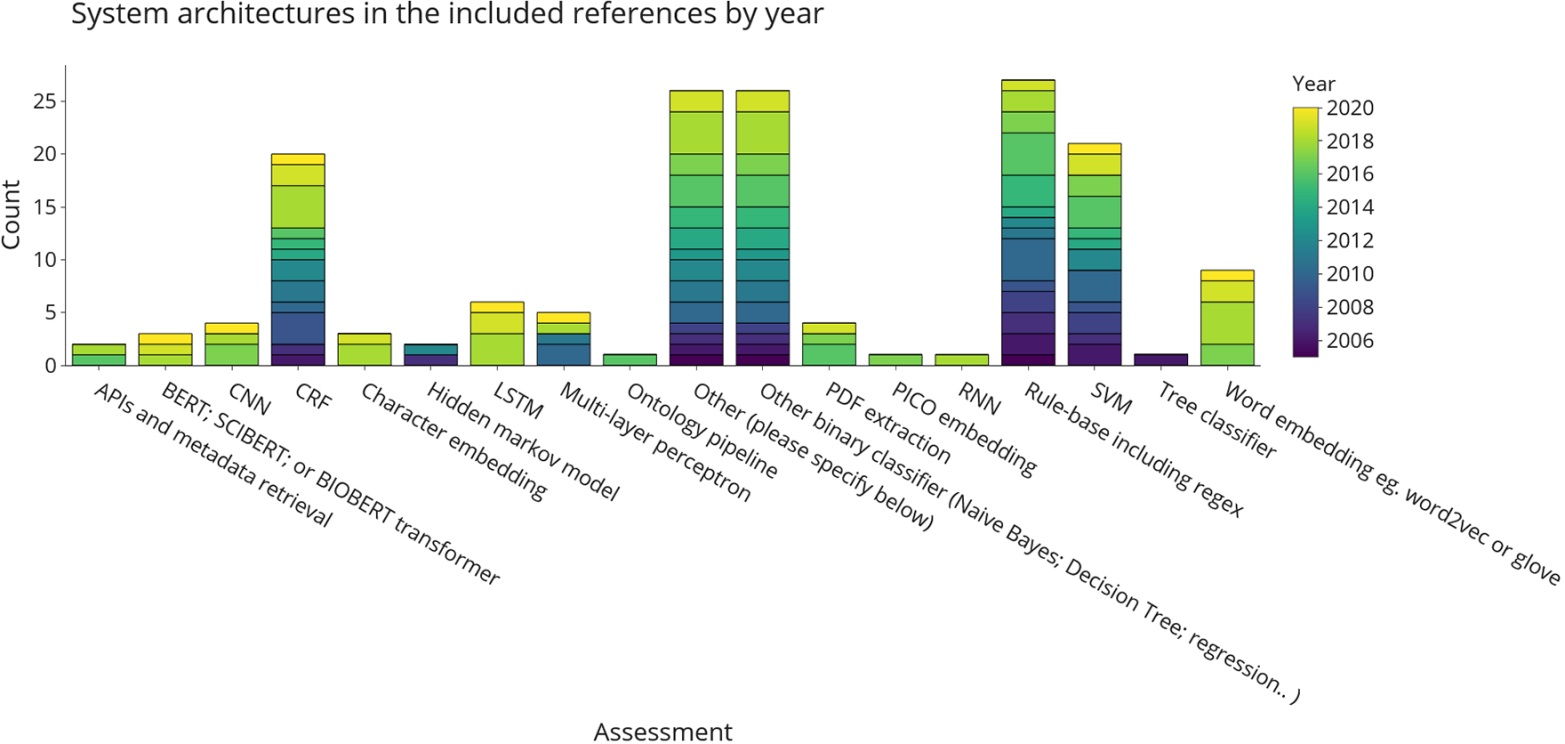
System architectures used for automating data extraction in the included publications. Results are divided into different categories of machine-learning and natural language processing approaches and coloured by the year of publication. More than one architecture component per publication is possible. Where API, application programming interface; BERT, bidirectional encoder representations decision trees; CNN, convolutional neural network; CRF, conditional random fields; LSTM, long short-term memory; PCIO, population, intervention, comparison, outcome; RNN, recurrent neural networks; SVM, support vector machines.

Figure 3 shows aspects of the system architectures implemented in the included publications. A short summary of these for each publication is provided in Table 1 in *Underlying data*. ^86^ Where possible, we tried to break down larger system architectures into smaller components. For example, an architecture combining a word embedding + long short-term memory (LSTM) network would have been broken down into the two respective sub-components. We grouped binary classifiers, such as naïve Bayes and Bidirectional Encoder Representations decision trees (BERT). Although SVM is also binary classifier, it was assigned as separate category due to its popularity. The final categories are a mixture of non-machine-leaning automation (application programming interface (API) and metadata retrieval, PDF extraction, rule-base), classic machine-learning (naïve Bayes, decision trees, SVM, or other binary classifiers) and neural or deep-learning approaches (convolutional neural network (CNN), LSTM, transformers, or word embeddings). This figure shows that there is no obvious choice of system architecture for this task.

Binary classifiers, most notably naïve Bayes and SVMs, are the most frequently used system components in the data extraction literature. They are frequently used in studies published between 2005 and now.

Rule-bases, including approaches using heuristics, wordlists, and regular expressions, were one of the earliest techniques used for data extraction in EBM literature. It remains one of the most frequently used approaches to automation. Eight publications (15%) use rule-bases alone, while the rest of the publications use them in combination with other classifiers (data shown in *Underlying data:* Appendix A ^86^). Although used more frequently in the past, the five publications published between 2017 and now that use this approach combine it with conditional random fields (CRF), ^24^ use it alone, ^25, 26^ use it with SVM ^27^ or use it with other binary classifiers. ^28^ In practice, these systems use rule-bases in the form of hand-crafted lists to identify candidate phrases for amount entities such as sample size ^25, 28^ or to refine a result obtained by a machine-learning classifier on the entity level (e.g., instances where a specific intervention or outcome is extracted from a sentence). ^24^ Embedding and neural architectures are increasingly being used in literature from the past five years. Recurrent neural networks (RNN), CNN, and LSTM networks require larger amounts of training data, but by using embeddings or pre-training algorithms based on unlabelled data they have become increasingly more interesting in fields such as data extraction for EBM, where high-quality training data are difficult and expensive to obtain.

In the ‘Other’ category, tools mentioned were mostly other classifiers such as maximum entropy classifiers (n = 3), kLog, J48, and various position or document-length classification algorithms. We also added methods such as supervised distant supervision (n = 3, see ^29^) and novel training approaches to existing neural architectures in this category.

***3.2.2 Reported performance metrics used for evaluation***

**Figure 4.**
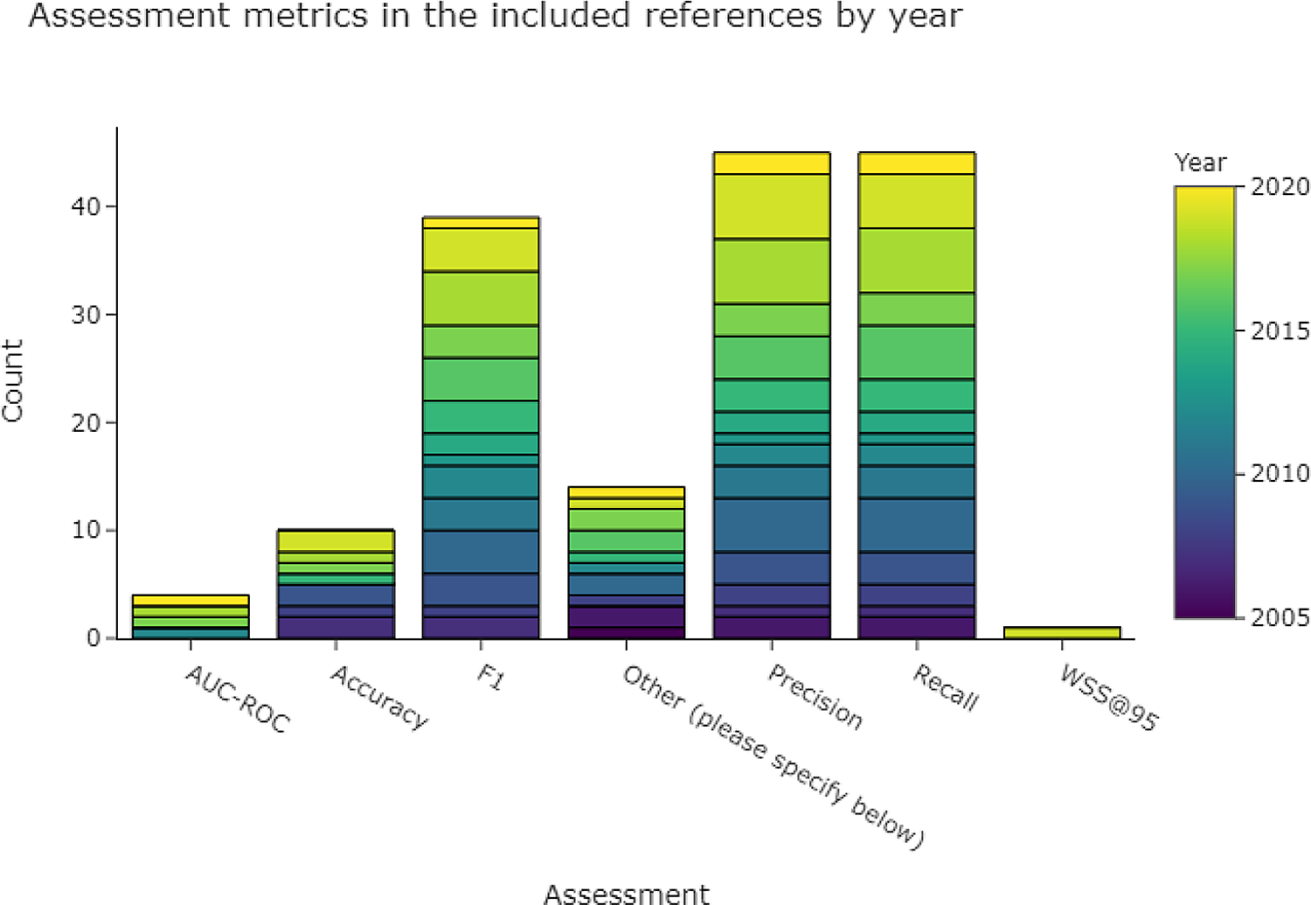
The most common assessment metrics used in the included publications in order to evaluate the performance of a data extraction system. More than one metric per publication is possible, which means that the total number of included publications (n = 53) is lower than the sum of counts of the bars within this figure. AUC-ROC, area under the curve - receiver operator characteristics; F1, harmonic mean of precision and recall.

Precision (i.e., positive predictive value), recall (i.e., sensitivity), and F1 score (harmonic mean of precision and recall) are the most widely used metrics for evaluating classifiers. This is reflected in Figure 4, which shows that at least one of these metrics was used in almost all of the 53 included publications. Accuracy and area under the curve - receiver operator characteristics (AUC-ROC) were less frequently used.

In the category ‘Other’ we added several instances where a relaxation of a metric was introduced, e. g., precision using top-n classified sentences ^29– 31^ or mean average precision and the metric ‘precision @rank 10’ for sentence ranking exercises. ^32, 33^ Another type of relaxation for standard metrics is a distance relaxation when normalising entities into concepts in medical subject headings (MesH) or unified medical language system (UMLS), to allow N hops between predicted and target concepts. ^34^ Other metrics were kappa, ^33^ random shuffling ^35^ or binomial proportion test ^36^ to test statistical significance, given with confidence intervals. ^27^ Further metrics included under ‘Other’ were odds ratios, ^37^ normalised discounted cumulative gain, ^29^ ‘sentences needed to screen per article’ in order to find one relevant sentence, ^38^ McNemar test, ^36^ C-statistic (with 95% CI) and Brier score (with 95% CI). ^39^

Real-life evaluations, such as the percentage of outputs needing human correction, or time saved per article, were reported by one publication, ^30^ and an evaluation as part of a wider screening system was done in another. ^40^

There were several approaches and justifications of using macro- or micro-averaged precision, recall, or F1 scores in the included publications. Micro or macro scores are computed in multi-class cases, and the final scores can have a difference if the classes in a dataset are imbalanced (as is the case in most datasets used in the included studies for this review).

Micro and macro scores were reported by, ^30, 41^ whereas ^26, 42^ reported micro across documents, and macro across the classes. Micro scores were used by ^41^ for class-level results.

Micro scores were also used by, ^44– 46^ and were used in the evaluation script of. ^47^

***3.2.3 Type of data***

3.2.3.1 Scope

Most data extraction is carried out on abstracts (See Table 1 in *Underlying data*, ^86^ and Table 5). Abstracts are the most practical choice, due to the possibility of exporting them along with literature search results from databases such as MEDLINE. Descriptions of the benefits of using full texts for data extraction include having access to a more complete dataset, while the benefits of using titles include lower complexity for the data extraction task. ^25^

**Table 5.** Examples for reports of inter-annotator disagreements in the included publications. Please see each included publication for further details on corpus quality.

Publication	Type	Score, or range between worst to best class
^25^	Average accuracy between annotators	Range: 0.62 to 0.70
^42^	Agreement rate	80%
^36^	Cohen’s Kappa	0.84 overall, down to 0.59 for worst class
^43^	Cohen’s Kappa	Range: 0.41 to 0.71
^48^	Inter-annotation recall	Range: 0.38 to 0.86
^47^	Cohen’s Kappa between experts	Range: 0.5 to 0.59
^47^	Macro-averaged worker vs. aggregation precision, recall, F1 (see publication for full scores)	Range: 0.39 to 0.70
^82^ (describes only PECODR corpus creation, excluded from review)	Initial agreement between annotators	Range: 85-87%
^44^	Average and range of agreement	62%, Range: 41-71
^33^	Avg. sentences labelled by expert vs. student per abstract	1.9 vs. 4.2
^33^	Cohen’s Kappa expert vs. student	0.42

Figure 6 shows that RCTs are the most common study design texts used for data extraction in the included publications (see also extended Table 1 in *Underlying data* ^86^). This is not surprising, because systematic reviews of interventions are the most common type of systematic review, and they are usually focusing on evidence from RCTs. Therefore, the literature for automation of data extraction focuses on RCTs, and their related PICO elements. Systematic reviews of diagnostic test accuracy are less frequent, and only one included publication specifically focused on text and entities related to these studies, ^48^ while another mentioned diagnostic procedures among other fields of interest. ^49^ Five publications focused on extracting data specifically from epidemiology research, or included text from cohort studies as well as RCT text. ^26, 42, 49– 51^ More publications mining data from surveys or case series might have been found if our search and review had concentrated on these types of texts.

**Figure 5.**
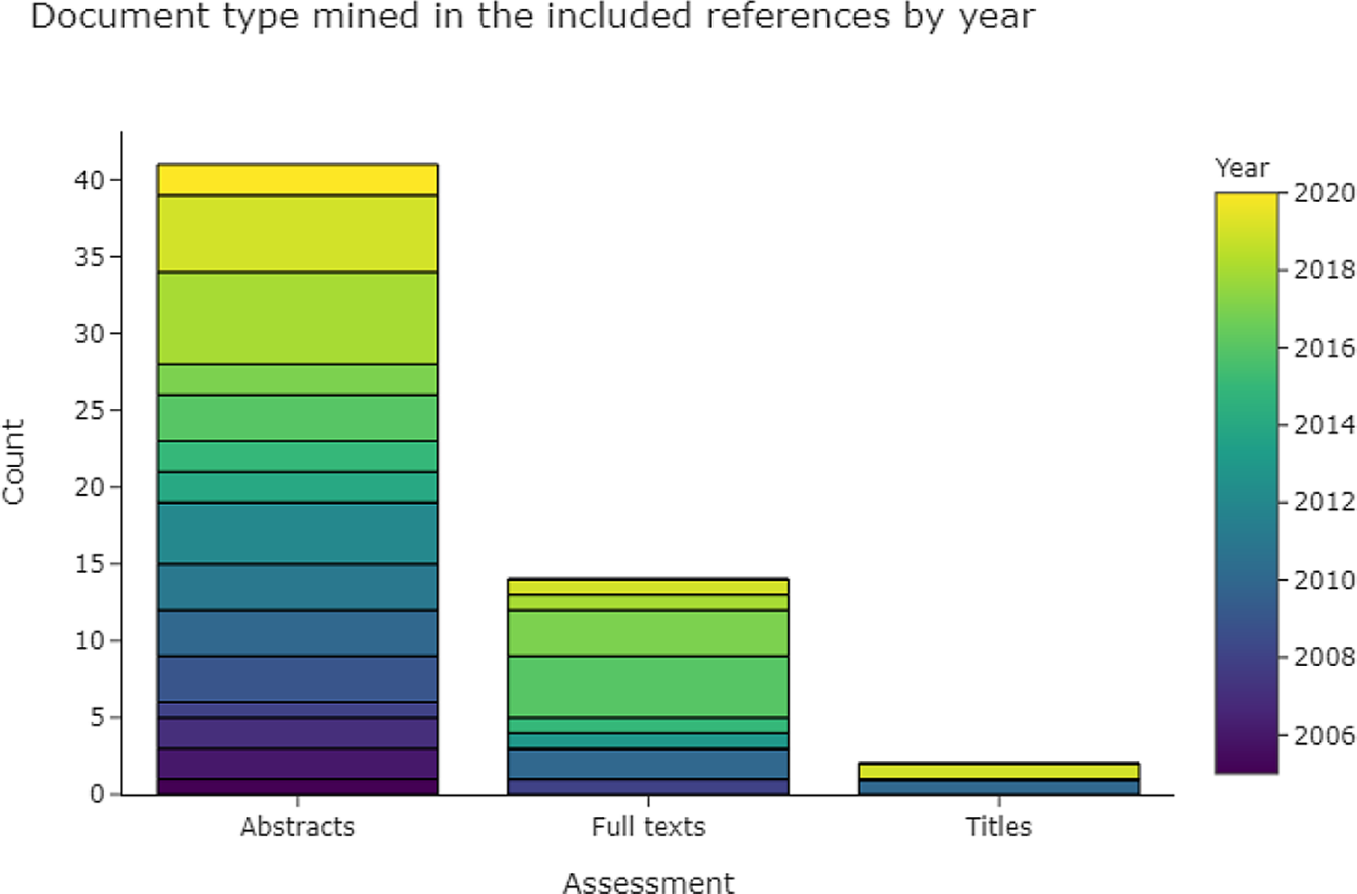
The most common assessment document type to conduct data extraction on, as used in the included publications. More than one type per publication is possible, which means that the total number of included publications (n = 53) is lower than the sum of counts of the bars within this figure.

**Figure 6.**
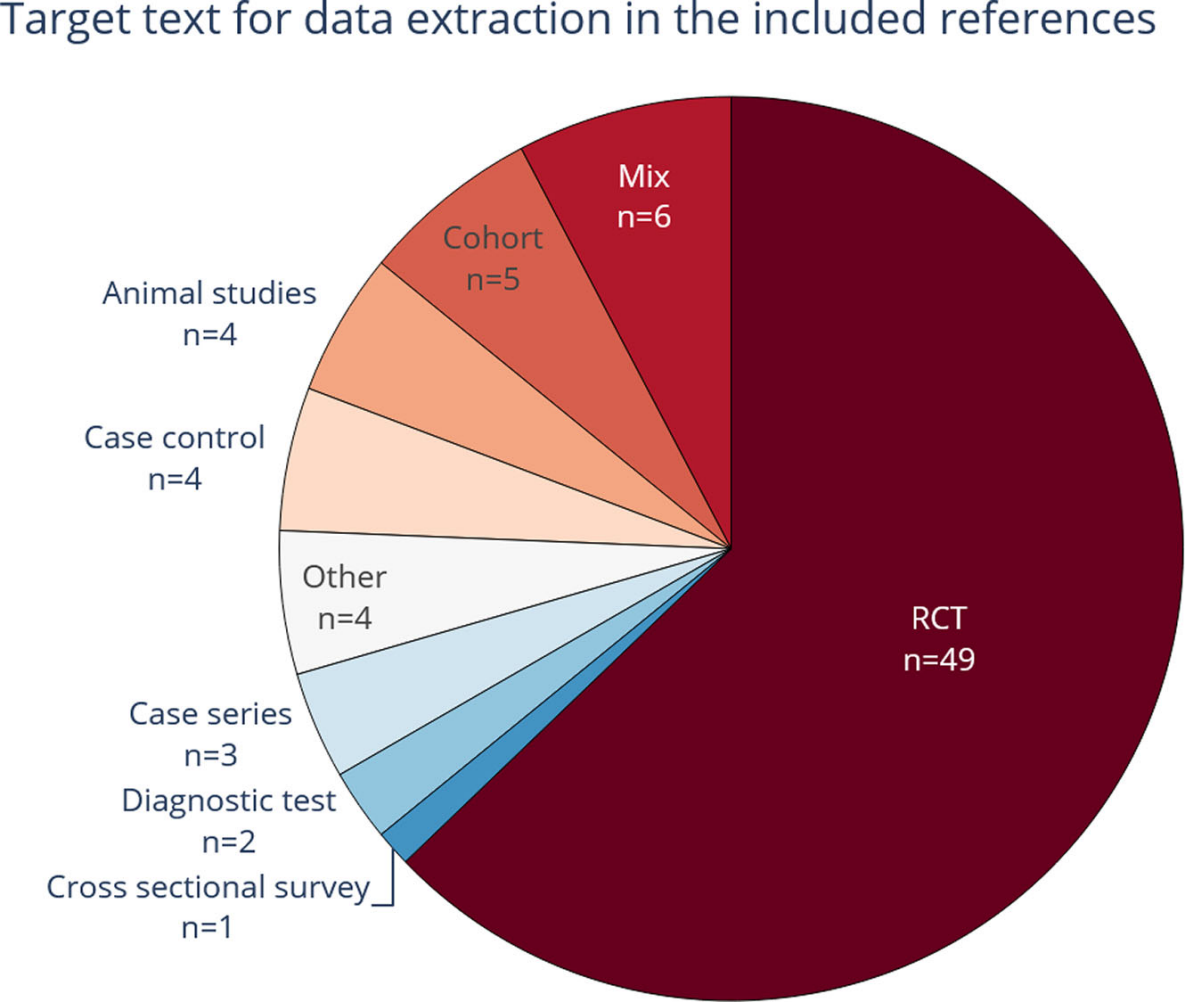
The study types from which data were extracted. Commonly, randomized controlled trials (RCT) text was at least one of the target text types used in the included publications.

3.2.3.2 Data extraction targets

**Figure 7.**
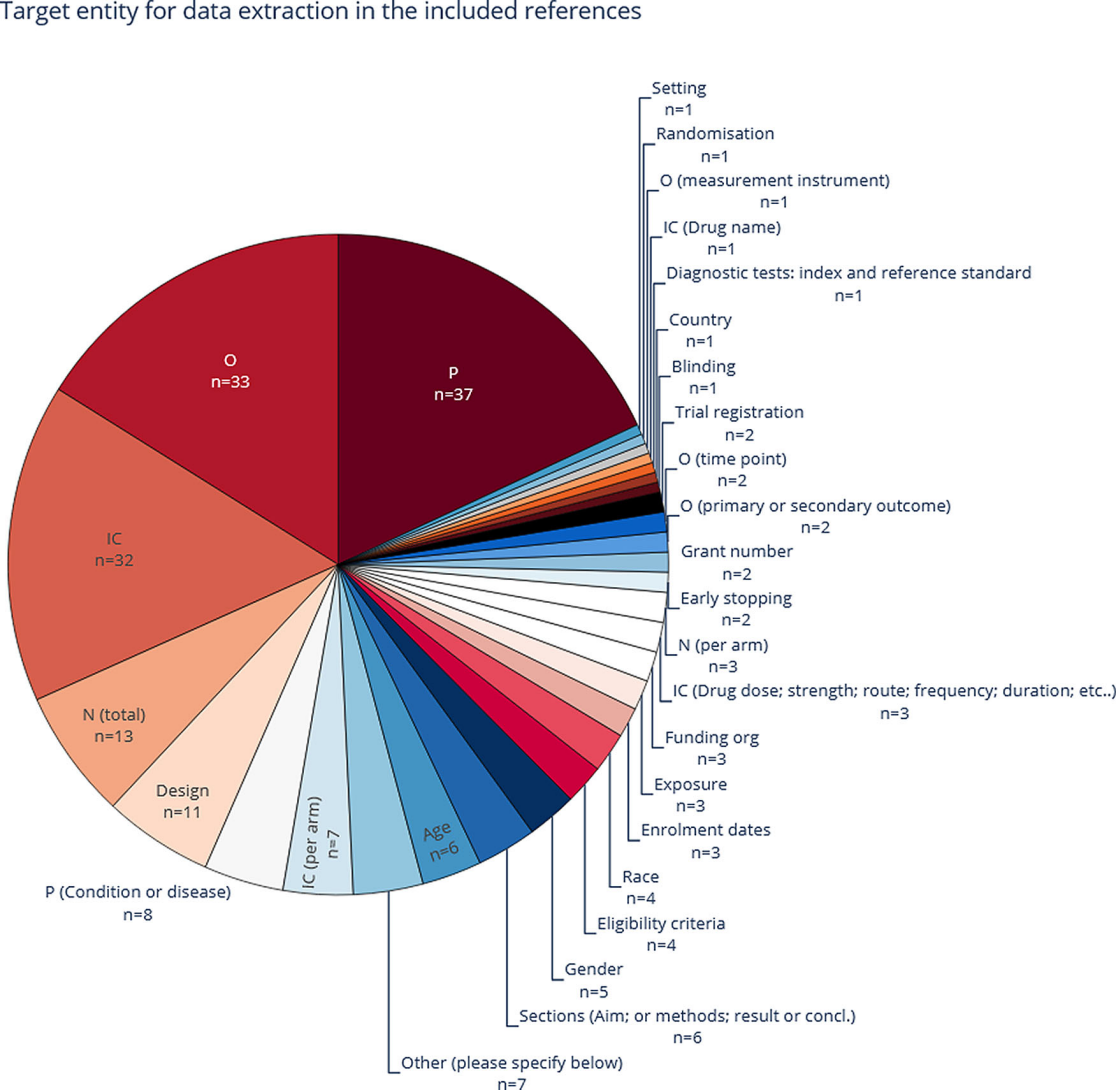
The most common entities, as extracted in the included publications. More than one entity type per publication is common, which means that the total number of included publications (n = 53) is lower than the sum of counts within this figure. P, population; I, intervention; C, comparison; O, outcome.

Mining P, IC, and O elements is the most common task performed in the literature of systematic review (semi-) automation (see Table 1 in *Underlying data*, ^86^ and Figure 7). However, some of the less-frequent data extraction targets in the literature can be categorised as sub-classes of a PICO., ^47^ for example, by annotating hierarchically multiple entity types such as health condition, age, and gender under the P class. The entity type ‘P (Condition and disease)’, was the most common entity closely related to the P class, appearing in seven included publications. ^40, 47, 49, 52– 55^

Notably, seven publications annotated or worked with datasets that differentiated between intervention and control arms. ^25, 30, 31, 37, 41, 56, 57^ Usually, I and C are merged (n=32). Most data extraction approaches focused on recognising instances of entity or sentence classes, and a small number of publications went one step further to normalise to actual concepts. ^34, 58^

The ‘Other’ category includes some more detailed drug annotations ^36^ or information such as confounders ^26^ and other entity types (see the full dataset in *Underlying data:* Appendix A for more information ^86^).

### 3.3 Results from the data extraction: Secondary items of interest

***3.3.1 Granularity of data extraction***

A total of 36 publications extracted at least one type of information at the entity level, while 32 publications used sentence level (see Table 1 extended version in *Underlying data* ^86^). We defined the entity level as any number of words that is shorter than a whole sentence, e.g., noun-phrases or other chunked text. Data types such as P, IC, or O commonly appeared to be extracted on both entity and sentence level, whereas ‘N’, the number of people participating in a study, was commonly extracted on entity level only.

***3.3.2 Type of input***

The majority of systems mentioned MEDLINE, or PubMed as the data source for text. Text files (n = 45), next to XML (n = 4), or HTML (n = 3), are the most common format of the data downloaded from these sources. Therefore, most systems described using, or were assumed to use, text files as input data. Eight included publications described using PDF files as input. ^29, 30, 34, 38, 48, 55, 59, 60^

***3.3.3 Type of output***

A limited number of publications described structured summaries as output of their extracted data (n = 9). Alternatives to exporting structured summaries were JSON (n = 3), XML, and HTML (n = 2 each). Most publications mentioned only classification scores without specifying an output type. In these cases, we assumed that the output would be saved as text files (n = 43).

### 3.4 Assessment of the quality of reporting

We used a list of 17 items to investigate reproducibility, transparency, description of testing, data availability, and internal and external validity of the approaches in each publication. The maximum and minimum number of items that were positively rated were 16 and 1, respectively, with a median of 10 (see Table 1 in *Underlying data* ^86^). Scores were added up and calculated based on the data provided in Appendix A (see *Underlying data* ^86^), using the sum and median functions integrated in Excel. Publications from recent years showed a trend towards more complete and clear reporting.

***3.4.1 Reproducibility***

3.4.1.1 Are the sources for training/testing data reported?

Of the included publications, 50 out of 53 (94%) clearly stated the sources of their data used for training and evaluation. MEDLINE was the most popular source of data, with abstracts usually described as being retrieved via searches on PubMed, or full texts from PubMed Central. A small number of publications described using text from specific journals such as PLoS Clinical Trials, New England Journal of Medicine, The Lancet, or BMJ. ^31, 56^ Texts and metadata from Cochrane, either provided in full or retrieved via PubMed, were used in five publications. ^32, 34, 38, 48, 59^ Corpora such as the ebm-nlp dataset, ^47^ or PubMed-PICO ^46^ are available for direct download. Publications published in recent years are increasingly reporting that they are using these benchmark datasets rather than creating and annotating their own corpora (see 4 for more details).

3.4.1.2 If pre-processing techniques were applied to the data, are they described?

Of the included publications, 47 out of 53 (89%) reported processing the textual data before applying/training algorithms for data extraction. Different types of pre-processing, with representative examples for usage and implementation, are listed in Table 1 below.

**Table 1.** Pre-processing techniques, a short description and examples from the literature.

Technique	Details	Example in literature
Tokenisation	Splitting text on sentence and word level	^31, 56, 61^
Normalisation	Replacing integers, units, dates, lower-casing	^36, 62, 63^
Lemmatisation and stemming	Reducing words to shorter or more common forms	^45, 64, 65^
Stop-word removal	Removing common words, such as ‘the’, from the text	^29, 42, 54^
Part-of-speech tagging and dependency parsing	Tagging words with their respective grammatical roles	^27, 52, 61^
Chunking	Defining sentence parts, such as noun-phrases	^36, 49, 66^
Concept tagging	Processing and tagging words with semantic classes or concepts, e. g. using word lists or MetaMap	^49, 53, 67^

***3.4.2 Transparency of methods***

3.4.2.1 Is there a description of the algorithms used?

**Figure 8.**
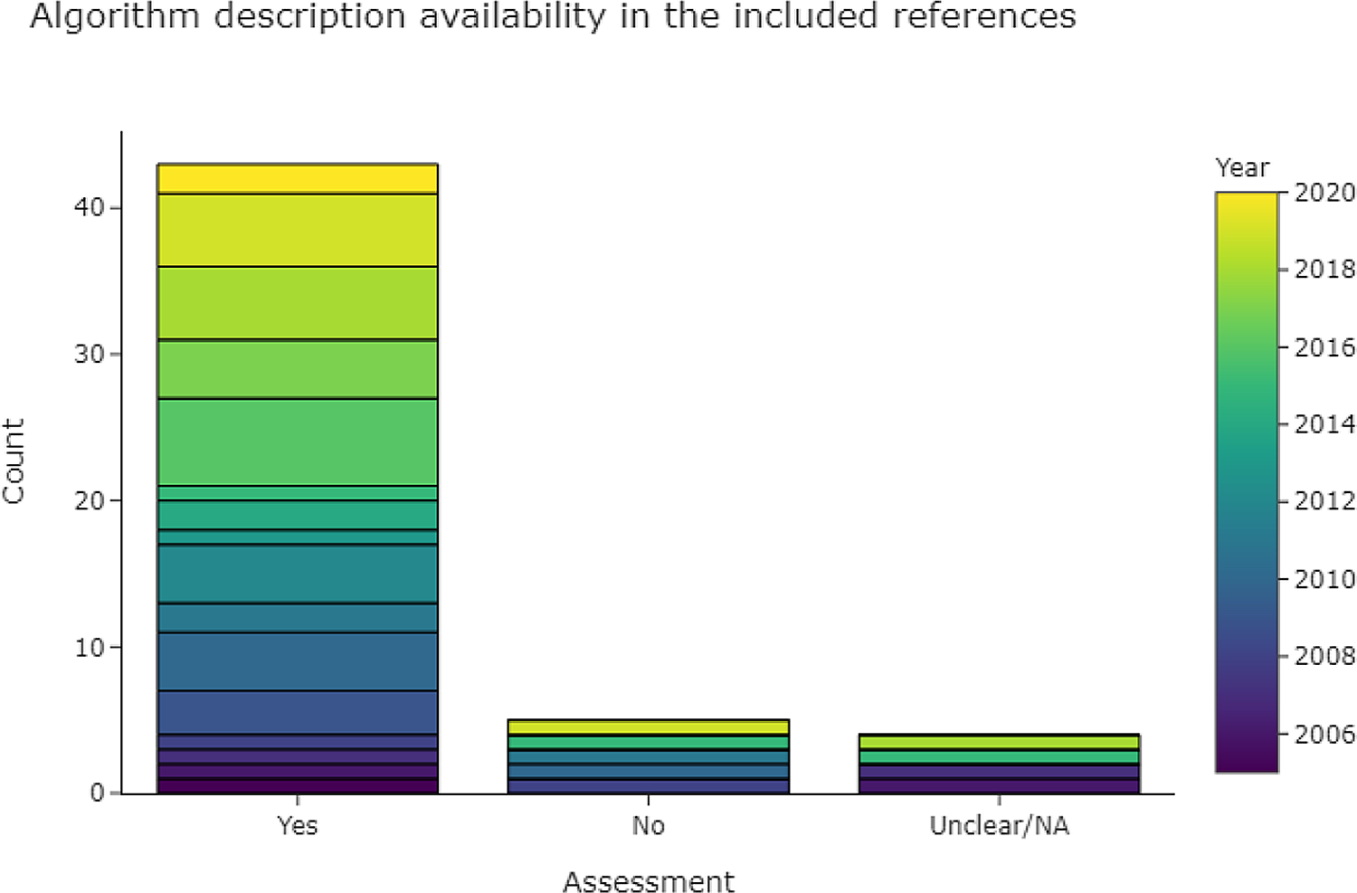
Bar chart, showing the levels of algorithm description in the included publications.

Figure 8 shows that 43 out of 53 publications (81%) provided descriptions of their data extraction algorithm. In the case of machine learning and neural networks, we looked for a description of hyperparameters and feature generation, and for the details of implementation (e. g. the machine-learning framework). Hyperparameters were rarely described in full, but if the framework (e.g., Scikit-learn, Mallet, or Weka) was given, in addition to a description of implementation and important parameters for each classifier, then we rated the algorithm as fully described. For rule-based methods we looked for a description of how rules were derived, and for a list of full or representative rules given as examples. Where multiple data extraction approaches were described, we gave a positive rating if the best-performing approach was described.

3.4.2.2 Is there a description of the dataset used and of its characteristics?

Of the included publications, 50 out of 53 (94%) provided descriptions of their dataset and its characteristics.

Most publications provided descriptions of the dataset(s) used for training and evaluation. The size of each dataset, as well as the frequencies of classes within the data, were transparent and described for most included publications. All datasets, along with a short description and availability of the data, are shown in Table 4.

**Table 4.** Corpora used in the included publications. RCT, randomized controlled trials; IR, information retrieval; PICO, population, intervention, comparison, outcome; UMLS, unified medical language system.

Publication	Also used by	Description	Classes	Size/type	Availability	Note
**^69^**	^60, 68, 46^	Automatically labelled sentence labels from structured abstracts up to Aug’17	P, IC, O, Method	24,668 abstracts	Yes	
**^47^**	^58, 68, 74^	Entities	P, IC, O + age, gender, and more entities	5,000 abstracts	Yes	
**^70^**		Entities	I and dosage-related	694 abstract/full text	Yes	Domain drug-based interventions
**^42^**		Entities	P, O, Design, Exposure	60 + 30 abstracts	Yes	Domain obesity
**^48^**		Sentence level 90,000 distant supervision annotations, 1000 manual.	Target condition, index test and reference standard	90,000 + 1000 sentences	Yes (labels, not text)	Domain diagnostic tests
**^44^**	^35^ (includes classifiers from ^75– 78^), ^24, 45, 46, 43^	Structured and unstructured abstracts, multi-label on sentences.	P, IC, O, Design	1000 abstracts	Yes	Multi-label sentences
**^41^**		Sentences	Drug intervention and comparative statements for each arm	300 (500 in available data) sentences	Yes	Domain drug-based interventions
**^64^**	^79^	Sentences and entities	P, N, sections	3657 structured abstracts with sentence tags, 204 abstracts with N (total) entities	No	
**^32^**		Structured, auto-labelled RCT abstracts with sentence tags and 378 documents with entity-level IR query-retrieval tags	P, IC, O	15,000 abstracts + 378 documents with IR tags	No	
**^57^**	^56^(unclear)	Sentences and entities	IC, O, N (total + per arm)	263 abstracts	No	
**^49^**	^33, 45^	100 abstracts with P, Condition, IC, possibly on entity level. For O, 633 abstracts are annotated on sentence level.	P, Condition, IC, 0	633 abstracts for O, 100 for other classes	No	
**^51^**		Entities	Age, Design, Setting (Country), IC, N, study dates and affiliated institutions	185 full texts (at least 93 labelled)	No	
**^53^**		Sentences and entities	P, IC, Age, Gender, Design, Condition, Race	2000 sentences from abstracts	No	
**^66^**		200 abstracts, 140 contain sentence and entity labels	P, IC	200 abstracts	No	
**^80^**		Auto-labelled structured abstracts, sentence level.	P, IC, O	14200+ abstracts	No	
**^67^**		Entities	P, age, gender, race	50 abstracts	No	
**^81^**		Sentences (and entities?)	P, IC, O	3000 abstracts	No	
**^28^**		Entities	N (total)	648 abstracts	No	
**^63^**		Entities	IC	330 abstracts	No	
**^37^**		Indonesian text with sentence annotations	P,I,C,O	200 abstracts	No	
**^38^**		Sentences from 69 (heart) +24 (random) RCTs included in Cochrane reviews	Inclusion criteria	69 + 24 full texts	No	Domain cardiology
**^54^**		Sentences and entities	P, IC, Age, Gender, P (Condition or disease)	200 abstracts	No	
**^40^**		4,824 sentences from 18 UpToDate documents and 714 sentences from MEDLINE citations for P. For I: CLEF 2013 shared task, and 852 MEDLINE citations	P, IC, P (Condition or disease)	abstracts, full texts	No	General topic and cardiology domain
**^27^**	^72^	Entity annotation as noun phrases	O, IC	100 + 132 sentences from full texts	No	Diabetes and endocrinology journals as source
**^65^**	^73^	Auto-labelled structured RCT abstract sentences. ^65^ has 19,854 sentences, assumed same corpus as authors and technique are the same.	P, IC, O	23,472 abstracts	No	
**^30^**		RCTs abstracts and full texts: 132 + 50 articles	IC (per arm), IC (drug entities.), O (time point), O (primary or secondary outcome), N (total), Eligibility criteria, Enrolment dates, Funding org, Grant number, Early stopping, Trial registration, Metadata	132 + 50 abstracts and full texts	No	
**^59^**		Sentences and entities	P, IC, O, N (per arm + total)	48 full texts	No	
**^26^**		Studies from 5 systematic reviews on environmental health exposure, entities	P, O, Country, Exposure	Studies from 5 systematic reviews	No	Observational studies on environmental health exposure in humans
**^29^**		Labelled via supervised distant supervision. Full texts (~12500 per class), 50 + 133 manually annotated for evaluation.	P, IC, O	12700+ full texts	No	
**^62^**		Sentence labels, structured & unstructured abstracts. Manually annotated: 344 IC, 341 O, and 144 P and more derived by automatic labelling.	P, IC, O	344+ abstracts	No	
**^61^**		Entities	P, IC, O, O as "Instruments" or "Study Variables"	20 full texts/abstracts	No	
**^58^**		Entities (Brat, IOB format)	P, IC, O	170 abstracts	No	
**^34^**		Entities assigned to UMLS concepts (probably Cochrane corpus, size unclear). '88 instances, annotated in total with 76, 87, and 139 [P, IC, O respectively]'	P, IC, O	Unclear, at least 88 documents	No	
**^25^**		Sentences and entities	P, IC (per arm), N (total)	1750 title or abstracts	No	
**^82^**		Excluded paper, no data extraction system. Corpus of Patient, Population, Problem, Exposure, Intervention, Comparison, Outcome, Duration and Results sentences in abstracts.			No	Excluded from review, but describes relevant corpus
**^31^**		Sentences and entities	P, IC (per arm), O, multiple more	88 full texts	No	

3.4.2.3 Is there a description of the hardware used?

Most included publications did not report their hardware specifications, though five publications (9%) did. One, for example, applied their system to new, unlabelled data and reported that classifying the whole of PubMed takes around 20 hours using a graphics processing unit (GPU). ^39^ In another example, the authors reported using Google Colab GPUs, along with estimates of computing time for different training settings. ^68^

3.4.2.4 Is the source code available?

**Figure 9.**
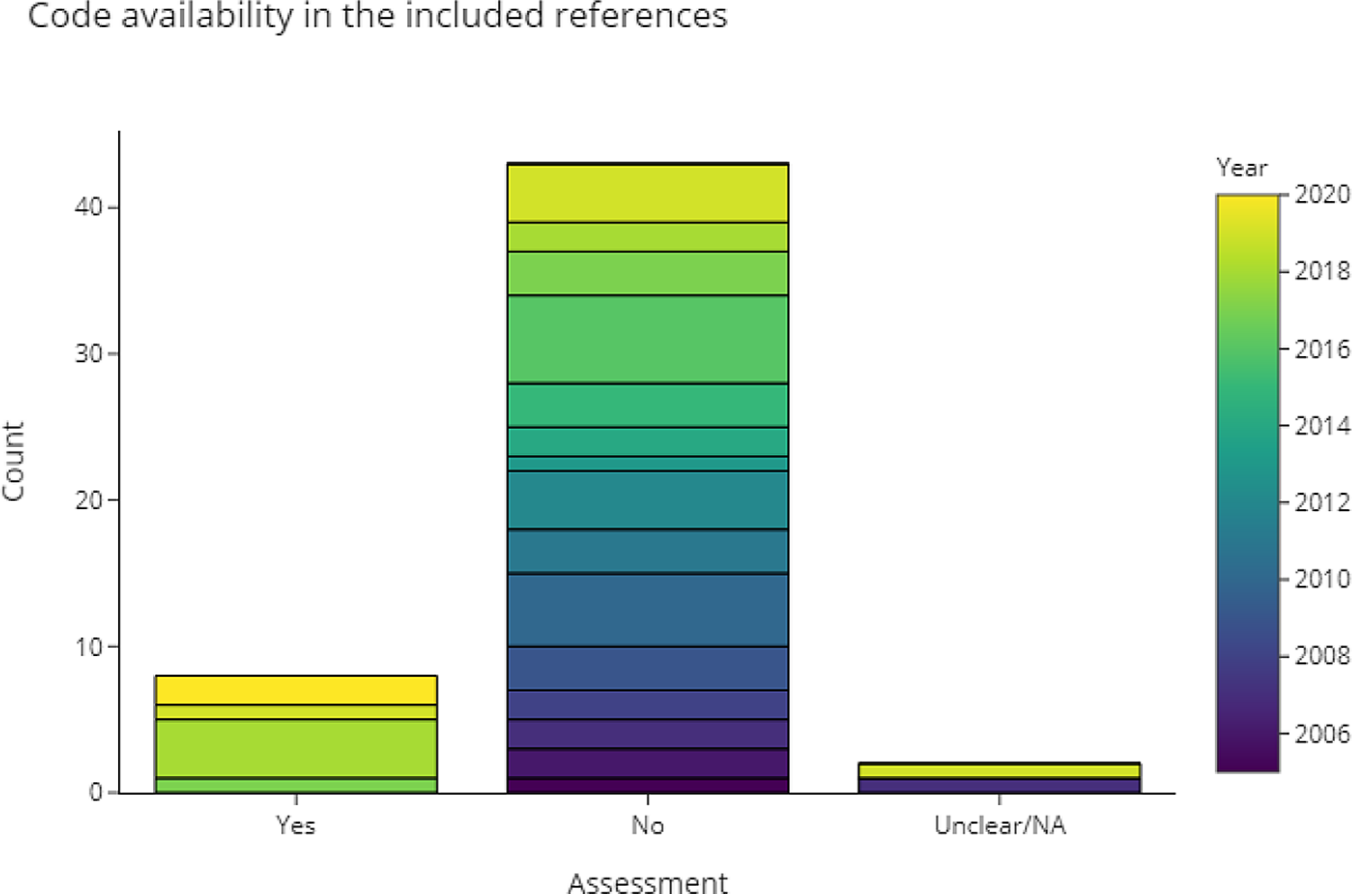
This chart shows the extent to which included publications provided access to their source code.

Figure 9 shows that most of the included publications did not provide any source code. Publications that did provide the source code were exclusively published or last updated in the last five years (n = 8). GitHub is the most popular platform for making code accessible. Some publications also provided links to notebooks on Google Colab, which is a cloud-based platform to develop and execute code online. Two publications provided access to parts of the code, or access was restricted. A full list of code repositories from the included publications is available in Table 2.

**Table 2.** Repositories containing source code for the included publications.

Publication	Code
^55^	Available under: https://github.com/ijmarshall/robotreviewer, older version: https://figshare.com/articles/Spa/997707
^69^	Available under: https://github.com/jind11/LSTM-PICO-Detection
^47^	Available under: https://github.com/bepnye/EBM-NLP https://colab.research.google.com/drive/1Ir52OmkJ2C_Iy9V_eS-_KFVLircJ4MXp https://colab.research.google.com/drive/1YbbQojM147Ybt1nEcyoXTqlvefmwMg-q
^46^	Available under: https://github.com/jind11/Deep-PICO-Detection
^71^	Available under: https://ii.nlm.nih.gov/DataSets/index.shtml
^58^	Available under: https://github.com/Tian312/PICO_Parser
	
^68^	Available under: https://github.com/L-ENA/HealthINF2020 https://www.kaggle.com/lenaschmidt0493/qa-integrated-biomedical-ner-classifier-for-pico
^39^	Available under: https://github.com/ijmarshall/trialstreamer
^41^	Unclear if Java code is accessible, pending user access: https://semrep.nlm.nih.gov/SemRep.v1.8_Installation.html#Download
^48^	Used public Google implementation of transformers + https://zenodo.org/record/1303259#.X4wSoaySk2w

***3.4.3 Testing***

3.4.3.1 Is there a justification/an explanation of the model assessment?

Of the included publications, 47 out of 53 (89%) gave a detailed assessment of their data extraction algorithms. We rated this item as negative if only the performance scores were given, i.e., if no error analysis was performed and no explanations or examples were given to illustrate model performance. In most publications a brief error analysis was common, for example discussions on representative examples for false negatives and false positives, ^41^ major error sources ^63^ or highlighting errors with respect to every entity class. ^49^ Both ^44, 45^ used structured and unstructured abstracts, and therefore discussed the implications of unstructured text data for classification scores.

A small number of publications did a real-life assessment, where the data extraction algorithm was applied to different, unlabelled, and often much larger datasets or tested while conducting actual systematic reviews. ^30, 33, 39, 42, 68, 71, 72^

3.4.3.2 Are basic metrics reported (true/false positives and negatives)?

**Figure 10.**
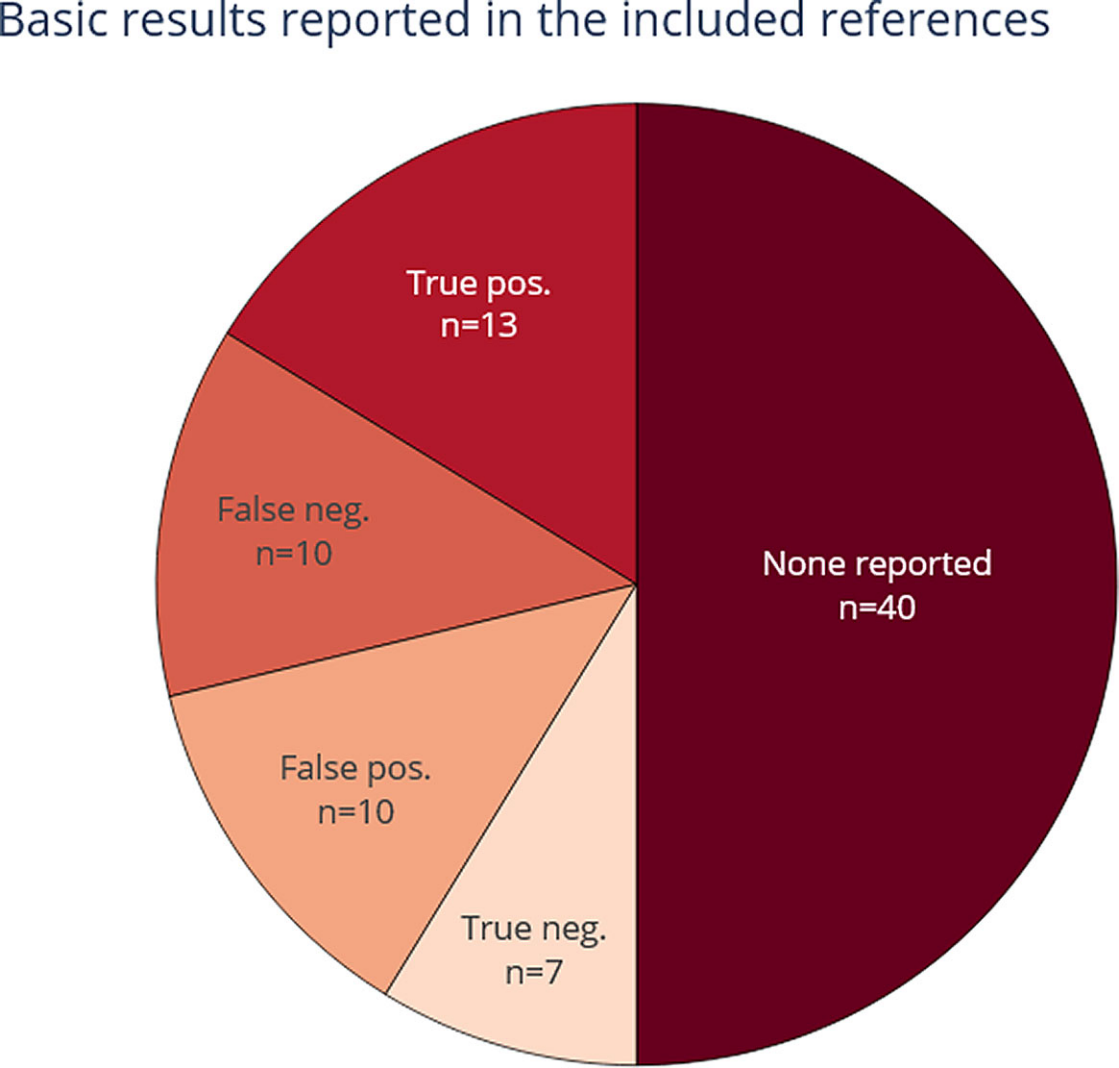
Reporting of basic metrics (true positive, false positive, true negative, and false negative). For each included paper. More than one selection is possible, which means that the total number of included publications (n=53) is lower than the sum of counts within this figure.

Figure 10 shows the extent to which all basic metrics were reported in the included publications. In most publications (n = 40) these basic metrics are not reported. When dealing with entity-level data extraction it can be challenging to define the quantity of true negative entities. This is true especially if entities are labelled and extracted based on text chunks, because there can be many combinations of phrases and tokens that constitute an entity. ^41^ This problem was solved in more recent publications by conducting a token-based evaluation that computes scores across every single token, hence gaining the ability to score partial matches for multi-word entities. ^47^

3.4.3.3 Does the assessment include any information about trade-offs between recall or precision (also known as sensitivity and positive predictive value)?

Of the included publications, 17 out of 53 (32%) described trade-offs or provided plots or tables showing the development of evaluation scores if certain parameters were altered or relaxed. Recall (i.e., sensitivity) is often described as the most important metric for systematic review automation tasks, as it is a methodological demand that systematic reviews do not exclude any eligible data.

^49, 31^ showed how the decision of extracting the top two or N predictions impacts the evaluation scores, for example precision or recall. ^72^ show precision-recall plots for different classification thresholds. ^50^ show four cut-offs, whereas ^68^ show different probability thresholds for their classifier, and describe the impacts of this on precision, recall, and F1 curves.

Some machine-learning architectures need to convert text into features before performing classification. A feature can be, for example, the number of times that a certain word occurs, or the length of an abstract. The number of features used, e. g. for CRF algorithms, which was given in multiple publications, ^65^ together with a discussion of classifiers that should be used in high recall is needed ^73^. ^28^ show ROC curves quantifying the amount of training data and its impact on the scores.

***3.4.4 Availability of the final model or tool***

3.4.4.1 Can we obtain a runnable version of the software based on the information in the publication?

**Figure 11.**
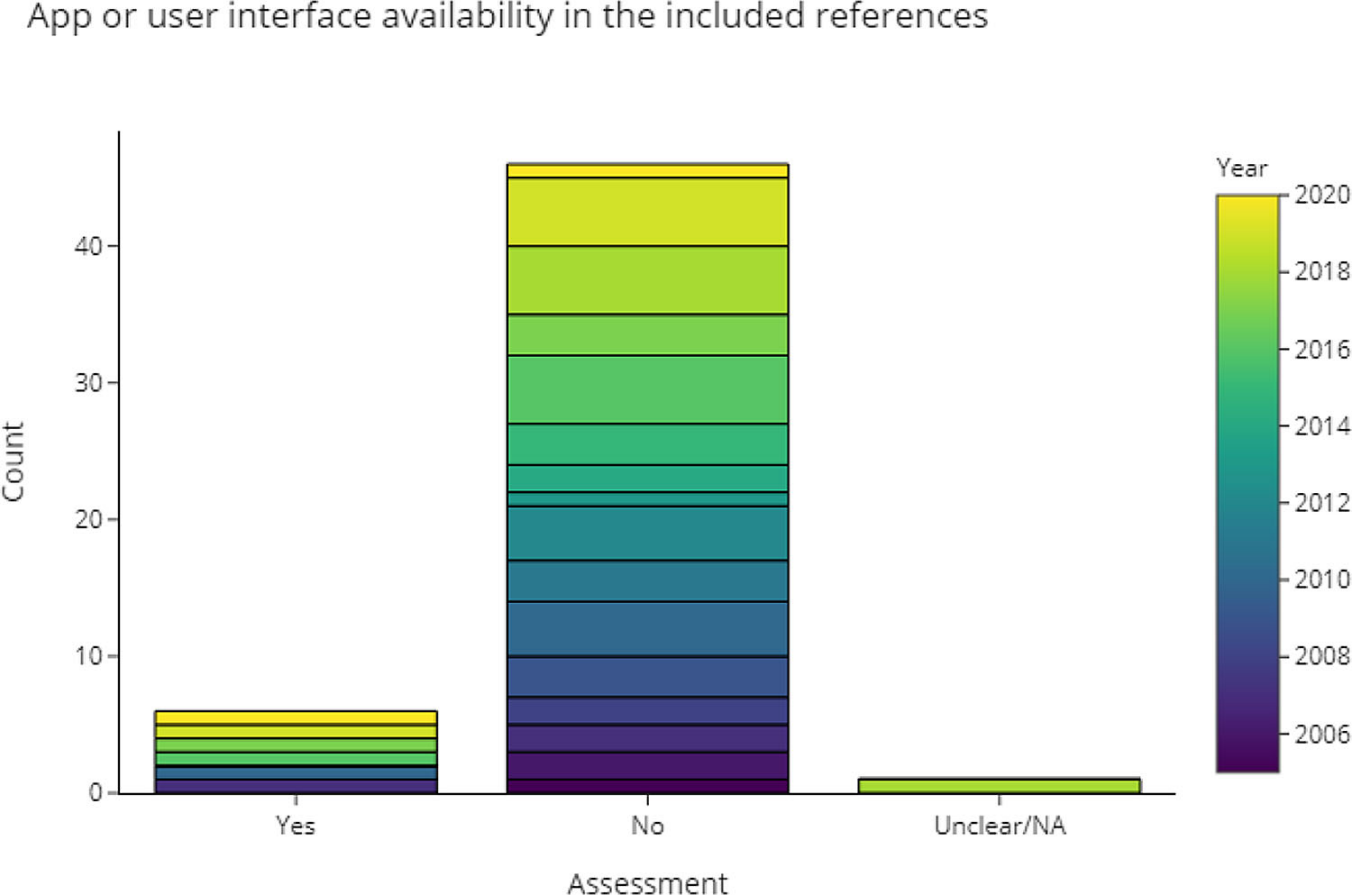
Publications that provide applications with user interface.

Compiling and testing code from every publication is outside the scope of this review. Instead, in Figure 11 and Table 3 we recorded the publications where a (web) interface or finished application was available. Counting RobotReviewer and Trialstreamer as separate projects, 9% of the included publications had an application associated with it.

**Table 3.** Publications that provide user interfaces to their final data extraction system.

Paper	Access
^28^	Unclear: A link was given, but tool is not yet online: https://ihealth.uemc.es/
^25^	https://www.tripdatabase.com/#pico
^29, 55^	https://www.robotreviewer.net/
^30^	https://exact.cluster.gctools.nrc.ca/ExactDemo/
^41^	https://semrep.nlm.nih.gov/SemRep.v1.8_Installation.html , SemMed is a web-based application published after this publication was released: https://skr3.nlm.nih.gov/SemMed/semmed.html
^39^	Database with all extracted data is available online: https://trialstreamer.robotreviewer.net/
^33^	Pending: article mentions that an app is being implemented.

3.4.4.2 Persistence: Can data be retrieved based on the information given in the publication?

Seven of the included publications (13%) made their datasets publicly available. Whereas 17 publications (32%) reported using at least one dataset published elsewhere. Of those 17, datasets used in six publications were not publicly available, but in these cases, there were often overlaps of at least one author in the author teams, explaining facilitated access to data. A further 29 (55%) publications appeared to have curated their own datasets.

In total, we counted 36 unique corpora with labelled data. Table 4 shows a summary of the corpora, their size, classes, and cross-reference to known publications re-using each data set. Where available, we collected the corpora, provide a central link to all datasets, and will add datasets as they become available during the life span of this living review (see *Underlying data* ^86, 87^ below). When a dataset is made freely available without barriers (i.e., direct downloads of text and labels), then large numbers of researchers can re-use the data and publish results from different models, which become comparable to one another. Copyright issues surrounding data sharing were noted by, ^48^ therefore they shared the gold-standard annotations used as training or evaluation data and information on how to obtain the texts.

3.4.4.3 Is the use of third-party frameworks reported and are they accessible?

Of the included publications, 47 out of 53 (88%) described using at least one third-party framework for their data extraction systems. The following list is likely to be incomplete, due to non-available code and incomplete reporting in the included publications. Most commonly, there was a description of machine-learning toolkits (Mallet, N = 12; Weka, N = 6; tensorflow, N = 5; scikit-learn, N = 3). Natural language processing toolkits such as Stanford parser/CoreNLP (N = 12) or NLTK (N = 3), were also commonly reported for the pre-processing and dependency parsing steps within publications. The MetaMap tool was used in nine publications, and the GENIA tagger in four. For the complete list of frameworks please see Appendix A in *Underlying data*. ^86^

***3.4.5 Internal and external validity of the model***

3.4.5.1 Does the dataset or assessment measure provide a possibility to compare to other tools in the same domain?

With this item we aimed to assess publications to see if the evaluation results from models are comparable with the results from other models. Ideally, a publication would have reported the results of another classification model on the same dataset, either by re-implementing the model themselves ^69^ or by describing results of other models when using benchmark datasets. ^35^ This was rarely the case for the publications in this review, as most datasets were curated and used in single publications only (see Table 4).

However, in 40 publications (75%) data were well described, and they utilised commonly used entities and common assessment metrics, such as precision, recall, and F1-scores, leading to a limited comparability of results. In these cases, the comparability is limited because those publications used different data sets, which can influence the difficulty of the data extraction task and lead to better results within for example structured datasets or topic-specific datasets.

3.4.5.2 Are explanations for the influence of both visible and hidden variables in the dataset given?

This item relates only to publications using machine learning or neural networks. Rule-based classification systems (N = 8, 15% reporting rule-base as sole approach) are not applicable to this item, because the rules leading to decisions are intentionally chosen by the creators of the system and are therefore always visible.

Ten publications (19%) discussed hidden variables. ^56^ discussed that the identification of the treatment group entity yielded the best results. However, when neither the words ‘group’ nor ‘arm’ were present in the text then the system had problems with identifying the entity. ‘Trigger tokens’ ^43^ and the influence of common phrases were also described by, ^38^ the latter showed that their system was able to yield some positive classifications in the absence of common phrases. ^73^ went a step further and provided a table with words that had the most impact on the prediction of each class. ^32^ describes removing sentence headings in structured abstracts in order to avoid creating a system biased towards common terms, while ^63^ discussed abbreviations and grammar as factors influencing the results. Length of input text ^34^ and position of a sentence within a paragraph or abstract, e. g. up to 10% lower classification scores for certain sentence combinations in unstructured abstracts, were shown in several publications. ^30, 37, 72^

3.4.5.3 Is the process of avoiding overfitting or underfitting described?

‘Overfitted’ is a term used to describe a system that shows particularly good evaluation results on a specific dataset because it has learned to classify noise and other intrinsic variations in the data as part of its model. ^83^

Of the included publications, 33 out of 53 (62%) reported that they used methods to avoid overfitting. Eight (15%) of all publications reported rule-based classification as their only approach, allowing them to not be susceptible to overfitting by machine learning.

Furthermore, 28 publications reported cross-validation to avoid overfitting. Mostly these classifiers were in the domain of machine-learning, e. g. SVMs. Most commonly, 10 folds were used (N = 15), but depending on the size of evaluation corpora, 3, 6, 5 or 15 folds were also described. Two publications ( ^58, 47^) cautioned that cross-validation with a high amount of folds (e. g. 10) causes high variance in evaluation results when using small datasets such as NICTA-PIBOSO. One publication ^43^ stratified folds by class in order to avoid this variance in evaluation results in a fold which is caused by a sparsity of positive instances.

Publications in the neural and deep-learning domain described approaches such as early stopping, dropout, L2-regularisation, or weight decay. ^34, 69, 74^ Some publications did not specifically discuss overfitting in the text, but their open-source code indicated that the latter techniques were used. ^47, 48^

3.4.5.4 Is the process of splitting training from validation data described?

Random allocation to treatment groups is an important item when assessing bias in RCTs, because selective allocation can lead to baseline differences. ^1^ Similarly the process of splitting a dataset randomly, or in a stratified manner, into training (or rule-crafting) and test data is important when constructing classifiers and intelligent systems. ^84^

All included publications gave indications of how different train and evaluation datasets were obtained. Most commonly there was one dataset and the splitting ratio which indicated that splits were random. This information was provided in 36 publications (68%).

For publications mentioning cross-validation (N = 28, 53%) we assumed that splits were random. The ratio of splitting (e.g. 80:20 for training and test data) was clear in the cross-validation cases and was described in the remainder of publications.

It was also common for publications to use completely different datasets, or multiple iterations of splitting, training and testing (N = 13, 24%). For example, ^31^ used cross-validation to train and evaluate their model, and then used an additional corpus after the cross-validation process. Similarly, ^34^ used 60:40 train/test splits, but then created an additional corpus of 88 documents to further validate the model’s performance on previously unseen data.

3.4.5.5 Is the model’s adaptability to different formats and/or environments beyond training and testing data described?

For this item we aimed to find out how many of the included publications tested their data extraction algorithms on different datasets. A limitation often noted in the literature was that gold-standard annotators have varying styles and preferences, and that datasets were small and limited to a specific literature search. Evaluating a model on multiple independent datasets provides the possibility of quantifying how well data can be extracted across domains and how flexible a model is in real-life application with completely new data sets. Of the included publications, 19 (36%) discussed how their model performed on datasets with characteristics that were different to those used for training and testing. In some instances, however, this evaluation was qualitative where the models were applied to large unlabelled, real-life datasets. ^30, 33, 39, 42, 68, 71, 72^

***3.4.6 Other***

3.4.6.1 Caveats

Caveats were extracted as free text. Included publications (N = 47, 87%) reported a variety of caveats. After extraction we structured them into six different domains:

1. Label-quality and inter-annotator disagreements
2. Variations in text
3. Domain adaptation and comparability
4. Computational or system architecture implications
5. Missing information in text or knowledge base
6. Practical implications

These are further discussed in the ‘Discussion’ section of this living review.

3.4.6.2 Sources of funding and conflict of interest

**Figure 12.**
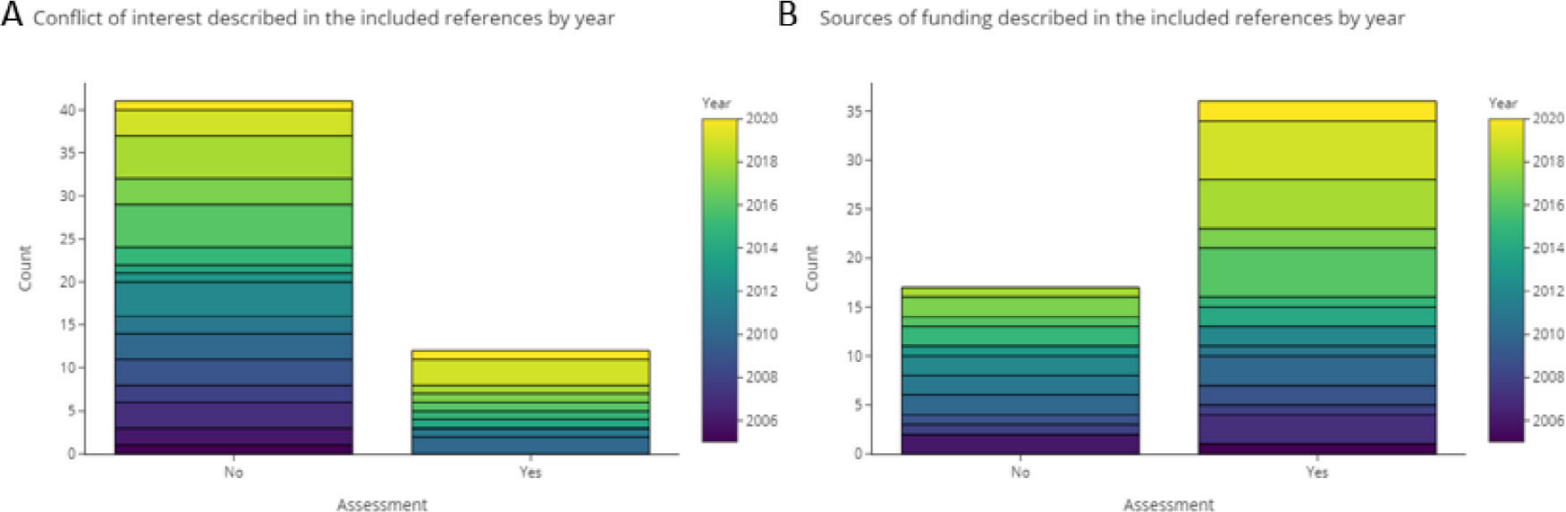
Declaration of funding sources and conflict of interest in the included studies.

Figure 12 shows that most of the included publications did not declare any conflict of interest. This is true for most publications published before 2010, and about 50% of the literature published in more recent years. However, sources of funding were declared more commonly, with 69% of all publications including statements for this item. This reflects a trend of more complete reporting in more recent years.

## 4. Discussion

### 4.1 Summary of key findings

***4.1.1 System architectures***

Systems described within the included publications are changing over time. Non-machine-learning data extraction via rule-base and API is one of the earliest and most frequently used approaches. Various classical machine-learning classifiers such as naïve Bayes and SVMs are very common in the literature published between 2005-2018, but in the current literature there is a trend towards word embeddings and neural networks such as LSTMs and transformers.

***4.1.2 Evaluation***

We found that precision, recall, and F1 were used as evaluation metrics in most publications, although sometimes these metrics were adapted or relaxed in order to account for partial or similar matches.

***4.1.3 Scope***

Most of the included publications focused on extracting data from abstracts. The reasons for this include the availability of data and ease of access, as well as the high coverage of information and the availability of structured abstracts that can automatically derive labelled training data. A much smaller number of the included publications (n=14, 26%) extracted data from full texts. Half of the systems that extract data from full text were published within the last five years. In systematic review practice, manually extracting data from abstracts is quicker and easier than manually extracting data from full texts. Therefore, the potential time saving and utility of full text data extraction is much higher because more time can be saved by automation, but the data extraction literature on that topic is still sparse.

***4.1.4 Target texts***

Reports of randomised controlled trials were the most common texts used for data extraction. Evidence concerning data extraction from other study types was rare and is discussed further in the following sections.

### 4.2 Assessment of the quality of reporting

The quality of reporting in the included studies is improving over time, especially in terms of the availability of data and source code. We assessed the included publications based on a list of 17 items in the domains of reproducibility, transparency, description of testing, data availability, and internal and external validity.

Reproducibility was high throughout, with sources of training and evaluation data reported in 94% of all publications and pre-processing described in 89%.

In terms of transparency, 81% of the publications provided a clear description of their algorithm, 94% described the characteristics of their datasets, but only 9% mentioned hardware specifications or feasibility of using their algorithm on large real-world datasets such as PubMed. Availability of source code was low (15%) and was only observed in recent publications.

Testing of the systems was generally described, 89% gave a detailed assessment of their algorithms. Basic metrics were reported in 24%, trade-offs between precision and recall were discussed in 32%.

Availability of the final models and tools was very poor. We found that only 11% of all publications are based on tools that are available and have a graphical user interface, and it is unclear how many of the other tools described in the literature are used in practice. Labelled training and evaluation data are available from 13% of the publications, but only a further 32% of all publications reported using one of these available datasets. A total of 88% of the publications described using at least one accessible third-party framework for their data extraction system.

Internal and external validity of each model was assessed based on its comparability to other tools (75%), assessment of visible and hidden variables in the data (19%), avoiding overfitting (62%, not applicable to non-machine learning systems), descriptions of splitting training from validation data (100%), and adaptability and external testing on datasets with different characteristics (36%). These items, together with caveats and limitations noted in the included publications are discussed in the following section.

### 4.3 Caveats and challenges for systematic review (semi)automation

In the following section we discuss caveats and challenges highlighted by the authors of the included publications. We found a variety of topics discussed in these publications and summarised them under seven different domains.

***4.3.1 Label-quality and inter-annotator disagreements***

**Figure 13.**
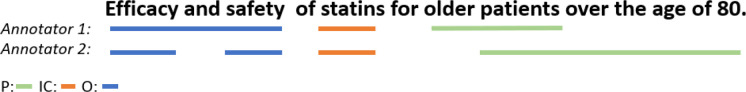
Example of inter-annotator disagreement. P, population; I, intervention; C, comparison; O, outcome.

The quality of labels in annotated datasets was identified as a problem by several authors. The length of the entity being annotated, for example O or P entities, often caused disagreements between annotators. ^30, 33, 39, 42, 68, 71, 72^ We created an example in Figure 13, which shows two potentially correct, but nevertheless different annotations on the same sentence.

Similar disagreements, ^36, 43, 58^ along with missed annotations, ^50^ are time-intensive to reconciliate ^70^ and make the scores less reliable. ^68^ As examples of this, two publications observed that their system performed worse on classes with high disagreement. ^43, 48^ There exist different explanations for worse performance in these cases. It is possibly harder for models to learn from labelled data with systematic differences within. Another reason is that the model learns predictions based on one annotation style and therefore artificial errors are produced when evaluated against differently labelled data, or that the annotation task itself is naturally harder in cases with high inter-annotator disagreement, and therefore lower performance from the models might be explainable. An overview of the included publications discussing this, together with their inter-annotator disagreement scores, is given in Table 5.

To mitigate these problems, careful training and guides for expert annotators are needed. ^33, 51^ For example, information should be provided on whether multiple basic entities or one longer entity annotation are preferred. ^58^ Crowd-sourced annotations can contain noisy or incorrect information and have low interrater reliability. However, they can be aggregated to improve quality. ^47^ In recent publications, partial entity matches (i.e., token-wise evaluation) downstream were generally favoured above complete detection, which helps to mitigate this problem’s impact on final evaluation scores. ^47, 56^

For automatically labelled or distantly supervised data, label quality is generally lower. This is primarily caused by incomplete annotation due to missing headings, or by ambiguity in sentence data, which is discussed as part of the next domain. ^29, 32, 73^

***4.3.2 Ambiguity***

The most common source of ambiguity in labels described in the included publications is associated with automatically labelled sentence-level data. Examples of this are sentences that could belong to multiple categories, e.g., those that should have both ‘P’ and an ‘I’ label, or sentences that were assigned to the class ‘other’ while containing PICO information ( ^46, 68, 69^, among others). Ambiguity was also discussed with respect to intervention terms ^49^ or when distinguishing between ‘control’ and ‘intervention’ arms. ^30^ When using, or mapping to UMLS concepts, ambiguity was discussed in. ^27, 44, 50^

At the text level, ambiguity around the meaning of specific wordings was discussed as a challenge, e.g., the word 'concentration' can be a quantitative measure or a mental concept. ^27^ Numbers were also described as challenging due to ambiguity, because they can refer to the total number of participants, number per arm of a trial, or can just refer to an outcome-related number. ^57, 79^ When classifying participants, the P entity or sentence is often overloaded because it includes too much information on different, smaller, entities within it, such as age, gender, or diagnosis. ^62^

***4.3.3 Variations in text***

Variations in natural language, wording, or grammar were identified as challenges in many references that looked closer at the texts within their corpora. Such variation may arise when describing entities or sentences (e.g., ^42, 53, 70^) or may reflect idiosyncrasies specific to one data source, e.g., the position of entities in a specific journal. ^30^ In particular, different styles or expressions were noted as caveats in rule-based systems. ^28, 42, 54^

There is considerable variation in how an entity is reported, for example between intervention types (drugs, therapies, routes of application) ^31^ or in outcome measures. ^30^ In particular, variations in style between structured and unstructured abstracts ^36, 52^ and the description lengths and detail ^34, 53^ can cause inconsistent results in the data extraction, for example by not detecting information correctly or extracting unexpected information. Complex sentence structure was mentioned as a caveat especially for rule-based systems. ^54^ An example of a complex structure is when more than one entity is described (e.g., ^66, 72^) or when entities such as ‘I’ and ‘O’ are mentioned close to each other. ^32^ Finally, different names for the same entity within an abstract are a potential source of problems. ^57^

Another common variation in text was implied information. For example, rather than stating dosage specifically, a trial text might report dosages of '10 or 20mg', where the ‘mg’ unit is implied for the number 10, making it a ‘dosage’ entity. ^30, 42, 63^

***4.3.4 Domain adaptation and comparability***

Because of the wide variation across medical domains, there is no guarantee that a data extraction system developed on one dataset automatically adapts to produce reliable results across different datasets relating to other domains. The hyperparameter configuration or rule-base used to conceive a system may not retrieve comparable results in a different medical domain. ^24, 38^ Therefore, scores might not be similar between different datasets, especially for rule-based classifiers, ^54^ when datasets are small, ^26^ when structure and distribution of class of interest varies, ^24^ or when the annotation guidelines vary. ^58^ Another caveat mentioned by ^58, 34^ is that the size of the label space must be considered when comparing scores, as models that normalise to specific concepts rather than detecting entities tend to have lower precision, recall, and F1 scores.

Finally, several publications discuss that a larger amount of benchmarking datasets could increase the comparability between published systems. ^30, 65, 80^

***4.3.5 Computational or system architecture implications***

Computational cost and scalability were described in two publications. ^45, 80^ Problems within the system, e.g., encoding ^70^ or PDF extraction errors ^48^ lead to problems downstream and ultimately result in bias, favouring articles from big publishers with better formatted data. ^48^ Similarly, grammar and parsing part-of-speech and/or chunking errors ( ^49, 54, 63^, among others) or faulty parse-trees ^52^ can reduce a system’s performance if it relies on access to correct grammatical structure. In terms of system evaluation, 10-fold cross-validation causes high variance in results when using small datasets such as NICTA-PIBOSO, ^46, 58^ and ^43^ described that the same problem needs to be addressed through stratification of the positive instances of each class within folds.

***4.3.6 Missing information in text or knowledge base***

Information in text can be incomplete. ^80^ For example, the number of patients in a study might not be explicitly reported, ^49^ or abstracts lacking information about study design and methods can appear, especially in unstructured abstracts and older trial texts. ^64, 66^ In some cases, abstracts can be missing entirely. These problems can sometimes be solved by considering the use of full texts as input. ^40, 60^

Where a model relies on features, e.g., MetaMap, then missing UMLS coverage causes errors. ^49, 50^ This also applies to models like CNNs that assign specific concepts, where unseen entities are not defined in the output label space. ^34^

***4.3.7 Practical implications***

In contrast to the problem of missing information, too much information can also have practical implications. For instance, often there are multiple sentences with each label, of which one is ‘key’, e.g., the descriptions of inclusion and exclusion criteria often span multiple sentences, and for a data extraction system it can be challenging to work out which sentence is the key sentence. The same problem applies to methods that select and rank the top-n sentences for each data extraction target, where a system risks including too much, or not enough results depending on the amount of sentences that are kept. ^30^

Low recall is an important practical implication, ^45^ especially in entities that appear infrequently in the training data, and are therefore not well represented in the training process of the classification system. ^42^ In other words, an entity such as ‘Race’ might not be labelled very often is a training corpus, and systematically missed or wrongly classified when the data extraction system is used on new texts. Therefore, human involvement is needed, ^59^ and scores need to be improved. ^27^ It is challenging to find the best set of hyperparameters ^74^ and to adjust precision and recall trade-offs to maximise the utility of a system while being transparent about the number of data points that might be missed when increasing system precision to save work for a human reviewer. ^39, 68, 71^

### 4.4 Explainability and interpretability of data extraction systems

The neural networks or machine-learning models from publications included in this review learn to classify and extract data by adjusting numerical weights and by applying mathematical functions to these sets of weights. The decision-making process behind the classification of a sentence or an entity is therefore comparable with a black box, because it is very hard to comprehend how, or why the model made its predictions. A recent comment published in Nature has called for a more in-depth analysis and explanation of the decision-making process within neural networks. ^84^ Ultimately, hidden tendencies in the training data can influence the decision-making processes of a data extraction model in a non-transparent way. Many of the examples discussed in the comment are related to healthcare, but in practice there is a very limited understanding of their inherent biases despite the broad application of machine learning and neural networks. ^84^

A deeper understanding of what occurs between data entry and the point of prediction can benefit the general performance of a system, because it uncovers shortcomings in the training process. These shortcomings can be related to the composition of training data (e. g. overrepresentation or underrepresentation of groups), the general system architecture, or to other unintended tendencies in a system’s prediction. ^85^ A small number of included publications (N = 10) discussed issues related to hidden variables as part of an extensive error analysis (see section 3.5.2). The composition of training and testing data were described in most publications, but no publication that specifically addresses the issues of interpretability or explainability was found.

### 4.5 Availability of corpora, and copyright issues

There are several corpora described in the literature, many with manual gold-standard labels. However, the number of shared datasets remains low (see Table 4). Possible reasons for this are concerns over copyright, or malfunctioning download links from websites mentioned in older publications. Ideally, data extraction algorithms should be evaluated on different datasets in order to detect over-fitting, to test how the systems react to data from different domains and different annotators, and to enable the comparison of systems in a reliable way. As a supplement to this manuscript, we have created an online repository for available datasets, and encourage researchers to share their automatically or manually annotated labels and texts so that other researchers may use them for development and evaluation of new data extraction systems.

### 4.6 Limitations of this living review

This review focused on data extraction from reports of clinical trials and epidemiological research. This mostly includes data extraction from reports of randomised controlled trials, and only a very small fraction of the evidence that addresses other important study types (e.g., diagnostic accuracy studies). During screening we excluded all publications related to clinical data (such as electronic health records) and publications extracting disease, population, or intervention data from genetic and biological research. There is a wealth of evidence and potential training and evaluation data in these publications, but it was not feasible to include them in the living review.

## 5. Conclusion

This living review presents an overview of the data-extraction literature of interest to different types of systematic review. We included a broad evidence base of publications describing data extraction for interventional systematic reviews (focusing on P, IC, and O classes and RCT data), and a very small number of publications extracting epidemiological and diagnostic accuracy data. However, the number of accessible tools that can help systematic reviewers with data extraction is very low. Currently, only around one in ten publications is linked to a usable tool or describes an ongoing implementation.

The data extraction algorithms and the characteristics of the data they were trained and evaluated on were well reported. However, only around one in ten publications made their datasets available to the public, and only a third of all included publications reported training or evaluating on these datasets. This makes it very difficult to draw conclusions on which is the best performing system. Additionally, data extraction is a very hard task. It usually requires conflict resolution between expert systematic reviewers when done manually, and consequently creates problems when creating the gold standards used for training and evaluation of the algorithms in this review.

We listed many ongoing challenges in the field of data extraction for systematic review (semi) automation, including ambiguity in clinical trial texts, incomplete data, and previously unseen data. With this living review we aim to review the literature continuously as it becomes available. Therefore, the most current review version, along with the number of abstracts screened and included after the publication of this review iteration, is available on our website.

## Data availability

### Underlying data

Harvard Dataverse: Appendix for base review. https://doi.org/10.7910/DVN/LNGCOQ. ^86^

This project contains the following underlying data:

• Appendix_A.zip (full database with all data extraction and other fields)
• Appendix B.docx (further information about excluded publications)
• Appendix_C.zip (code, weights, data, scores of abstract classifiers for Web of Science content)
• Supplementary_key_items.docx (overview of items extracted for each included study)
• table 1. csv and table 1_long.csv ( Table 1 in csv format, the long version includes extra data)
• included.ris and background.ris (literature references in this paper)

Harvard Dataverse: Available datasets for SR automation. https://doi.org/10.7910/DVN/0XTV25. ^87^

This project contains the following underlying data:

• Datasets shared by authors of the included publications

Data are available under the terms of the Creative Commons Zero “No rights reserved” data waiver (CC0 1.0 Public domain dedication).

### Extended data

Open Science Framework: Data Extraction Methods for Systematic Review (semi)Automation: A Living Review Protocol. https://doi.org/10.17605/OSF.IO/ECB3T. ^9^

This project contains the following extended data:

• Review protocol
• Additional_Fields.docx (overview of data fields of interest for text mining in clinical trials)
• Search.docx (additional information about the searches, including full search strategies)
• PRISMA P checklist for ‘Data extraction methods for systematic review (semi)automation: A living review protocol.’

Data are available under the terms of the Creative Commons Attribution 4.0 International license (CC-BY 4.0).

### Reporting guidelines

Harvard Dataverse: PRISMA checklist for ‘Data extraction methods for systematic review (semi)automation: A living systematic review’ https://doi.org/10.7910/DVN/LNGCOQ. ^86^

Data are available under the terms of the Creative Commons Zero “No rights reserved” data waiver (CC0 1.0 Public domain dedication).

## Software availability

The development version of the software for automated searching is available from Github: https://github.com/mcguinlu/COVID_suicide_living.

Archived source code at time of publication: http://doi.org/10.5281/zenodo.3871366 (14).

License: MIT

## Author contributions

LS: Conceptualization, Investigation, Methodology, Software, Visualization, Writing – Original Draft Preparation

BKO: Conceptualization, Investigation, Methodology, Software, Writing – Review & Editing

LAM: Conceptualization, Validation, Methodology, Software, Writing – Review & Editing

JT: Conceptualization, Investigation, Methodology, Writing – Review & Editing

JPTH: Conceptualization, Funding Acquisition, Investigation, Methodology, Writing – Review & Editing
