# Data extraction methods for systematic review (semi)automation: Update of a living systematic review

**DOI:** 10.12688/f1000research.51117.2

**Published:** 2023-10-09

**Authors:** Lena Schmidt, Ailbhe N. Finnerty Mutlu, Rebecca Elmore, Babatunde K. Olorisade, James Thomas, Julian P. T. Higgins

**Affiliations:** 1 NIHR Innovation Observatory, Newcastle University, Newcastle upon Tyne, NE4 5TG, UK; 2 Sciome LLC, Research Triangle Park, North Carolina, 27713, USA; 3 Bristol Medical School, University of Bristol, Bristol, BS8 2PS, UK; 4 UCL Social Research Institute, University College London, London, WC1H 0AL, UK; 5 Evaluate Ltd, London, SE1 2RE, UK; 6 Cardiff School of Technologies, Cardiff Metropolitan University, Cardiff, CF5 2YB, UK

**Keywords:** Data Extraction, Natural Language Processing, Reproducibility, Systematic Reviews, Text Mining

## Abstract

**Background:** The reliable and usable (semi)automation of data extraction can support the field of systematic review by reducing the workload required to gather information about the conduct and results of the included studies. This living systematic review examines published approaches for data extraction from reports of clinical studies.

**Methods:** We systematically and continually search PubMed, ACL Anthology, arXiv, OpenAlex via EPPI-Reviewer, and the *dblp computer science bibliography*. Full text screening and data extraction are conducted within an open-source living systematic review application created for the purpose of this review. This living review update includes publications up to December 2022 and OpenAlex content up to March 2023.

**Results: **76 publications are included in this review. Of these, 64 (84%) of the publications addressed extraction of data from abstracts, while 19 (25%) used full texts. A total of 71 (93%) publications developed classifiers for randomised controlled trials. Over 30 entities were extracted, with PICOs (population, intervention, comparator, outcome) being the most frequently extracted. Data are available from 25 (33%), and code from 30 (39%) publications. Six (8%) implemented publicly available tools

**Conclusions:** This living systematic review presents an overview of (semi)automated data-extraction literature of interest to different types of literature review. We identified a broad evidence base of publications describing data extraction for interventional reviews and a small number of publications extracting epidemiological or diagnostic accuracy data. Between review updates, trends for sharing data and code increased strongly: in the base-review, data and code were available for 13 and 19% respectively, these numbers increased to 78 and 87% within the 23 new publications. Compared with the base-review, we observed another research trend, away from straightforward data extraction and towards additionally extracting relations between entities or automatic text summarisation. With this living review we aim to review the literature continually.

## 1. Introduction

In a systematic review, data extraction is the process of capturing key characteristics of studies in structured and standardised form based on information in journal articles and reports. It is a necessary precursor to assessing the risk of bias in individual studies and synthesising their findings. Interventional, diagnostic, or prognostic systematic reviews routinely extract information from a specific set of fields that can be predefined. ^1^ The most common fields for extraction in interventional reviews are defined in the PICO framework (population, intervention, comparison, outcome) and similar frameworks are available for other review types. The data extraction task can be time-consuming and repetitive when done by hand. This creates opportunities for support through intelligent software, which identify and extract information automatically. When applied to the field of health research, this (semi) automation sits at the interface between evidence-based medicine (EBM) and data science, and as described in the following section, interest in its development has grown in parallel with interest in AI in other areas of computer science.

### 1.1 Related systematic reviews and overviews

This review is, to the best of our knowledge, the only living systematic review (LSR) of data extraction methods. We identified four previous reviews of tools and methods in the first iteration of this living review (called base-review hereafter), ^2^^–^^5^ and two documents providing overviews and guidelines relevant to our topic. ^3^^,^^6^^,^^7^ Between base-review and this update, we identified six more related (systematic) literature reviews that will be summarised in the following paragraphs. ^8^^–^^13^

**Related reviews up to 2014:** The systematic reviews from 2014 to 2015 present an overview of classical machine learning and natural language processing (NLP) methods applied to tasks such as data mining in the field of evidence-based medicine. At the time of publication of these documents, methods such as topic modelling (Latent Dirichlet Allocation) and support vector machines (SVM) were considered state-of-the art for language models.

In 2014, Tsafnat *et al.* provided a broad overview on automation technologies for different stages of authoring a systematic review. ^5^ O’Mara-Eves *et al*. published a systematic review focusing on text-mining approaches in 2015. ^4^ It includes a summary of methods for the evaluation of systems, such as recall, accuracy, and F1 score (the harmonic mean of recall and precision, a metric frequently used in machine-learning). The reviewers focused on tasks related to PICO classification and supporting the screening process. In the same year, Jonnalagadda, Goyal and Huffman ^3^ described methods for data extraction, focusing on PICOs and related fields. The age of these publications means that the latest static or contextual embedding-based and neural methods are not included. These newer methods, ^14^ however, are used in contemporary systematic review automation software which will be reviewed in the scope of this living review.

**Related reviews up to 2020:** Reviews up to 2020 focus on discussions around tool development and integration in practice, and mark the starting date of the inclusion of automation methods based on neural networks. Beller *et al.* describe principles for development and integration of tools for systematic review automation. ^6^ Marshall and Wallace ^7^ present a guide to automation technology, with a focus on availability of tools and adoption into practice. They conclude that tools facilitating screening are widely accessible and usable, while data extraction tools are still at piloting stages or require a higher amount of human input.

A systematic review of machine-learning for systematic review automation, published in Portuguese in 2020, included 35 publications. The authors examined journals in which publications about systematic review automation are published, and conducted a term-frequency and citation analysis. They categorised papers by systematic review task, and provided a brief overview of data extraction methods. ^2^

**Related reviews after 2020:** These six reviews include and discuss end-user tools and cover different tasks across the SR workflow, including data extraction. Compared with this LSR, these reviews are broader in scope but have less included references on the automation of data extraction. Ruiz and Duffy ^10^ did a literature and trend analysis showing that the number of published references about SR automation is steadily increasing. Sundaram and Berleant ^11^ analyse 29 references applying text mining to different parts of the SR process and note that 24 references describe automation in study selection while research gaps are most prominent for data extraction, monitoring, quality assessment, and synthesis. ^11^ Khalil et al. ^9^ include 47 tools and descriptions of validation studies in a scoping review, of which 8 are available end-user tools that mostly focus on screening, but also cover data extraction and risk of bias assessments. They discuss limitations of tools such as lack of generalisability, integration, funding, and limited performance or access. ^9^ Cierco Jimenez et al. ^8^ included 63 references in a mapping review of machine-learning to assist SRs during different workflow steps, of which 41 were available end-user tools for use by researchers without informatics background. In accordance with other reviews they describe screening as the most frequently automated step, while automated data extraction tools are lacking due to the complexity of the task. Zhang et al. ^12^ included 49 references on automation of data extraction fields such as diseases, outcomes, or metadata. They focussed on extraction from traditional Chinese medicine texts such as published clinical trial texts, health records, or ancient literature. ^12^ Schmidt et al. ^13^ published a narrative review of tools with a focus on living systematic review automation. They discuss tools that automate or support the constant literature retrieval that is the hallmark of LSRs, while well-integrated (semi) automation of data extraction and automatic dissemination or visualisation of results between official review updates is supported by some, but less common.

### 1.2 Aim

We aim to review published methods and tools aimed at automating or (semi) automating the process of data extraction in the context of a systematic review of medical research studies. We will do this in the form of a living systematic review, keeping information up to date and relevant to the challenges faced by systematic reviewers at any time.

Our objectives in reviewing this literature are two-fold. First, we want to examine the methods and tools from the data science perspective, seeking to reduce duplicate efforts, summarise current knowledge, and encourage comparability of published methods. Second, we seek to highlight the added value of the methods and tools from the perspective of systematic reviewers who wish to use (semi) automation for data extraction, i.e., what is the extent of automation? Is it reliable? We address these issues by summarising important caveats discussed in the literature, as well as factors that facilitate the adoption of tools in practice.

## 2. Methods

### 2.1 Registration/protocol

This review was conducted following a preregistered and published protocol. ^15^ PROSPERO was initially considered as platform for registration, but it is limited to reviews with health-related outcomes. Any deviations from the protocol have been described below.

### 2.2 Living review methodology

We are conducting a living review because the field of systematic review (semi) automation is evolving rapidly along with advances in language processing, machine-learning and deep-learning.

**Figure 1.**
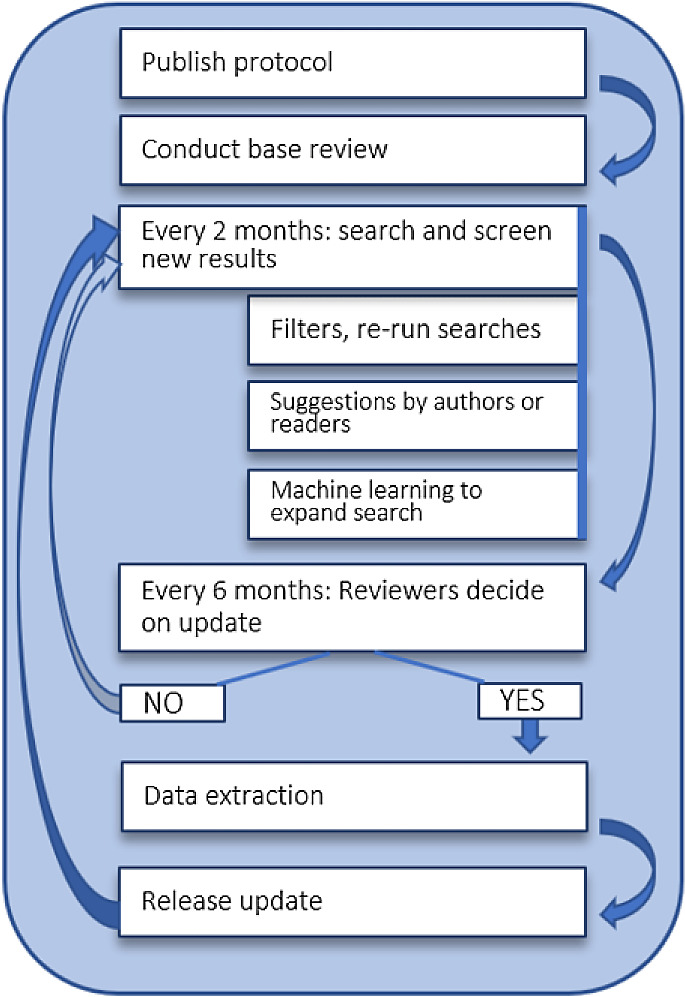
Continuous updating of the living review. This image is reproduced under the terms of a Creative Commons Attribution 4.0 International license (CC-BY 4.0) from Schmidt et al. ^15^

The process of updating started as described in the protocol ^15^ and is shown in Figure 1. In short, we will continuously update the literature search results, using the search strategies and methods described in the section ‘Search’ below. PubMed and arXiv search results are updated daily in a completely automated fashion via APIs. Articles from the dblp, ACL, and OpenAlex via EPPI-Reviewer are added every two months. All search results are automatically imported to our living review screening and data extraction web-application, which is described in the section ‘Data collection and analysis’ below.

The decision for full review updates is made every six months based on the number of new publications added to the review. For more details about this, please refer to the protocol or to the Cochrane living systematic review guidance. In between updates, the screening process and current state of the data extraction is visible via the living review website.

### 2.3 Eligibility criteria

• We included full text publications that describe an original NLP approach for extracting data related to systematic reviewing tasks. Data fields of interest (referred to here as entities or as sentences) were adapted from the Cochrane Handbook for Systematic Reviews of Interventions, ^1^ and are defined in the protocol. ^15^ We included the full range of NLP methods (e.g., regular expressions, rule-based systems, machine learning, and deep neural networks).
• Publications must describe a full cycle of the implementation and evaluation of a method. For example, they must report training and at least one measure of evaluating the performance of a data extraction algorithm.
• We included reports published from 2005 until the present day, similar to previous work. ^3^ We would have translated non-English reports, had we found any.
• The data that the included publications use for mining must be texts from randomised controlled trials, comparative cohort studies, case control studies or comparative cross-sectional studies (e.g., for diagnostic test accuracy). The scope of data extraction methods can be applied to the full text or to abstracts within each eligible publication’s corpus. We included publications that extracted data from other study types, as long as at least one of our study types of interest was contained in the corpus.

We excluded publications reporting:

• Methods and tools related solely to image processing and importing biomedical data from PDF files without any NLP approach, including data extraction from graphs.
• Any research that focuses exclusively on protocol preparation, synthesis of already extracted data, write-up, solely the pre-processing of text or its dissemination.
• Methods or tools that provided no natural language processing approach and offered only organisational interfaces, document management, databases, or version control
• Any publications related to electronic health reports or mining genetic data.

### 2.4 Search

**Base-review:** We searched five electronic databases, using the search methods previously described in our protocol. ^15^ In short, we searched MEDLINE via Ovid, using a search strategy developed with the help of an information specialist, and searched Web of Science Core Collection and IEEE using adaptations of this strategy, which were made by the review authors. Searches on the arXiv (computer science) and dblp were conducted on full database dumps using the search functionality described by McGuinness and Schmidt. ^16^ The full search results and further information about document retrieval are available in *Underlying data:* Appendix A and B. ^127^

Originally, we planned to include a full literature search from the Web of Science Core Collection. Due to the large number of publications retrieved via this search (n = 7822) we decided to first screen publications from all other sources, to train a machine-learning ensemble classifier, and to only add publications that were predicted as relevant for our living review. This reduced the Web of Science Core Collection publications to 547 abstracts, which were added to the studies in the initial screening step. The dataset, code and weights of trained models are available in *Underlying data:* Appendix C. ^127^ This includes plots of each model’s evaluation in terms of area under the curve (AUC), accuracy, F1, recall, and variance of cross-validation results for every metric.

Update: As planned, we changed to the PubMed API for searching MEDLINE. This decision was made to facilitate continuous reference retrieval. We searched only for pre-print or published literature and therefore did not search sources such as GITHUB or other source code repositories.

Update: We searched PubMed via its API, arXiv (computer science), ACL-Anthology, dblp, and used EPPI-Reviewer to collect citations from MicrosoftAcademic and later OpenAlex using the ‘Bi-Citation AND Recommendations’ method.

### 2.5 Data collection and analysis

***2.5.1 Selection of studies***

Initial screening and data extraction were conducted as stated in the protocol. In short, for the base-review we screened all retrieved publications using the Abstrackr tool. All abstracts were screened by two independent reviewers. Conflicting judgements were resolved by the authors who made the initial screening decisions. Full texts screening was conducted in a similar manner to abstract screening but used our web application for LSRs described in the following section.

For the updated review we used our living review web application to retrieve all publications with the exception of the items retrieved by EPPI-Reviewer (these are added to the dataset separately). We further used our application to de-duplicate, screen, and data-extract all publications.

A methodological update to the screening process included a change to single-screening to assess eligibility on both abstract and full-text level, reducing dual-screening to 10% of the publications.

***2.5.2 Data extraction, assessment, and management***

We previously developed a web application to automate reference retrieval for living review updates (see *Software availability* ^17^), to support both abstract and full text screening for review updates, and to manage the data extraction process throughout. ^17^ For future updates of this living review we will use the web application, and not Abstrackr, for screening references. This web application is already in use by another living review. ^18^ It automates daily reference retrieval from the included sources and has a screening and data extraction interface. All extracted data are stored in a database. Figures and tables can be exported on a daily basis and the progress in between review updates is shared on our living review website. The full spreadsheet of items extracted from each included reference is available in the *Underlying data.* ^127^ As previously described in the protocol, quality of reporting and reproducibility was initially assessed based on a previously published checklist for reproducibility in text mining, but some of the items were removed from the scope of this review update. ^19^

As planned in the protocol, a single reviewer conducted data extraction, and a random 10% of the included publications were checked by a second reviewer.

***2.5.3 Visualisation***

The creation of all figures and interactive plots on the living review website and in this review’s ‘Results’ section was automated based on structured content from our living review database (see Appendix A and D, *Underlying data* ^127^). We automated the export of PDF reports for each included publication. Calculation of percentages, export of extracted text, and creation of figures was also automated.

***2.5.4 Accessibility of data***

All data and code are free to access. A detailed list of sources is given in the ‘Data availability’ and ‘Software availability’ sections.

### 2.6 Changes from protocol and between updates

In the protocol we stated that data would be available via an OSF repository. Instead, the full review data are available via the Harvard Dataverse, as this repository allows us to keep an assigned DOI after updating the repository with new content for each iteration of this living review. We also stated that we would screen all publications from the Web of Science search. Instead, we describe a changed approach in the Methods section, under ‘Search’. For review updates, Web of Science was dropped and replaced with OpenAlex searches via EPPI-Reviewer.

We added a data extraction item for the type of information which a publication mines (e.g. P, IC, O) into the section of primary items of interest, and we moved the type of input and output format from primary to secondary items of interest. We grouped the secondary item of interest ‘Other reported metrics, such as impacts on systematic review processes (e.g., time saved during data extraction)’ with the primary item of interest ‘Reported performance metrics used for evaluation’.

The item ‘Persistence: is the dataset likely to be available for future use?’ was changed to: ‘Can data be retrieved based on the information given in the publication?’. We decided not to speculate if a dataset is likely to be available in the future and chose instead to record if the dataset was available at the time when we tried to access it.

The item ‘Can we obtain a runnable version of the software based on the information in the publication?’ was changed to ‘Is an app available that does the data mining, e.g. a web-app or desktop version?’.

In this current version of the review we did not yet contact the authors of the included publications. This decision was made due to time constraints, however reaching out to authors is planned as part of the first update to this living review.

In the base-review we assessed the included publications based on a list of 17 items in the domains of reproducibility (3.4.1), transparency (3.4.2), description of testing (3.4.3), data availability (3.4.4), and internal and external validity (3.4.5). The list of items was reduced to six items for the update:

• 3.4.2.2 Is there a description of the dataset used and of its characteristics?
• 3.4.2.4 Is the source code available?
• 3.4.3.2 Are basic metrics reported (true/false positives and negatives)?
• 3.4.4.1 Can we obtain a runnable version of the software based on the information in the publication?
• 3.4.4.2 Persistence: Can data be retrieved based on the information given in the publication?
• 3.4.5.1 Does the dataset or assessment measure provide a possibility to compare to other tools in the same domain?

The following items were removed, although the results and discussion from the assessment of these items in the base-review remains within the review text:

• 3.4.1.1 Are the sources for training/testing data reported?
• 3.4.1.2 If pre-processing techniques were applied to the data, are they described?
• 3.4.2.1 Is there a description of the algorithms used?
• 3.4.2.3 Is there a description of the hardware used?
• 3.4.3.1 Is there a justification/an explanation of the model assessment?
• 3.4.3.3 Does the assessment include any information about trade-offs between recall or precision (also known as sensitivity and positive predictive value)?
• 3.4.4.3 Is the use of third-party frameworks reported and are they accessible?
• 3.4.5.2 Are explanations for the influence of both visible and hidden variables in the dataset given?
• 3.4.5.3 Is the process of avoiding overfitting or underfitting described?
• 3.4.5.4 Is the process of splitting training from validation data described?
• 3.4.5.5 Is the model’s adaptability to different formats and/or environments beyond training and testing data described?

## 3. Results

### 3.1 Results of the search

**Figure 2.**
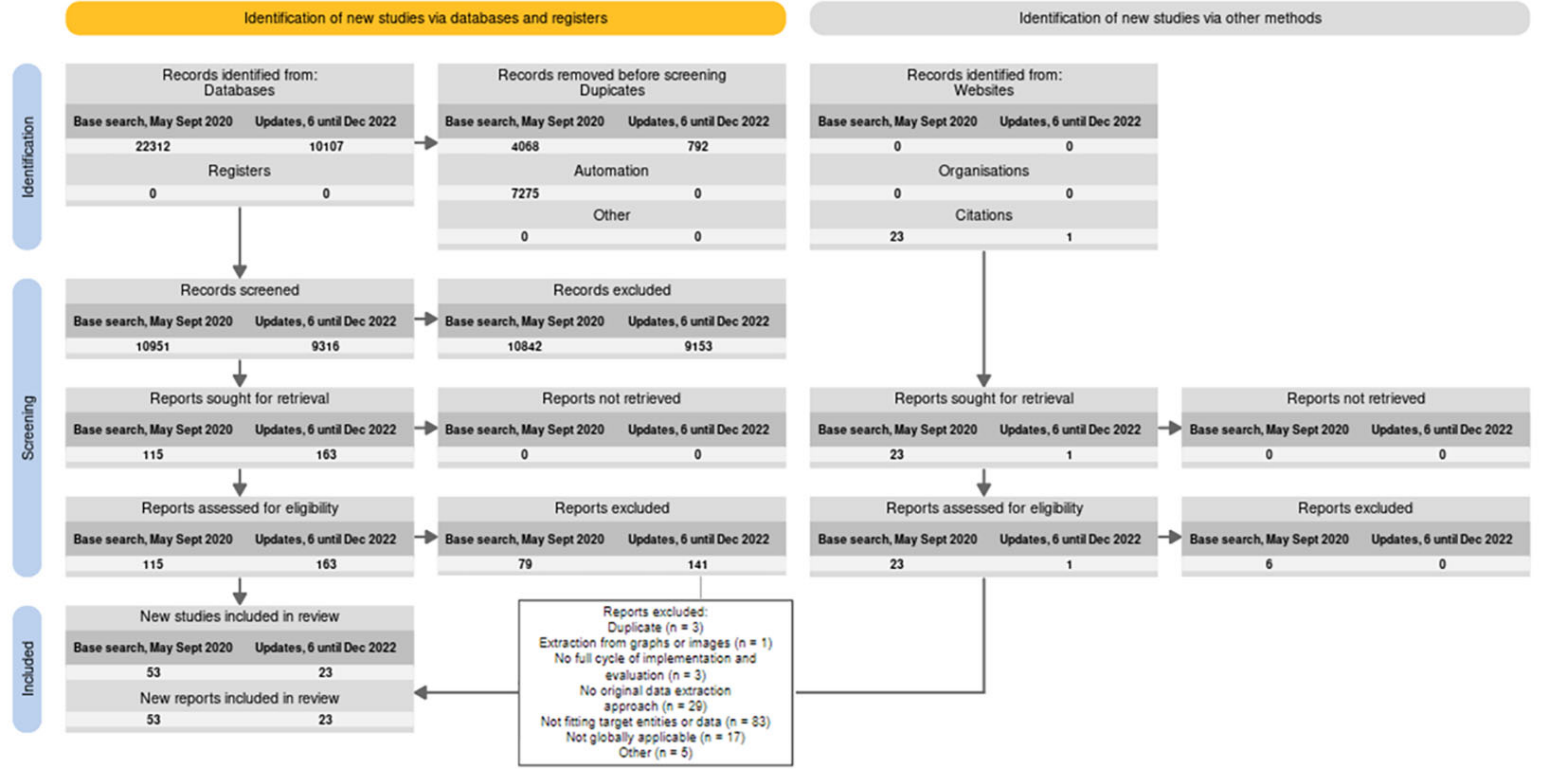
PRISMA2020 flow diagram adapted for living reviews. ^20^^–^^22^

Our database searches identified 10,107 publications after duplicates were removed (see Figure 2). We identified one more publication manually.

This iteration of the living review includes 76 publications, summarised in Table A1 in *Underlying data* ^127^).

***3.1.1 Excluded publications***

Across the base-review and the update, 216 publications were excluded at the full text screening stage, with the most common reason for exclusion being that it did not fit target entities or target data. In most cases, this was due to the text-types mined in the publications. Electronic health records and non-trial data were common, and we created a list of datasets that would be excluded in this category (see more information in *Underlying data:* Appendix B ^127^). Some publications addressed the right kind of text but were excluded for not mining data of interest to this review. For example, Norman, Leeflang and Névéol ^23^ performed data extraction for diagnostic test accuracy reviews, but focused on extracting the results and data for statistical analyses. Millard, Flach and Higgins ^24^ and Marshall, Kuiper and Wallace ^25^ looked at risk of bias classification, which is beyond the scope of this review. Boudin, Nie and Dawes ^26^ developed a weighing scheme based on an analysis of PICO element locations, leaving the detection of single PICO elements for future work. Luo *et al*. ^27^ extracted data from clinical trial registrations but focused on parsing inclusion criteria into event or temporal entities to aid participant selection for randomised controlled trials (RCTs).

The second most common reason for study exclusion was that they had ‘no original data extraction approach’. Rathbone *et al*., ^28^ for example, used hand-crafted Boolean searches specific to a systematic review’s PICO criteria to support the screening process of a review within Endnote. We classified this article as not having any original data extraction approach because it does not create any structured outputs specific to P, IC, or O. Malheiros *et al.* ^29^ performed visual text mining, supporting systematic review authors by document clustering and text highlighting. Similarly, Fabbri *et al.* ^30^ implemented a tool that supports the whole systematic review workflow, from protocol to data extraction, performing clustering and identification of similar publications. Other systematic reviewing tasks that can benefit from automation but were excluded from this review are listed in *Underlying data:* Appendix B. ^127^

### 3.2 Results from the data extraction: Primary items of interest

***3.2.1 Automation approaches used***

**Figure 3.**
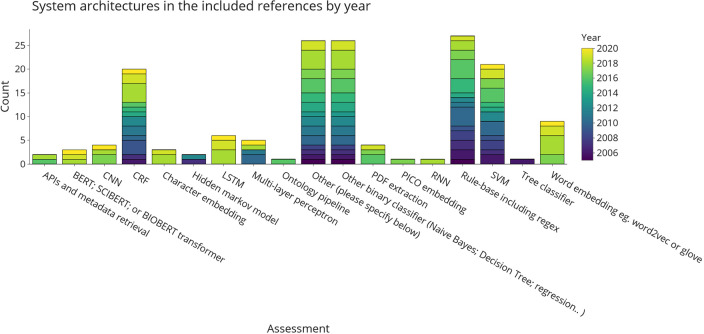
System architectures used for automating data extraction in the included publications. Results are divided into different categories of machine-learning and natural language processing approaches and coloured by the year of publication. More than one architecture component per publication is possible. Where API, application programming interface; BERT, bidirectional encoder representations from Transformers; CNN, convolutional neural network; CRF, conditional random fields; LSTM, long short-term memory; PICO, population, intervention, comparison, outcome; RNN, recurrent neural networks; SVM, support vector machines.

Figure 3 shows aspects of the system architectures implemented in the included publications. A short summary of these for each publication is provided in Table A1 in *Underlying data.* ^127^ Where possible, we tried to break down larger system architectures into smaller components. For example, an architecture combining a word embedding + long short-term memory (LSTM) network would have been broken down into the two respective sub-components. We grouped binary classifiers, such as naïve Bayes and logistic regression. Although SVM is also binary classifier, it was assigned as separate category due to its popularity. The final categories are a mixture of non-machine-leaning automation (application programming interface (API) and metadata retrieval, PDF extraction, rule-base), classic machine-learning (naïve Bayes, decision trees, SVM, or other binary classifiers) and neural or deep-learning approaches (convolutional neural network (CNN), LSTM, transformers, or word embeddings). This figure shows that there is no obvious choice of system architecture for this task. For the LSR update, the strongest trend was the increasing application of BERT (Bidirectional Encoder Representations from Transformers). BERT was published in 2018 and other architecturally-identical versions of it tailored to using scientific text, such as SciBERT, are summarised under the same category in this review. ^14^^,^^31^ In the base-review, BERT was used three times, whilst now appearing 21 times. Other transformer-based architectures such as the bio-pretrained version of ELECTRA, are also gaining attention, ^32^^,^^33^ as well as FLAIR-based models. ^34^^–^^36^

Rule-bases, including approaches using heuristics, wordlists, and regular expressions, were one of the earliest techniques used for data extraction in EBM literature. It remains the most frequently used approaches to automation. Nine publications (12%) use rule-bases alone, while the rest of the publications use them in combination with other classifiers (data shown in *Underlying data:* Appendix A and D ^127^). Although used more frequently in the past, the 11 publications published between 2017 and now that use this approach alongside other architectures such as BERT, ^33^^,^^37^^–^^39^ conditional random fields (CRF), ^40^ use it with SVM ^41^ or other binary classifiers. ^42^ In practice, these systems use rule-bases in the form of hand-crafted lists to identify candidate phrases for amount entities such as sample size ^42^^,^^43^ or to refine a result obtained by a machine-learning classifier on the entity level (e.g., instances where a specific intervention or outcome is extracted from a sentence). ^40^

Binary classifiers, most notably naïve Bayes and SVMs, are also frequently used system components in the data extraction literature. They are frequently used in studies published between 2005 and now but their usage started declining with the advent of neural models.

Embedding and neural architectures are increasingly being used in literature over the past seven years. Recurrent neural networks (RNN), CNN, and LSTM networks require larger amounts of training data; by using transformer-based embeddings with pre-training algorithms based on unlabelled data they have become increasingly more interesting in fields such as data extraction for EBM- where high-quality training data are difficult and expensive to obtain.

In the ‘Other’ category, tools mentioned were mostly other classifiers such as maximum entropy classifiers (n = 3), kLog, J48, and various position or document-length classification algorithms. We also added methods such as supervised distant supervision (n = 3, see Ref. 44) and novel training approaches to existing neural architectures in this category.

***3.2.2 Reported performance metrics used for evaluation***

**Figure 4.**
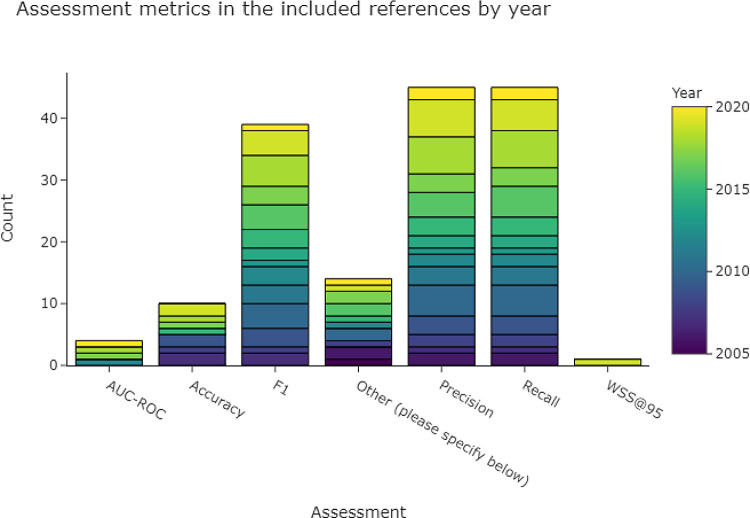
The most common assessment metrics used in the included publications in order to evaluate the performance of a data extraction system. More than one metric per publication is possible, which means that the total number of included publications (n = 76) is lower than the sum of counts of the bars within this figure. AUC-ROC, area under the curve - receiver operator characteristics; F1, harmonic mean of precision and recall.

Precision (i.e., positive predictive value), recall (i.e., sensitivity), and F1 score (harmonic mean of precision and recall) are the most widely used metrics for evaluating classifiers. This is reflected in Figure 4, which shows that at least one of these metrics was used in the majority of the included publications. Accuracy and area under the curve - receiver operator characteristics (AUC-ROC) were less frequently used.

There were several approaches and justifications of using macro- or micro-averaged precision, recall, or F1 scores in the included publications. Micro or macro scores are computed in multi-class cases, and the final scores can differ whenever the classes in a dataset are imbalanced (as is the case in most datasets used for automating data extraction in SR automation).

Both micro and macro scores were reported by Singh et al. (2021), ^45^ Kilicoglu et al. (2021), ^38^ Kiritchenko et al. (2010), ^46^ Fiszman et al. (2007) ^47^ whereas Karystianis et al. (2014, 2017) ^48^^,^^49^ reported micro across documents, and macro across the classes.

Macro-scores were used in one publication. ^37^

Micro scores were used by Fiszman et al. ^47^ for class-level results. In one publication harmonic mean was used for precision and recall, while micro-scoring was used for F1. ^50^ Micro scores were most widely used, including Al-Hussaini et al. (2022), ^32^ Sanchez-Graillet et al. (2022), ^51^ Kim et al. (2011), ^52^ Verbeke et al. (2012), ^53^ and Jin and Szolovits (2020) ^54^ were used in the evaluation script of Nye et al. (2018). ^55^

In the category ‘Other’ we added several instances where a relaxation of a metric was introduced, e.g., precision using top-n classified sentences ^44^^,^^46^^,^^56^ or mean average precision and the metric ‘precision @rank 10’ for sentence ranking exercises. ^57^^,^^58^ Another type of relaxation for standard metrics is a distance relaxation when normalising entities into concepts in medical subject headings (MesH) or unified medical language system (UMLS), to allow N hops between predicted and target concepts. ^59^

The LSR update showed an increasing trend of text summarisation and relation extraction algorithms. ROGUE, ∆EI, or Jaccard similarity were metrics for summarisation. ^60^^,^^61^ For relation extraction F1, precision, and recall remained the most common metrics. ^62^^,^^63^

Other metrics were kappa, ^58^ random shuffling ^64^ or binomial proportion test ^65^ to test statistical significance, given with confidence intervals. ^41^ Further metrics included under ‘Other’ were odds ratios, ^66^ normalised discounted cumulative gain, ^44^^,^^67^ ‘sentences needed to screen per article’ in order to find one relevant sentence, ^68^ McNemar test, ^65^ C-statistic (with 95% CI) and Brier score (with 95% CI). ^69^ Barnett (2022) ^70^ extracted sample sizes and reported the mean difference between true and extracted numbers.

Real-life evaluations, such as the percentage of outputs needing human correction, or time saved per article, were reported by two publications, ^32^^,^^46^ and an evaluation as part of a wider screening system was done in another. ^71^

***3.2.3 Type of data***

3.2.3.1 Scope and data

Most data extraction is carried out on abstracts (See Table A1 in *Underlying data*, ^127^ and the supplementary table giving an overview of all included publications). Abstracts are the most practical choice, due to the possibility of exporting them along with literature search results from databases such as MEDLINE. In total, 84% (N=64) of the included publications directly reported using abstracts. Within the 19 references (25%) that reported usage of full texts, eight specifically mentioned that this also included abstracts but it is unclear if all full texts included abstract text. Descriptions of the benefits of using full texts for data extraction include having access to a more complete dataset, while the benefits of using titles (N=4, 5%) include lower complexity for the data extraction task. ^43^ Xu et al. (2010) ^72^ exclusively used titles, while the other three publications that specifically mentioned titles also used abstracts in their datasets. ^43^^,^^73^^,^^74^

**Figure 5.**
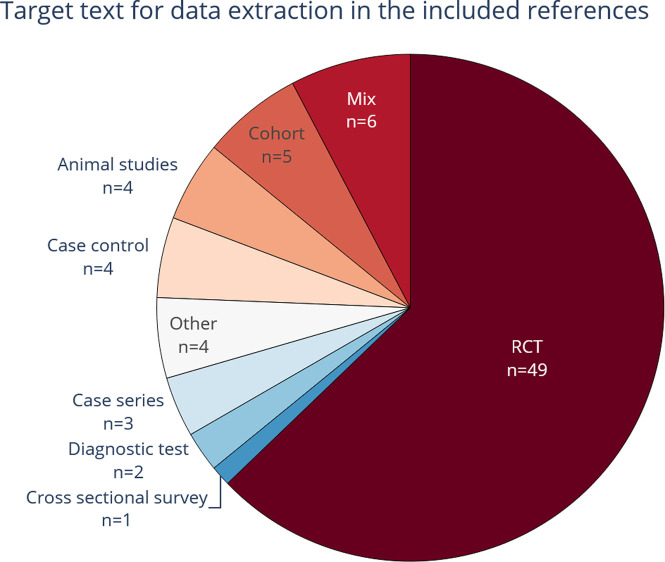
The study types from which data were extracted. Commonly, randomized controlled trials (RCT) text was at least one of the target text types used in the included publications.

Figure 5 shows that RCTs are the most common study design texts used for data extraction in the included publications (see also extended Table A1 in *Underlying data* ^127^). This is not surprising, because systematic reviews of interventions are the most common type of systematic review, and they are usually focusing on evidence from RCTs. Therefore, the literature for automation of data extraction focuses on RCTs, and their related PICO elements. Systematic reviews of diagnostic test accuracy are less frequent, and only one included publication specifically focused on text and entities related to these studies, ^75^ while two mentioned diagnostic procedures among other fields of interest. ^35^^,^^76^ Eight publications focused on extracting data specifically from epidemiology research, non-randomised interventional studies, or included text from cohort studies as well as RCT text. ^48^^,^^49^^,^^61^^,^^72^^–^^74^^,^^76^^,^^77^ More publications mining data from surveys, animal RCTs, or case series might have been found if our search and review had concentrated on these types of texts.

3.2.3.2 Data extraction targets

**Figure 6.**
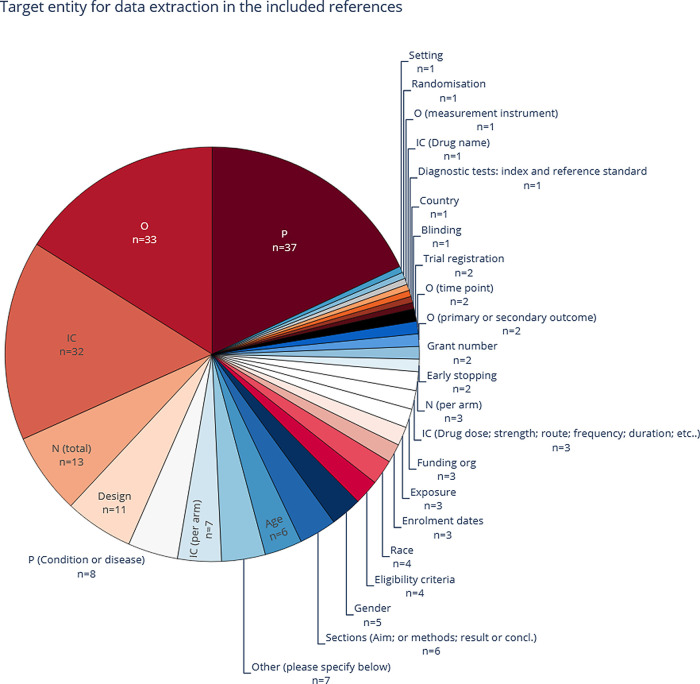
The most common entities, as extracted in the included publications. More than one entity type per publication is common, which means that the total number of included publications (n = 76) is lower than the sum of counts within this figure. P, population; I, intervention; C, comparison; O, outcome.

Mining P, IC, and O elements is the most common task performed in the literature of systematic review (semi-) automation (see Table A1 in *Underlying data*, ^127^ and Figure 6). In the base-review, P was the most common entity. After the LSR update, O (n=52, 68%) has become the most popular, due to the emerging trend of relation-extraction models that focus on the relationship between O and I entities and therefore may omit the automatic extraction of P. Some of the less-frequent data extraction targets in the literature can be categorised as sub-classes of a PICO, ^55^ for example, by annotating hierarchically multiple entity types such as health condition, age, and gender under the P class. The entity type ‘P (Condition and disease)’, was the most common entity closely related to the P class, appearing in twelve included publications, of which four were published in 2021 or later. ^35^^,^^36^^,^^51^^,^^55^^,^^63^^,^^71^^,^^75^^,^^76^^,^^78^^–^^81^

Notably, eleven publications annotated or worked with datasets that differentiated between intervention and control arms, four of these published after 2020 with a trend towards relation extraction and summarisation tasks requiring this type of data. ^46^^,^^47^^,^^51^^,^^56^^,^^62^^,^^63^^,^^66^^,^^82^^–^^84^ Usually, I and C are merged (n=47). Most data extraction approaches focused on recognising instances of entity or sentence classes, and a small number of publications went one step further to normalise to actual concepts and including data sources such as UMLS (Unified Medical Language System). ^35^^,^^39^^,^^59^^,^^73^^,^^85^

The ‘Other’ category includes some more detailed drug annotations ^65^ or information such as confounders ^49^ and other entity types (see the full dataset in *Underlying data:* Appendix A and D for more information ^127^).

### 3.3 Results from the data extraction: Secondary items of interest

***3.3.1 Granularity of data extraction***

A total of 54 publications (71%) extracted at least one type of information at the entity level, while 46 publications (60%) used sentence level (see Table A1 extended version in *Underlying data* ^127^). We defined the entity level as any number of words that is shorter than a whole sentence, e.g., noun-phrases or other chunked text. Data types such as P, IC, or O commonly appeared to be extracted on both entity and sentence level, whereas ‘N’, the number of people participating in a study, was commonly extracted on entity level only.

***3.3.2 Type of input***

The majority of publications and benchmark corpora mentioned MEDLINE, via PubMed, as the data source for text. Text files (n = 64), next to XML (n = 8), or HTML (n = 3), are the most common format of the data downloaded from these sources. Therefore, most systems described using, or were assumed to use, text files as input data. Eight included publications described using PDF files as input. ^44^^,^^46^^,^^59^^,^^68^^,^^75^^,^^81^^,^^86^^,^^87^

***3.3.3 Type of output***

A limited number of publications described structured summaries as output of their extracted data (n = 14, increasing trend between LSR updates). Alternatives to exporting structured summaries were JSON (n = 4), XML, and HTML (n = 2 each). Two publications mentioned structured data outputs in the form of an ontology. ^51^^,^^88^ Most publications mentioned only classification scores without specifying an output type. In these cases, we assumed that the output would be saved as text files, for example as entity span annotations or lists of sentences (n = 55).

### 3.4 Assessment of the quality of reporting

In the base-review we used a list of 17 items to investigate reproducibility, transparency, description of testing, data availability, and internal and external validity of the approaches in each publication. The maximum and minimum number of items that were positively rated were 16 and 1, respectively, with a median of 10 (see Table A1 in *Underlying data* ^127^). Scores were added up and calculated based on the data provided in Appendix A and D (see *Underlying data* ^127^), using the sum and median functions integrated in Excel. Publications from recent years up to 2021 showed a trend towards more complete and clear reporting.

***3.4.1 Reproducibility***

3.4.1.1 Are the sources for training/testing data reported?

Of the included publications in the base-review, 50 out of 53 (94%) clearly stated the sources of their data used for training and evaluation. MEDLINE was the most popular source of data, with abstracts usually described as being retrieved via searches on PubMed, or full texts from PubMed Central. A small number of publications described using text from specific journals such as PLoS Clinical Trials, New England Journal of Medicine, The Lancet, or BMJ. ^56^^,^^83^ Texts and metadata from Cochrane, either provided in full or retrieved via PubMed, were used in five publications. ^57^^,^^59^^,^^68^^,^^75^^,^^86^ Corpora such as the ebm-nlp dataset, ^55^ or PubMed-PICO ^54^ are available for direct download. Publications published in recent years are increasingly reporting that they are using these benchmark datasets rather than creating and annotating their own corpora (see 4 for more details).

3.4.1.2 If pre-processing techniques were applied to the data, are they described?

Of the included publications in the base-review, 47 out of 53 (89%) reported processing the textual data before applying/training algorithms for data extraction. Different types of pre-processing, with representative examples for usage and implementation, are listed in Table 1 below.

**Table 1.** Pre-processing techniques, a short description and examples from the literature.

Technique	Details	Example in literature
Tokenisation	Splitting text on sentence and word level	^56^^,^^83^^,^^88^
Normalisation	Replacing integers, units, dates, lower-casing	^65^^,^^89^^,^^90^
Lemmatisation and stemming	Reducing words to shorter or more common forms	^53^^,^^91^^,^^92^
Stop-word removal	Removing common words, such as ‘the’, from the text	^44^^,^^48^^,^^80^
Part-of-speech tagging and dependency parsing	Tagging words with their respective grammatical roles	^41^^,^^78^^,^^88^
Chunking	Defining sentence parts, such as noun-phrases	^65^^,^^76^^,^^93^
Concept tagging	Processing and tagging words with semantic classes or concepts, e.g. using word lists or MetaMap	^75^^,^^79^^,^^94^

After the publication of the base-review, transformer models such as BERT became dominant in the literature (see Figure 3). With their word-piece vocabulary, contextual embeddings, and self-supervised pre-training on large unlabelled corpora these models have essentially removed the need for most pre-processing beyond automatically-applied lower-casing. ^14^^,^^31^ We are therefore not going to update this table in this, or any future iterations of this LSR. We leave it for reference to publications that may still use these methods in the future.

***3.4.2 Transparency of methods***

3.4.2.1 Is there a description of the algorithms used?

**Figure 7.**
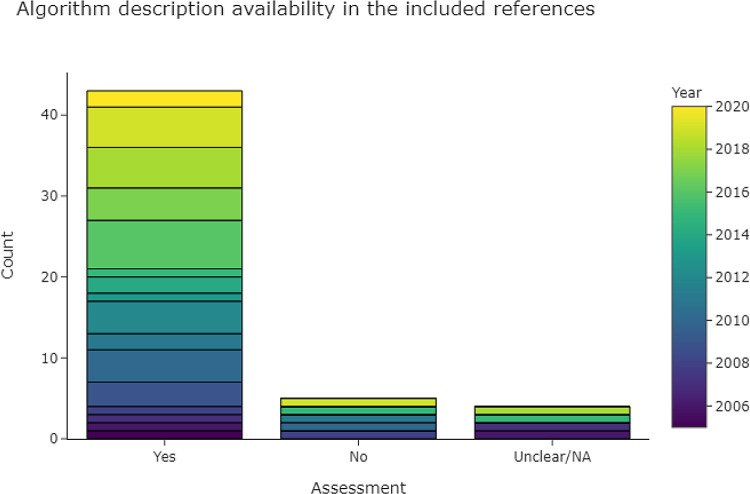
Bar chart, showing the levels of algorithm description in the included publications.

Figure 7 shows that 43 out of 53 publications in the base-review (81%) provided descriptions of their data extraction algorithm. In the case of machine learning and neural networks, we looked for a description of hyperparameters and feature generation, and for the details of implementation (e.g. the machine-learning framework). Hyperparameters were rarely described in full, but if the framework (e.g., Scikit-learn, Mallet, or Weka) was given, in addition to a description of implementation and important parameters for each classifier, then we rated the algorithm as fully described. For rule-based methods we looked for a description of how rules were derived, and for a list of full or representative rules given as examples. Where multiple data extraction approaches were described, we gave a positive rating if the best-performing approach was described.

3.4.2.2 Is there a description of the dataset used and of its characteristics?

Of the included publications in the review update, 73 out of 76 (97%) provided descriptions of their dataset and its characteristics.

Most publications provided descriptions of the dataset(s) used for training and evaluation. The size of each dataset, as well as the frequencies of classes within the data, were transparent and described for most included publications. All dataset citations, along with a short description and availability of the data, are shown in Table 4.

3.4.2.3 Is there a description of the hardware used?

Most included publications in the base-review did not report their hardware specifications, though five publications (9%) did. One, for example, applied their system to new, unlabelled data and reported that classifying the whole of PubMed takes around 20 hours using a graphics processing unit (GPU). ^69^ In another example, the authors reported using Google Colab GPUs, along with estimates of computing time for different training settings. ^95^

3.4.2.4 Is the source code available?

**Figure 8.**
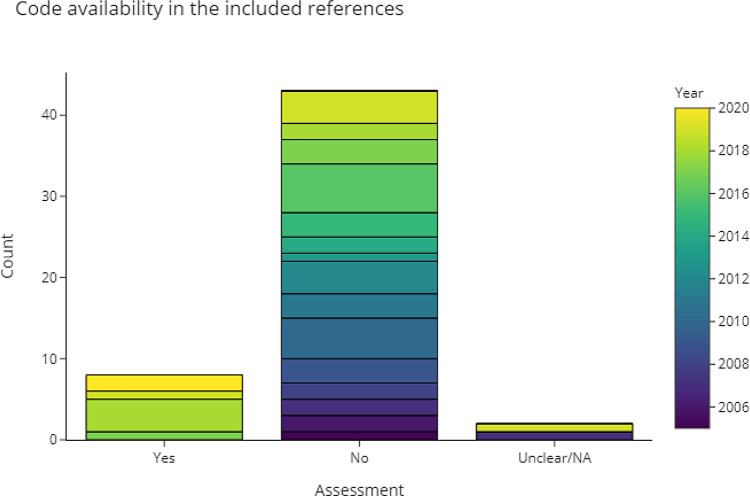
This chart shows the extent to which included publications provided access to their source code.

Figure 8 shows that most of the included publications did not provide any source code, although there is a very strong trend towards better code-availabilty in the publications from the review update (n=19 published code, 83% of the new publications provided code). Publications that did provide the source code were exclusively published or last updated in the last seven years. GitHub is the most popular platform for making code accessible. Some publications also provided links to notebooks on Google Colab, which is a cloud-based platform to develop and execute code online. Two publications provided access to parts of the code, or access was restricted. A full list of code repositories from the included publications is available in Table 2.

**Table 2.** Repositories containing source code for the included publications.

Publication	Code	LSR
^81^	Available under: https://github.com/ijmarshall/robotreviewer, older version: https://figshare.com/articles/Spa/997707	Base
^96^	Available under: https://github.com/jind11/LSTM-PICO-Detection	Base
^55^	Available under: https://github.com/bepnye/EBM-NLP https://colab.research.google.com/drive/1Ir52OmkJ2C_Iy9V_eS-_KFVLircJ4MXp https://colab.research.google.com/drive/1YbbQojM147Ybt1nEcyoXTqlvefmwMg-q	Base
^54^	Available under: https://github.com/jind11/Deep-PICO-Detection	Base
^97^	Available under: https://ii.nlm.nih.gov/DataSets/index.shtml	Base
^85^	Available under: https://github.com/Tian312/PICO_Parser	Base
^95^	Available under: https://github.com/L-ENA/HealthINF2020 https://www.kaggle.com/lenaschmidt0493/qa-integrated-biomedical-ner-classifier-for-pico	Base
^69^	Available under: https://github.com/ijmarshall/trialstreamer	Base
^47^	Unclear if Java code is accessible, pending user access: https://semrep.nlm.nih.gov/SemRep.v1.8_Installation.html#Download	Base
^75^	Used public Google implementation of transformers + https://zenodo.org/record/1303259#.X4wSoaySk2w	Base
^60^	Available under: https://github.com/smileslab/Brain_Aneurysm_Research/tree/master/BioMed_Summarizer	Update
^74^	Available under: https://github.com/nstylia/pico_entities/	Update
^98^	Available under: https://github.com/wds-seu/Aceso	Update
^62^	Available under: https://github.com/jayded/evidence-inference	Update
^61^	Available under: https://github.com/allenai/ms2	Update
^99^	Available under: https://github.com/Tian312/MD-Attention	Update
^38^	Available under: https://github.com/kilicogluh/CONSORT-TM	Update
^35^	Available under: https://github.com/lcampillos/Medical-NER	Update
^36^	Available under: https://gitlab.com/tomaye/ecai2020-transformer_based_am	Update
^50^	Available under: https://github.com/jetsunwhitton/RCT-ART	Update
^34^	Available under: https://github.com/LivNLP/ODP-tagger	Update
^33^	Available under: https://data.mendeley.com/datasets/ccfnn3jb2x/1	Update
^82^	Available under: https://osf.io/2dqcg/	Update
^51^	Available under: https://zenodo.org/record/6365890	Update
^45^	Available under: https://github.com/gauravsc/pico-tagging	Update
^67^	Available under: https://github.com/MichealAbaho/Label-Context-Aware-Attention-Model	Update
^100^	Available under: https://github.com/evidence-surveillance/sent2span	Update
^70^	Available under: https://zenodo.org/record/6647853#.ZBnpLXbP2Uk	Update
^37^	Available under: https://github.com/anjani-dhrangadhariya/distant-PICO	Update

***3.4.3 Testing***

3.4.3.1 Is there a justification/an explanation of the model assessment?

Of the included publications in the base-review, 47 out of 53 (89%) gave a detailed assessment of their data extraction algorithms. We rated this item as negative if only the performance scores were given, i.e., if no error analysis was performed and no explanations or examples were given to illustrate model performance. In most publications a brief error analysis was common, for example discussions on representative examples for false negatives and false positives, ^47^ major error sources ^90^ or highlighting errors with respect to every entity class. ^76^ Both Refs. 52, 53 used structured and unstructured abstracts, and therefore discussed the implications of unstructured text data for classification scores.

A small number of publications did a real-life assessment, where the data extraction algorithm was applied to different, unlabelled, and often much larger datasets or tested while conducting actual systematic reviews. ^46^^,^^58^^,^^63^^,^^69^^,^^48^^,^^95^^,^^101^^,^^102^

3.4.3.2 Are basic metrics reported (true/false positives and negatives)?

**Figure 9.**
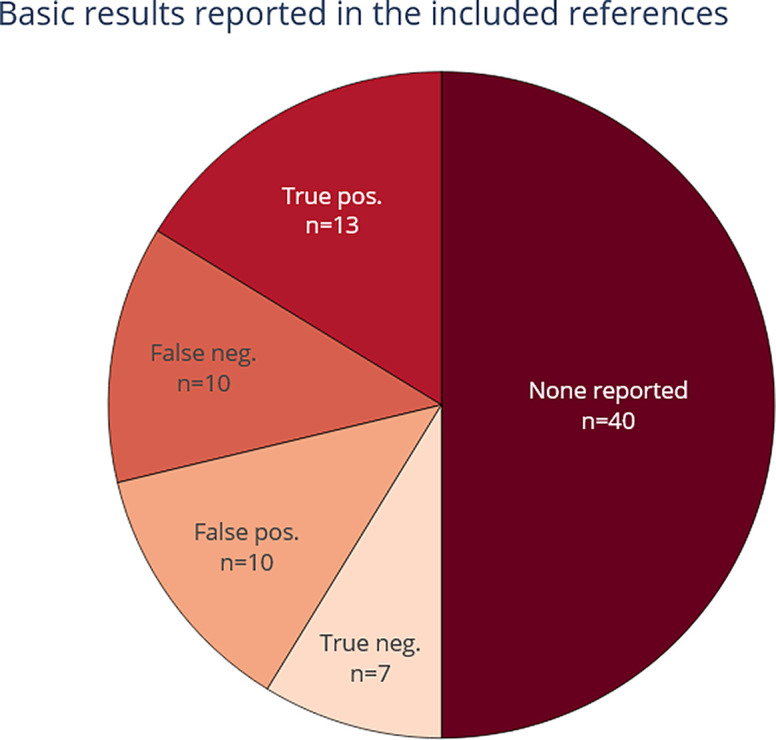
Reporting of basic metrics (true positive, false positive, true negative, and false negative). For each included paper. More than one selection is possible, which means that the total number of included publications (n=76) is lower than the sum of counts within this figure.

Figure 9 shows the extent to which all raw basic metrics, such as true-positives, were reported in the included publications in the LSR update. In most publications (n = 62) these basic metrics are not reported, and there is a trend between base-review and this update towards not reporting these. However, basic metrics could be obtained since the majority of new included publications made source code available and used publicly available datasets. When dealing with entity-level data extraction it can be challenging to define the quantity of true negative entities. This is true especially if entities are labelled and extracted based on text chunks, because there can be many combinations of phrases and tokens that constitute an entity. ^47^ This problem was solved in more recent publications by conducting a token-based evaluation that computes scores across every single token, hence gaining the ability to score partial matches for multi-word entities. ^55^

3.4.3.3 Does the assessment include any information about trade-offs between recall or precision (also known as sensitivity and positive predictive value)?

Of the included publications in the base-review, 17 out of 53 (32%) described trade-offs or provided plots or tables showing the development of evaluation scores if certain parameters were altered or relaxed. Recall (i.e., sensitivity) is often described as the most important metric for systematic review automation tasks, as it is a methodological demand that systematic reviews do not exclude any eligible data.

References 56 and 76 showed how the decision of extracting the top two or N predictions impacts the evaluation scores, for example precision or recall. Reference 102 shows precision-recall plots for different classification thresholds. Reference 72 shows four cut-offs, whereas Ref. 95 shows different probability thresholds for their classifier, and describe the impacts of this on precision, recall, and F1 curves.

Some machine-learning architectures need to convert text into features before performing classification. A feature can be, for example, the number of times that a certain word occurs, or the length of an abstract. The number of features used, e.g. for CRF algorithms, which was given in multiple publications, ^92^ together with a discussion of classifiers that should be used in high recall is needed. ^42^^,^^103^ show ROC curves quantifying the amount of training data and its impact on the scores.

***3.4.4 Availability of the final model or tool***

3.4.4.1 Can we obtain a runnable version of the software based on the information in the publication?

**Figure 10.**
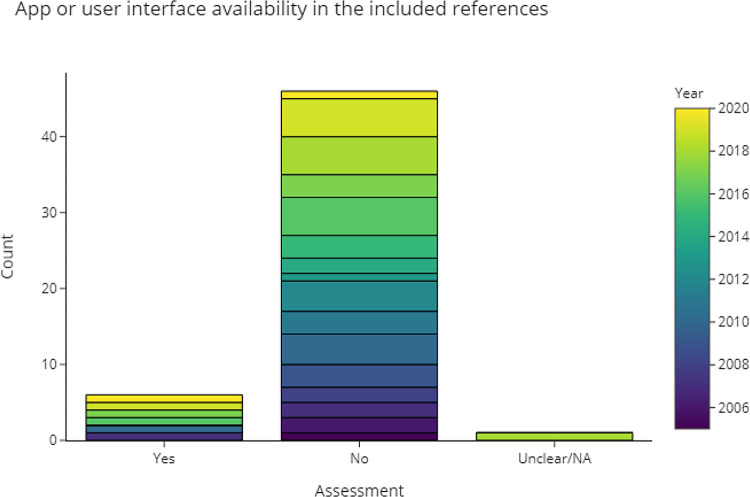
Publications that provide applications with user interface.

Compiling and testing code from every publication is outside the scope of this review. Instead, in Figure 10 and Table 3 we recorded the publications where a (web) interface or finished application was available. Counting RobotReviewer and Trialstreamer as separate projects, 12% of the included publications had an application associated with it, but only 5 (6%) are available and directly usable via web-apps. Applications were available as open-source, completely free, or free basic versions with optional features that can be purchased or subscribed to.

**Table 3.** Publications that provide user interfaces to their final data extraction system.

Paper	Access
^42^	Unclear: A link was given, but tool is not yet online: https://ihealth.uemc.es/
^43^	https://www.tripdatabase.com/#pico
^44, 81^	https://www.robotreviewer.net/
^46^	https://exact.cluster.gctools.nrc.ca/ExactDemo/
^47^	https://semrep.nlm.nih.gov/SemRep.v1.8_Installation.html, SemMed is a web-based application published after this publication was released: https://skr3.nlm.nih.gov/SemMed/semmed.html
^69^	Database with all extracted data is available online: https://trialstreamer.robotreviewer.net/
^58^	Pending: article mentions that an app is being implemented.
^36^	http://ns.inria.fr/acta/
^82^	App code for own deployment available here: https://osf.io/2dqcg/

3.4.4.2 Persistence: Can data be retrieved based on the information given in the publication?

We observed an increasing trend of dataset availability and publications re-using benchmark corpora within the LSR update. Only seven of the included publications in the base-review (13%) made their datasets publicly available, out of the 36 unique corpora found then.

After the LSR update we accumulated 55 publications that describe unique new corpora. Of these, 23 corpora were available online and a total of 40 publication mentioned using one of these public benchmarking sets. Table 4 shows a summary of the corpora, their size, classes, links to the datasets, and cross-reference to known publications re-using each data set. For the base review, we collected the corpora, provide a central link to all datasets, and will add datasets as they become available during the life span of this living review (see *Underlying data* ^127^^,^^128^ below). Due to the increased number of available corpora we stopped downloading the data and provide links instead. When a dataset is made freely available without barriers (i.e., direct downloads of text and labels), then any researcher can re-use the data and publish results from different models, which become comparable to one another. Copyright issues surrounding data sharing were noted by Ref. 75, therefore they shared the gold-standard annotations used as training or evaluation data and information on how to obtain the texts.

**Table 4.** Corpora used in the included publications. RCT, randomized controlled trials; IR, information retrieval; PICO, population, intervention, comparison, outcome; UMLS, unified medical language system.

Publication	Also used by	Description	Classes	Size/type	Availability	Note
^96^	^39^^,^^54^^,^^87^^,^^95^^,^^98^ Dataset adaptations: ^60^	Automatically labelled sentence labels from structured abstracts up to Aug’17	P, IC, O, Method	24,668 abstracts	Yes, https://github.com/jind11/PubMed-PICO-Detection	
^55^	^32^^,^^33^^,^^36^^,^^61^^,^^74^^,^^85^^,^^95^^,^^98^^,^^100^^,^^106^ Dataset adaptions: ^34^^,^^37^^,^^50^^,^^67^	Entities	P, IC, O + age, gender, and more entities	5,000 abstracts	Yes, https://github.com/bepnye/EBM-NLP	
^97^		Entities	I and dosage-related	694 abstract/full text	Yes, https://ii.nlm.nih.gov/DataSets/index.shtml	Domain drug-based interventions
^48^		Entities	P, O, Design, Exposure	60 + 30 abstracts	Yes, http://gnteam.cs.manchester.ac.uk/old/epidemiology/data.html	Domain obesity
^75^		Sentence level 90,000 distant supervision annotations, 1000 manual.	Target condition, index test and reference standard	90,000 + 1000 sentences	Yes (labels, not text), https://zenodo.org/record/1303259	Domain diagnostic tests
^52^	^64^ (includes classifiers from), ^40^^,^^53^^,^^54^^,^^102^^,^^107^^–^^110^	Structured and unstructured abstracts, multi-label on sentences.	P, IC, O, Design	1000 abstracts	Yes, https://drive.google.com/file/d/1M9QCgrRjERZnD9LM2FeK-3jjvXJbjRTl/view?usp=sharing	Multi-label sentences
^47^		Sentences	Drug intervention and comparative statements for each arm	300 (500 in available data) sentences	Yes, https://dataverse.harvard.edu/file.xhtml?fileId=4171005&version=1.0	Domain drug-based interventions
^98^		Sentences	P, IC, O	5099 sentences from references included in SRs, labelled using active-learning	Yes, https://github.com/wds-seu/Aceso/tree/master/datasets	Domain heart disease
^62^based on ^111^	^32^^,^^61^^,^^99^	Sentences	P, I, O	Fulltext: 12,616 prompts stemming from 3,346 articles; Abstract-only: 6375 prompts	Yes, http://evidence-inference.ebm-nlp.com/download/	Triplets for relation extraction
^61^		Sentences, Entities	P, IC, O	470 studies from 20k reviews, entity labels initially assigned via model trained on EBM-NLP	Yes, https://github.com/allenai/ms2	Relation extraction with direction of effect labels
^35^		Entities	P, IC, diagnostic test	500 abstracts and 700 trial records	Yes, http://www.lllf.uam.es/ESP/nlpmedterm_en.html	Spanish dataset, UMLS normalisations
^36^		Entities	P, O	660 RCT abstracts	Yes, https://gitlab.com/tomaye/abstrct	Relation extraction, domains neoplasm, glaucoma, hepatitis, diabetes, hypertension
^112^	^50^	Entities	P, IC, O, Design	99 RCT abstracts	Yes, https://github.com/jetsunwhitton/RCT-ART	Excluded for containing only glaucoma studies
^34^	^67^	Entities	O	300 abstracts	Yes, https://github.com/LivNLP/ODP-tagger	Own data + adaptation of EBM-NLP with normalization to 38 domains and 5 outcome-areas
^33^		Entities	I	1807 abstracts, labelled automatically by matching intervention strings from clinical trial registration	Yes, https://data.mendeley.com/datasets/ccfnn3jb2x/1	
^60^		Sentences	P, IC, O	42000 sentences	Yes, https://github.com/smileslab/Brain_Aneurysm_Research/tree/master/BioMed_Summarizer	Own data on brain aneurysm + existing dataset from Jin and Szolovits ^96^
^74^		Sentences, Entities	P, IC, O	130 abstracts from MEDLINE's PubMed Online PICO interface	Yes, https://github.com/nstylia/pico_entities/	
^99^		Entities	I,C,O	10 RCT abstracts	Yes, https://www.ncbi.nlm.nih.gov/pmc/articles/PMC8135980/bin/ocab077_supplementary_data.pdf	Relation extraction, domain COVID-19
^38^		Sentences	P, IC, O, N + CONSORT items	50 Full text RCTs	Yes, https://github.com/kilicogluh/CONSORT-TM	
^82^		Entities, Sentences	I, C, O + animal entities	400 RCT abstracts in first corpus, 10k abstract in additional corpus from mined data	Yes, https://osf.io/2dqcg/	Domain animal RCTs
^51^		Entities	P, I, C, O	211 RCT abstracts and 20 full texts	Yes, https://zenodo.org/record/6365890	
^70^		Entities	N	200 RCT fulltexts from PMC, annotated N from baseline tables	Yes, https://zenodo.org/record/6647853#.ZCa9dXbMJPY	
^63^based on ^111^		Entities	I, C, O	First corpus 160 abstracts, second corpus 20	Yes, https://github.com/bepnye/evidence_extraction/blob/master/data/exhaustive_ico_fixed.csv	Second corpus is domain cancer
^39^		Sentences, Entities	P, IC, O	500 labelled abstracts for sentences and 100 for P, O entities	No	
^73^		Entities	O	1300 abstracts with 3100 outcome statements	No	Domain cancer
^63^^,^^111^						
^45^		Entities	P, IC, O	Cochrane-provided dataset with 10137 abstracts	No	
^61^	^113^	Sentences and entities	P, N, sections	3657 structured abstracts with sentence tags, 204 abstracts with N (total) entities	No	
^57^		Structured, auto-labelled RCT abstracts with sentence tags and 378 documents with entity-level IR query-retrieval tags	P, IC, O	15,000 abstracts + 378 documents with IR tags	No	
^84^	^83^ (unclear)	Sentences and entities	IC, O, N (total + per arm)	263 abstracts	No	
^76^	^53, 58^	100 abstracts with P, Condition, IC, possibly on entity level. For O, 633 abstracts are annotated on sentence level.	P, Condition, IC, 0	633 abstracts for O, 100 for other classes	No	
^77^		Entities	Age, Design, Setting (Country), IC, N, study dates and affiliated institutions	185 full texts (at least 93 labelled)	No	
^79^		Sentences and entities	P, IC, Age, Gender, Design, Condition, Race	2000 sentences from abstracts	No	
^93^		200 abstracts, 140 contain sentence and entity labels	P, IC	200 abstracts	No	
^114^		Auto-labelled structured abstracts, sentence level.	P, IC, O	14200+ abstracts	No	
^94^		Entities	P, age, gender, race	50 abstracts	No	
^115^		Sentences (and entities?)	P, IC, O	3000 abstracts	No	
^42^		Entities	N (total)	648 abstracts	No	
^90^		Entities	IC	330 abstracts	No	
^66^		Indonesian text with sentence annotations	P,I,C,O	200 abstracts	No	
^68^		Sentences from 69 (heart) +24 (random) RCTs included in Cochrane reviews	Inclusion criteria	69 + 24 full texts	No	Domain cardiology
^80^		Sentences and entities	P, IC, Age, Gender, P (Condition or disease)	200 abstracts	No	
^71^		4,824 sentences from 18 UpToDate documents and 714 sentences from MEDLINE citations for P. For I: CLEF 2013 shared task, and 852 MEDLINE citations	P, IC, P (Condition or disease)	abstracts, full texts	No	General topic and cardiology domain
^41^	^102^	Entity annotation as noun phrases	O, IC	100 + 132 sentences from full texts	No	Diabetes and endocrinology journals as source
^92^	^103^	Auto-labelled structured RCT abstract sentences. ^92^ has 19,854 sentences, assumed same corpus as authors and technique are the same.	P, IC, O	23,472 abstracts	No	
^46^		RCTs abstracts and full texts: 132 + 50 articles	IC (per arm), IC (drug entities.), O (time point), O (primary or secondary outcome), N (total), Eligibility criteria, Enrolment dates, Funding org, Grant number, Early stopping, Trial registration, Metadata	132 + 50 abstracts and full texts	No	
^86^		Sentences and entities	P, IC, O, N (per arm + total)	48 full texts	No	
^49^		Studies from 5 systematic reviews on environmental health exposure, entities	P, O, Country, Exposure	Studies from 5 systematic reviews	No	Observational studies on environmental health exposure in humans
^44^		Labelled via supervised distant supervision. Full texts (~12500 per class), 50 + 133 manually annotated for evaluation.	P, IC, O	12700+ full texts	No	
^89^		Sentence labels, structured & unstructured abstracts. Manually annotated: 344 IC, 341 O, and 144 P and more derived by automatic labelling.	P, IC, O	344+ abstracts	No	
^88^		Entities	P, IC, O, O as "Instruments" or "Study Variables"	20 full texts/abstracts	No	
^85^		Entities (Brat, IOB format)	P, IC, O	170 abstracts	No	
^59^		Entities assigned to UMLS concepts (probably Cochrane corpus, size unclear). '88 instances, annotated in total with 76, 87, and 139 [P, IC, O respectively]'	P, IC, O	Unclear, at least 88 documents	No	
^43^		Sentences and entities	P, IC (per arm), N (total)	1750 title or abstracts	No	
^116^		Excluded paper, no data extraction system. Corpus of Patient, Population, Problem, Exposure, Intervention, Comparison, Outcome, Duration and Results sentences in abstracts.			No	Excluded from review, but describes relevant corpus
^56^		Sentences and entities	P, IC (per arm), O, multiple more	88 full texts	No	

3.4.4.3 Is the use of third-party frameworks reported and are they accessible?

Of the included publications in the base-review, 47 out of 53 (88%) described using at least one third-party framework for their data extraction systems. The following list is likely to be incomplete, due to non-available code and incomplete reporting in the included publications. Most commonly, there was a description of machine-learning toolkits (Mallet, N = 12; Weka, N = 6; tensorflow, N = 5; scikit-learn, N = 3). Natural language processing toolkits such as Stanford parser/CoreNLP (N = 12) or NLTK (N = 3), were also commonly reported for the pre-processing and dependency parsing steps within publications. The MetaMap tool was used in nine publications, and the GENIA tagger in four. For the complete list of frameworks please see Appendix A and D in *Underlying data.* ^127^

***3.4.5 Internal and external validity of the model***

3.4.5.1 Does the dataset or assessment measure provide a possibility to compare to other tools in the same domain?

With this item we aimed to assess publications to see if the evaluation results from models are comparable with the results from other models. Ideally, a publication would have reported the results of another classification model on the same dataset, either by re-implementing the model themselves ^96^ or by describing results of other models when using benchmark datasets. ^64^ This was rarely the case for the publications in the base-review, as most datasets were curated and used in single publications only. However, the re-use of benchmark corpora increased with the publications in the LSR update, where we found 40 publications that report results on one of the previously published benchmark datasets (see Table 4).

Addtionally, in the base-review, in 40 publications (75%) data were well described, and they utilised commonly used entities and common assessment metrics, such as precision, recall, and F1-scores, leading to a limited comparability of results. In these cases, the comparability is limited because those publications used different data sets, which can influence the difficulty of the data extraction task and lead to better results within for example structured datasets or topic-specific datasets.

3.4.5.2 Are explanations for the influence of both visible and hidden variables in the dataset given?

This item relates only to publications using machine learning or neural networks. Rule-based classification systems (N = 8, 15% reporting rule-base as sole approach) are not applicable to this item, because the rules leading to decisions are intentionally chosen by the creators of the system and are therefore always visible.

Ten publications in the base-review (19%) discussed hidden variables. ^83^ discussed that the identification of the treatment group entity yielded the best results. However, when neither the words ‘group’ nor ‘arm’ were present in the text then the system had problems with identifying the entity. ‘Trigger tokens’ ^104^ and the influence of common phrases were also described by Ref. 68, the latter showed that their system was able to yield some positive classifications in the absence of common phrases. ^103^ went a step further and provided a table with words that had the most impact on the prediction of each class. ^57^ describes removing sentence headings in structured abstracts in order to avoid creating a system biased towards common terms, while Ref. 90 discussed abbreviations and grammar as factors influencing the results. Length of input text ^59^ and position of a sentence within a paragraph or abstract, e.g. up to 10% lower classification scores for certain sentence combinations in unstructured abstracts, were shown in several publications. ^46^^,^^66^^,^^102^

3.4.5.3 Is the process of avoiding overfitting or underfitting described?

‘Overfitted’ is a term used to describe a system that shows particularly good evaluation results on a specific dataset because it has learned to classify noise and other intrinsic variations in the data as part of its model. ^105^

Of the included publications in the base-review, 33 out of 53 (62%) reported that they used methods to avoid overfitting. Eight (15%) of all publications reported rule-based classification as their only approach, allowing them to not be susceptible to overfitting by machine learning.

Furthermore, 28 publications reported cross-validation to avoid overfitting. Mostly these classifiers were in the domain of machine-learning, e.g. SVMs. Most commonly, 10 folds were used (N = 15), but depending on the size of evaluation corpora, 3, 6, 5 or 15 folds were also described. Two publications ^55^^,^^85^ cautioned that cross-validation with a high amount of folds (e.g. 10) causes high variance in evaluation results when using small datasets such as NICTA-PIBOSO. One publication ^104^ stratified folds by class in order to avoid this variance in evaluation results in a fold which is caused by a sparsity of positive instances.

Publications in the neural and deep-learning domain described approaches such as early stopping, dropout, L2-regularisation, or weight decay. ^59^^,^^96^^,^^106^ Some publications did not specifically discuss overfitting in the text, but their open-source code indicated that the latter techniques were used. ^55^^,^^75^

3.4.5.4 Is the process of splitting training from validation data described?

Random allocation to treatment groups is an important item when assessing bias in RCTs, because selective allocation can lead to baseline differences. ^1^ Similarly the process of splitting a dataset randomly, or in a stratified manner, into training (or rule-crafting) and test data is important when constructing classifiers and intelligent systems. ^117^

All included publications in the base-review gave indications of how different train and evaluation datasets were obtained. Most commonly there was one dataset and the splitting ratio which indicated that splits were random. This information was provided in 36 publications (68%).

For publications mentioning cross-validation (N = 28, 53%) we assumed that splits were random. The ratio of splitting (e.g. 80:20 for training and test data) was clear in the cross-validation cases and was described in the remainder of publications.

It was also common for publications to use completely different datasets, or multiple iterations of splitting, training and testing (N = 13, 24%). For example Ref. 56 used cross-validation to train and evaluate their model, and then used an additional corpus after the cross-validation process. Similarly Ref. 59, used 60:40 train/test splits, but then created an additional corpus of 88 documents to further validate the model’s performance on previously unseen data.

3.4.5.5 Is the model’s adaptability to different formats and/or environments beyond training and testing data described?

For this item we aimed to find out how many of the included publications in the base-review tested their data extraction algorithms on different datasets. A limitation often noted in the literature was that gold-standard annotators have varying styles and preferences, and that datasets were small and limited to a specific literature search. Evaluating a model on multiple independent datasets provides the possibility of quantifying how well data can be extracted across domains and how flexible a model is in real-life application with completely new data sets. Of the included publications, 19 (36%) discussed how their model performed on datasets with characteristics that were different to those used for training and testing. In some instances, however, this evaluation was qualitative where the models were applied to large unlabelled, real-life datasets. ^46^^,^^58^^,^^69^^,^^48^^,^^95^^,^^101^^,^^102^

***3.4.6 Other***

3.4.6.1 Caveats

Caveats were extracted as free text. Included publications (N = 64, 86%) reported a variety of caveats. After extraction we structured them into six different domains:

1. Label-quality and inter-annotator disagreements
2. Variations in text
3. Domain adaptation and comparability
4. Computational or system architecture implications
5. Missing information in text or knowledge base
6. Practical implications

These are further discussed in the ‘Discussion’ section of this living review.

3.4.6.2 Sources of funding and conflict of interest

**Figure 11.**
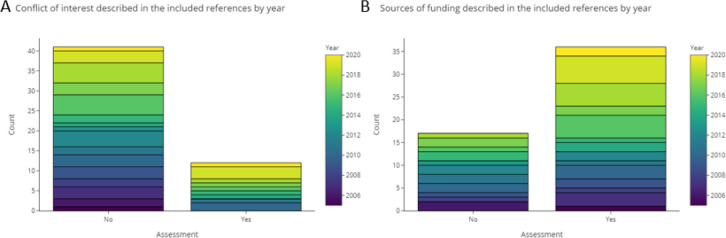
Declaration of funding sources and conflict of interest in the included studies.

Figure 11 shows that most of the included publications in the base review did not declare any conflict of interest. This is true for most publications published before 2010, and about 50% of the literature published in more recent years. However, sources of funding were declared more commonly, with 69% of all publications including statements for this item. This reflects a trend of more complete reporting in more recent years.

## 4 Discussion

### 4.1 Summary of key findings

***4.1.1 System architectures***

Systems described within the included publications are changing over time. Non-machine-learning data extraction via rule-base and API is one of the earliest and most frequently used approaches. Various classical machine-learning classifiers such as naïve Bayes and SVMs are very common in the literature published between 2005-2018. Up until 2020 there was a trend towards word embeddings and neural networks such as LSTMs. Between 2020 and 2022 we observed a trend towards transformers, especially the BERT, RoBERTa and ELECTRA architectures pre-trained on biomedical or scientific text.

***4.1.2 Evaluation***

We found that precision, recall, and F1 were used as evaluation metrics in most publications, although sometimes these metrics were adapted or relaxed in order to account for partial or similar matches.

***4.1.3 Scope***

Most of the included publications focused on extracting data from abstracts. The reasons for this include the availability of data and ease of access, as well as the high coverage of information and the availability of structured abstracts that can automatically derive labelled training data. A much smaller number of the included publications (n=19, 25%) extracted data from full texts. Half of the systems that extract data from full text were published within the last seven years. In systematic review practice, manually extracting data from abstracts is quicker and easier than manually extracting data from full texts. Therefore, the potential time-saving and utility of full text data extraction is much higher because more time can be saved by automation and it provides automation that more closely reflects the work done by systematic reviewers in practice. However, the data extraction literature on full text is still sparse and extraction from abstracts may be of limited value to reviewers in practice because it carries the risk of missing information. Whenever a publication reported full-text extraction we tried to find out if this also included abstract text, in which case we would count the publication in both categories. However, this information was not always clearly reported.

***4.1.4 Target texts***

Reports of randomised controlled trials were the most common texts used for data extraction. Evidence concerning data extraction from other study types was rare and is discussed further in the following sections.

### 4.2 Assessment of the quality of reporting

We only assessed full quality of reporting in the base-review, and assessed selected items during the review update. The quality of reporting in the included studies in the base-review is improving over time We assessed the included publications based on a list of 17 items in the domains of reproducibility, transparency, description of testing, data availability, and internal and external validity.

Base-review: Reproducibility was high throughout, with information about sources of training and evaluation data reported in 94% of all publications and pre-processing described in 89%.

Base-review: In terms of transparency, 81% of the publications provided a clear description of their algorithm, 94% described the characteristics of their datasets, but only 9% mentioned hardware specifications or feasibility of using their algorithm on large real-world datasets such as PubMed.

Update: Availability of source code was high in the publications added in the LSR update (N=19, 83%). Before the update, 15% of all included publications had made their code available. Overall, 39% (N=30) now have their code available and all links to code repositories are shown in Table 2.

Base-review: Testing of the systems was generally described, 89% gave a detailed assessment of their algorithms. Trade-offs between precision and recall were discussed in 32%.

Update: Basic metrics were reported in only 19% (N=14) of the included publications, which is a downward trend from 24% in the base-review. However, more complete reporting of source-code and public datasets still leads to increased transparency and comparability.

Update: Availability of the final models as end-user tools was very poor. Only 12% of the included publications had an application associated with it, but only 5 (6%) are available and directly usable via web-apps (see Table 3 for links). Furthermore, it is unclear how many of the other tools described in the literature are used in practice, even if only used internally within their authors research groups. There was a surprisingly strong trend towards sharing and re-using already published corpora in the LSR update. Earlier, labelled training and evaluation data were available from 13% of the publications, and only a further 32% of all publications reported using one of these available datasets. Within the LSR update, 22 corpora were available online and at least 40 other included publication mention using them. Table 4 provides the sources of all corpora and publications using them. For named-entity recognition, EBM-NLP ^55^ is the most popular dataset, used by at least 10 other publications and adapted and used by another four. For sentence classification the NICTA gold-standard ^52^ is used by eight others, and the automatically labelled corpus by Jin and Szolovits ^96^ is used by five others and was adapted once. For relation extraction the EvidenceInference 2.0 corpus is gaining attention, being used in at least three other publications.

Base-review: A total of 88% of the publications described using at least one accessible third-party framework for their data extraction system. Internal and external validity of each model was assessed based on its comparability to other tools (75%), assessment of visible and hidden variables in the data (19%), avoiding overfitting (62%, not applicable to non-machine learning systems), descriptions of splitting training from validation data (100%), and adaptability and external testing on datasets with different characteristics (36%). These items, together with caveats and limitations noted in the included publications are discussed in the following section.

### 4.3 Caveats and challenges for systematic review (semi)automation

In the following section we discuss caveats and challenges highlighted by the authors of the included publications. We found a variety of topics discussed in these publications and summarised them under seven different domains. Due to the increasing trend of relation-extraction and text summarisation models we now summarise any challenges or caveats related to these within the updated text at the end of each applicable domain.

***4.3.1 Label-quality and inter-annotator disagreements***

**Figure 12.**
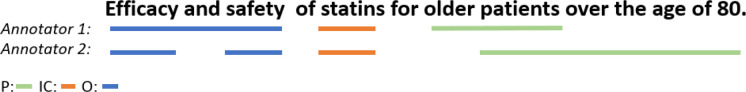
Example of inter-annotator disagreement. P, population; I, intervention; C, comparison; O, outcome.

The quality of labels in annotated datasets was identified as a problem by several authors. The length of the entity being annotated, for example O or P entities, often caused disagreements between annotators. ^46^^,^^48^^,^^58^^,^^69^^,^^95^^,^^101^^,^^102^ We created an example in Figure 12, which shows two potentially correct, but nevertheless different annotations on the same sentence.

Similar disagreements, ^65^^,^^85^^,^^104^ along with missed annotations, ^72^ are time-intensive to reconciliate ^97^ and make the scores less reliable. ^95^ As examples of this, two publications observed that their system performed worse on classes with high disagreement. ^75^^,^^104^ There exist different explanations for worse performance in these cases. It is possibly harder for models to learn from labelled data with systematic differences within. Another reason is that the model learns predictions based on one annotation style and therefore artificial errors are produced when evaluated against differently labelled data, or that the annotation task itself is naturally harder in cases with high inter-annotator disagreement, and therefore lower performance from the models might be explainable. An overview of the included publications discussing this, together with their inter-annotator disagreement scores, is given in Table 5.

**Table 5.** Examples for reports of inter-annotator disagreements in the included publications. Please see each included publication for further details on corpus quality.

Publication	Type	Score, or range between worst to best class
^43^	Average accuracy between annotators	Range: 0.62 to 0.70
^48^	Agreement rate	80%
^65^	Cohen’s Kappa	0.84 overall, down to 0.59 for worst class
^104^	Cohen’s Kappa	Range: 0.41 to 0.71
^75^	Inter-annotation recall	Range: 0.38 to 0.86
^55^	Cohen’s Kappa between experts	Range: 0.5 to 0.59
^55^	Macro-averaged worker vs. aggregation precision, recall, F1 (see publication for full scores)	Range: 0.39 to 0.70
^116^ (describes only PECODR corpus creation, excluded from review)	Initial agreement between annotators	Range: 85-87%
^52^	Average and range of agreement	62%, Range: 41-71
^58^	Avg. sentences labelled by expert vs. student per abstract	1.9 vs. 4.2
^58^	Cohen’s Kappa expert vs. student	0.42
^61^	Agreement; Cohen’s Kappa	86%; 0.76
^38^	MASI measure (Measuring Agreement on Set-Valued Items) for article/selection level; Krippendorff’s alpha for class-level	MASI 0.6 range 0.5-0.89; Krippendorf 0.53 for I, 0.57 for O, ranging from 0.06-0.96 between all classes
^35^	F1 strict vs. relaxed, at beginning and end of annotation phase	85.6% vs. 93.9% at the end; relaxed score increasing from 86% at beginning of annotation phase to 93.9% at the end
^36^	Fleiss’ Kappa on 47 abstracts for outcomes and on 30 for relation-extraction	Outcomes 0.81; Relations 0.62-0.72
^63^	B3, MUC, Constrained Entity-Alignment F-Measure (CEAFe) scores	B3 0.40; MUC 0.46; and CEAFe 0.42
^51^	Kappa for entities and F1 for complex entities with sub-classes or relations	Kappa range 0.74-0.68; complex entities 0.81
^37^	Cohen’s Kappa of their EBM-NLP adaptation vs. original dataset	Between 0.53 for P-0.69 for O

To mitigate these problems, careful training and guides for expert annotators are needed. ^58^^,^^77^ For example, information should be provided on whether multiple basic entities or one longer entity annotation are preferred. ^85^ Crowd-sourced annotations can contain noisy or incorrect information and have low interrater reliability. However, they can be aggregated to improve quality. ^55^ In recent publications, partial entity matches (i.e., token-wise evaluation) downstream were generally favoured above complete detection, which helps to mitigate this problem’s impact on final evaluation scores. ^55^^,^^83^

For automatically labelled or distantly supervised data, label quality is generally lower. This is primarily caused by incomplete annotation due to missing headings, or by ambiguity in sentence data, which is discussed as part of the next domain. ^44^^,^^57^^,^^103^

***4.3.2 Ambiguity***

The most common source of ambiguity in labels described in the included publications is associated with automatically labelled sentence-level data. Examples of this are sentences that could belong to multiple categories, e.g., those that should have both ‘P’ and an ‘I’ label, or sentences that were assigned to the class ‘other’ while containing PICO information (Refs. 54, 95, 96, among others). Ambiguity was also discussed with respect to intervention terms ^76^ or when distinguishing between ‘control’ and ‘intervention’ arms. ^46^ When using, or mapping to UMLS concepts, ambiguity was discussed in Refs. 41, 52, 72.

At the text level, ambiguity around the meaning of specific wordings was discussed as a challenge, e.g., the word 'concentration' can be a quantitative measure or a mental concept. ^41^ Numbers were also described as challenging due to ambiguity, because they can refer to the total number of participants, number per arm of a trial, or can just refer to an outcome-related number. ^84^^,^^113^ When classifying participants, the P entity or sentence is often overloaded because it includes too much information on different, smaller, entities within it, such as age, gender, or diagnosis. ^89^

Ambiguity in relation-extraction can include cases where interventions and comparators are classified separately in a trial with more than two arms, thus leading to an increased complexity in correctly grouping and extracting data for each separate comparison.

***4.3.3 Variations in text***

Variations in natural language, wording, or grammar were identified as challenges in many references that looked closer at the texts within their corpora. Such variation may arise when describing entities or sentences (e.g., Refs. 48, 79, 97) or may reflect idiosyncrasies specific to one data source, e.g., the position of entities in a specific journal. ^46^ In particular, different styles or expressions were noted as caveats in rule-based systems. ^42^^,^^48^^,^^80^

There is considerable variation in how an entity is reported, for example between intervention types (drugs, therapies, routes of application) ^56^ or in outcome measures. ^46^ In particular, variations in style between structured and unstructured abstracts ^65^^,^^78^ and the description lengths and detail ^59^^,^^79^ can cause inconsistent results in the data extraction, for example by not detecting information correctly or extracting unexpected information. Complex sentence structure was mentioned as a caveat especially for rule-based systems. ^80^ An example of a complex structure is when more than one entity is described (e.g., Refs. 93, 102) or when entities such as ‘I’ and ‘O’ are mentioned close to each other. ^57^ Finally, different names for the same entity within an abstract are a potential source of problems. ^84^ When using non-English texts, such as Spanish articles, it was noted that mandatory translation of titles can lead to spelling mistakes and translation errors. ^35^

Another common variation in text was implied information. For example, rather than stating dosage specifically, a trial text might report dosages of ‘10 or 20 mg’, where the ‘mg’ unit is implied for the number 10, making it a ‘dosage’ entity. ^46^^,^^48^^,^^90^

Implied information was also mentioned as problem in the field of relation-extraction, with Nye et al. (2021) ^63^ discussing importance of correctly matching and resolving intervention arm names that only imply which intervention was used. Examples are using ‘Group 1’ instead of referring to the actual intervention name, or implying effects across a group of outcomes, such as all adverse events. ^63^

***4.3.4 Domain adaptation and comparability***

Because of the wide variation across medical domains, there is no guarantee that a data extraction system developed on one dataset automatically adapts to produce reliable results across different datasets relating to other domains. The hyperparameter configuration or rule-base used to conceive a system may not retrieve comparable results in a different medical domain. ^40^^,^^68^ Therefore, scores might not be similar between different datasets, especially for rule-based classifiers, ^80^ when datasets are small, ^35^^,^^49^ when structure and distribution of class of interest varies, ^40^ or when the annotation guidelines vary. ^85^ A model for outcome detection, for example, might learn to be biased towards outcomes frequently appearing in a certain domain, such as chemotherapy-related outcomes for cancer literature or it might favour to detect outcomes more frequent in older trial texts if the underlying training data are older or outdated. ^73^ Another caveat mentioned by Refs. 59, 85 is that the size of the label space must be considered when comparing scores, as models that normalise to specific concepts rather than detecting entities tend to have lower precision, recall, and F1 scores.

Comparability between models might be further decreased by comparing results between publications that use relaxed vs. strict evaluation approaches for token-based evaluation, ^34^ or publications that use the same dataset but with different random seeds to split training and testing data. ^33^^,^^118^

Therefore, several publications discuss that a larger amount of benchmarking datasets with standardised splits for train, development, and evaluation datasets and standardised evaluation scripts could increase the comparability between published systems. ^46^^,^^92^^,^^114^

***4.3.5 Computational or system architecture implications***

Computational cost and scalability were described in two publications. ^53^^,^^114^ Problems within the system, e.g., encoding ^97^ or PDF extraction errors ^75^ lead to problems downstream and ultimately result in bias, favouring articles from big publishers with better formatted data. ^75^ Similarly, grammar and parsing part-of-speech and/or chunking errors (Refs. 76, 80, 90, among others) or faulty parse-trees ^78^ can reduce a system’s performance if it relies on access to correct grammatical structure. In terms of system evaluation, 10-fold cross-validation causes high variance in results when using small datasets such as NICTA-PIBOSO, ^54^^,^^85^^,^^104^ described that the same problem needs to be addressed through stratification of the positive instances of each class within folds.

***4.3.6 Missing information in text or knowledge base***

Information in text can be incomplete. ^114^ For example, the number of patients in a study might not be explicitly reported, ^76^ or abstracts lacking information about study design and methods can appear, especially in unstructured abstracts and older trial texts. ^91^^,^^96^ In some cases, abstracts can be missing entirely. These problems can sometimes be solved by considering the use of full texts as input. ^71^^,^^87^

Where a model relies on features, e.g., MetaMap, then missing UMLS coverage causes errors. ^72^^,^^76^ This also applies to models like CNNs that assign specific concepts, where unseen entities are not defined in the output label space. ^59^

In terms of automatic summarisation and relation extraction it was also cautioned that relying on abstracts will lead to a low sensitivity of retrieved information, as not all information of interest may be reported in sufficient detail to allow comprehensive summaries or statements about relationships between interventions and outcomes to be made. ^60^^,^^63^

***4.3.7 Practical and other implications***

In contrast to the problem of missing information, too much information can also have practical implications. For instance, often there are multiple sentences with each label, of which one is ‘key’, e.g., the descriptions of inclusion and exclusion criteria often span multiple sentences, and for a data extraction system it can be challenging to work out which sentence is the key sentence. The same problem applies to methods that select and rank the top-n sentences for each data extraction target, where a system risks including too much, or not enough results depending on the amount of sentences that are kept. ^46^

Low recall is an important practical implication, ^53^ especially in entities that appear infrequently in the training data, and are therefore not well represented in the training process of the classification system. ^48^ In other words, an entity such as ‘Race’ might not be labelled very often is a training corpus, and systematically missed or wrongly classified when the data extraction system is used on new texts. Therefore, human involvement is needed, ^86^ and scores need to be improved. ^41^ It is challenging to find the best set of hyperparameters ^106^ and to adjust precision and recall trade-offs to maximise the utility of a system while being transparent about the number of data points that might be missed when increasing system precision to save work for a human reviewer. ^69^^,^^95^^,^^101^

For relation extraction or normalisation tasks, error-propagation was noted as a practical issue in joint models. ^63^^,^^67^ To extract relations, first a model to identify entities is needed, and then another model to classify relationships is applied in a pipeline. Neither human nor machine can instantly perform perfect data extraction or labelling, ^37^ and thus errors done in earlier classification steps can be carried forwards and accumulate.

For relation extraction and summarisation, the importance of qualitative real-world evaluation was discussed. This was due to missing clarity of how well summarisation metrics relate to the actual usefulness or completeness of a summary and because challenges such as contradictions or negations within and between trial texts need to be evaluated within the context of a review and not just a trial itself. ^61^^,^^63^

A separate practical caveat with relation-extraction models are longer dependencies, i.e. bigger gaps between salient pieces of information in text that lead to a conclusion. This leads to increased complexity of the task and thus to reduced performance. ^99^

In their statement on ethical concerns, DeYoung et al. (2021) ^61^ mention that these complex relation and summarisation models can produce correct-looking but factually incorrect statements and are risky to be applied in practice without extra caution.

### 4.4 Explainability and interpretability of data extraction systems

The neural networks or machine-learning models from publications included in this review learn to classify and extract data by adjusting numerical weights and by applying mathematical functions to these sets of weights. The decision-making process behind the classification of a sentence or an entity is therefore comparable with a black box, because it is very hard to comprehend how, or why the model made its predictions. A recent comment published in Nature has called for a more in-depth analysis and explanation of the decision-making process within neural networks. ^117^ Ultimately, hidden tendencies in the training data can influence the decision-making processes of a data extraction model in a non-transparent way. Many of the examples discussed in the comment are related to healthcare, but in practice there is a very limited understanding of their inherent biases despite the broad application of machine learning and neural networks. ^117^

A deeper understanding of what occurs between data entry and the point of prediction can benefit the general performance of a system, because it uncovers shortcomings in the training process. These shortcomings can be related to the composition of training data (e.g. overrepresentation or underrepresentation of groups), the general system architecture, or to other unintended tendencies in a system’s prediction. ^119^ A small number of included publications in the base-review (N = 10) discussed issues related to hidden variables as part of an extensive error analysis (see section 3.5.2). The composition of training and testing data were described in most publications, but no publication that specifically addresses the issues of interpretability or explainability was found.

### 4.5 Availability of corpora, and copyright issues

There are several corpora described in the literature, many with manual gold-standard labels (see Table 4). There are still publications with custom, unshared datasets. Possible reasons for this are concerns over copyright, or malfunctioning download links from websites mentioned in older publications. Ideally, data extraction algorithms should be evaluated on different datasets in order to detect over-fitting, to test how the systems react to data from different domains and different annotators, and to enable the comparison of systems in a reliable way. As a supplement to this manuscript, we have collected links to datasets in Table 4 and encourage researchers to share their automatically or manually annotated labels and texts so that other researchers may use them for development and evaluation of new data extraction systems.

### 4.6 Latest developments and upcoming research

This is a timely LSR update, since it has a cut-off just before a the arrival of a new generation of tools: generative ‘Large Language Models’ (LLMs), such as ChatGPT from OpenAI, based on the GPT-3.5 model [ 1]. ^120^ As such, it may mark the current state of the field at the end of a challenging period of investigation, where the limitations of recent machine learning approaches have been apparent, and the automation of data extraction was quite limited.

**Figure 13.**
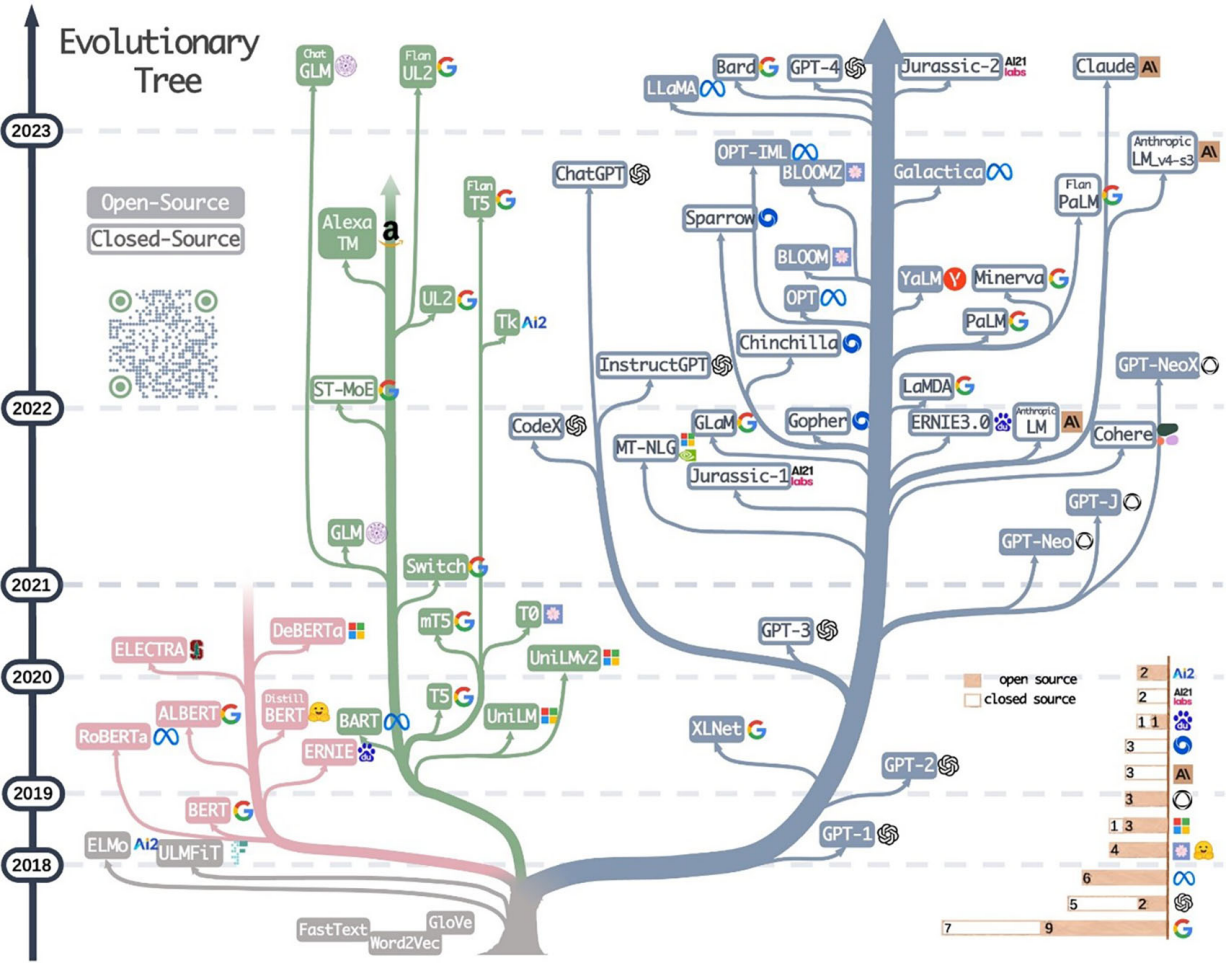
The evolutionary tree of language models, reproduced from Yang et al. ^122^ as published in their paper 'Harnessing the Power of LLMs in Practice: A Survey on ChatGPT and Beyond'.

The arrival of transformer-based methods in 2018 marked the last big change in the field, as documented by this LSR. Methods of our included papers only rarely progressed beyond the original BERT architecture, ^14^ varying mostly just in terms of datasets used in pre-training. Few used models only marginally different to BERT, such as RoBERTa with its altered pre-training strategy. ^121^ However, Figure 13 (reproduced from Yang et al. (2023) ^122^) shows that there has been a vast amount of NLP research and whole families of new methods that have not yet been tested to advance our target task of data extraction. For example within the new GPT-4 technical report, OpenAI describe increased performance, predictability, and closer adherence to the expected behaviour of their model, ^123^ and some other (open-source) LLMs shown in Figure 13 may have similar potential.

Early evaluations of LLMs suggest that these models may produce a step-change in both the accuracy and the efficiency of automated information extraction, while in parallel reducing the need for expensive labelled training data: a pre-print by Shaib et al. ^124^ describes a new dataset [ 2] and an evaluation of GPT-3-produced RCT summaries; ^124^ Wadhwa, DeYoung, et al. ^125^ use the Evidence Inference dataset and it’s annotations of RCT intervention-comparator-outcome triplets to train and evaluate BRAN, DyGIE++, ELI, BART, T5-base, and several FLAN models in a pre-print; ^125^ and in a separate pre-print Wadhwa, Amir, et al. ^126^ used the Flan-T5 and GPT-3 models to extract and predict relations between drugs and adverse events. ^126^ In the near future we expect the number of studies in this review to grow, as more evaluations of LLMs move into pre-print or published literature.

***4.6.1 Limitations of this living review***

This review focused on data extraction from reports of clinical trials and epidemiological research. This mostly includes data extraction from reports of randomised controlled trials where intervention and comparators are usually jointly extracted, and only a very small fraction of the evidence that addresses other important study types (e.g., diagnostic accuracy studies). During screening we excluded all publications related to clinical data (such as electronic health records) and publications extracting disease, population, or intervention data from genetic and biological research. There is a wealth of evidence and potential training and evaluation data in these publications, but it was not feasible to include them in the living review.

## 5. Conclusion

This LSR presents an overview of the data-extraction literature of interest to different types of systematic review. We included a broad evidence base of publications describing data extraction for interventional systematic reviews (focusing on P, IC, and O classes and RCT data), and a very small number of publications extracting epidemiological and diagnostic accuracy data. Within the LSR update we identified research trends such as the emergence of relation-extraction methods, the current dominance of transformer neural networks, or increased code and dataset availability between 2020-2022. However, the number of accessible tools that can help systematic reviewers with data extraction is still very low. Currently, only around one in ten publications is linked to a usable tool or describes an ongoing implementation.

The data extraction algorithms and the characteristics of the data they were trained and evaluated on were well reported. Around three in ten publications made their datasets available to the public, and more than half of all included publications reported training or evaluating on these datasets. Unfortunately, usage of different evaluation scripts, different methods for averaging of results, or custom adaptations to datasets still make it difficult to draw conclusions on which is the best performing system. Additionally, data extraction is a very hard task. It usually requires conflict resolution between expert systematic reviewers when done manually, and consequently creates problems when creating the gold standards used for training and evaluation of the algorithms in this review.

We listed many ongoing challenges in the field of data extraction for systematic review (semi) automation, including ambiguity in clinical trial texts, incomplete data, and previously unseen data. With this living review we aim to review the literature continuously as it becomes available. Therefore, the most current review version, along with the number of abstracts screened and included after the publication of this review iteration, is available on our website.

## Data availability

### Underlying data

Harvard Dataverse: Appendix for base review. https://doi.org/10.7910/DVN/LNGCOQ. ^127^

This project contains the following underlying data:

• Appendix_A.zip (full database with all data extraction and other fields for base review data)
• Appendix B.docx (further information about excluded publications)
• Appendix_C.zip (code, weights, data, scores of abstract classifiers for Web of Science content)
• Appendix_D.zip (full database with all data extraction and other fields for LSR update)
• Supplementary_key_items.docx (overview of items extracted for each included study)
• table 1. csv and table 1_long.csv (Table A1 in csv format, the long version includes extra data)
• table 1_long_updated.csv (LSR update for Table A1 in csv format, the long version includes extra data)
• included.ris and background.ris (literature references from base review)

Harvard Dataverse: Available datasets for SR automation. https://doi.org/10.7910/DVN/0XTV25. ^128^

This project contains the following underlying data:

• Datasets shared by authors of the included publications

Data are available under the terms of the Creative Commons Zero “No rights reserved” data waiver (CC0 1.0 Public domain dedication).

### Extended data

Open Science Framework: Data Extraction Methods for Systematic Review (semi)Automation: A Living Review Protocol. https://doi.org/10.17605/OSF.IO/ECB3T. ^15^

This project contains the following extended data:

• Review protocol
• Additional_Fields.docx (overview of data fields of interest for text mining in clinical trials)
• Search.docx (additional information about the searches, including full search strategies)
• PRISMA P checklist for ‘Data extraction methods for systematic review (semi)automation: A living review protocol.’

Data are available under the terms of the Creative Commons Attribution 4.0 International license (CC-BY 4.0).

### Reporting guidelines

Harvard Dataverse: PRISMA checklist for ‘Data extraction methods for systematic review (semi)automation: A living systematic review’ https://doi.org/10.7910/DVN/LNGCOQ. ^127^

Data are available under the terms of the Creative Commons Zero “No rights reserved” data waiver (CC0 1.0 Public domain dedication).

### Software availability

The development version of the software for automated searching is available from Github: https://github.com/mcguinlu/COVID_suicide_living.

Archived source code at time of publication: http://doi.org/10.5281/zenodo.3871366. ^17^

License: MIT

## Author contributions

LS: Conceptualization, Investigation, Methodology, Software, Visualization, Writing – Original Draft Preparation

ANFM: Data Curation, Investigation, Writing – Review & Editing

RE: Data Curation, Investigation, Writing – Review & Editing

BKO: Conceptualization, Investigation, Methodology, Software, Writing – Review & Editing

JT: Conceptualization, Investigation, Methodology, Writing – Review & Editing

JPTH: Conceptualization, Funding Acquisition, Investigation, Methodology, Writing – Review & Editing

## Footnotes

^1^

https://openai.com/blog/chatgpt (last accessed 22/05/2023).

^2^

https://github.com/cshaib/summarizing-medical-evidence (last accessed 22/05/2022).
