# Supplementary material for: Data extraction methods for systematic review (semi)automation: Update of a living systematic review
Source: F1000Res. 2025 Apr 8;10:401. Originally published 2021 May 19. [Version 3] doi: 10.12688/f1000research.51117.3 (PMC8361807; doi:10.12688/f1000research.51117.3)
Supplement: Peer review history

# Reviewer report: Reviewer response for version 1

**DOI:** 10.5256/f1000research.54235.r89347

**Published:** 2021-08-26

**Authors:** Carmen Amezcua-Prieto

**Affiliations:** 1 Department of Preventive Medicine and Public Health, University of Granada, Granada, Spain

Data extraction in a systematic review is a hard and time-consuming task. The (semi) automation of data extraction in systematic reviews is an advantage for researchers and ultimately for evidence-based clinical practice. This living systematic review examines published approaches for data extraction from reports of clinical studies published up to a cut-off date of 22 April 2020. The authors included more than 50 publications in this version of their review that addressed extraction of data from abstracts, while less (26%) used full texts. They identified more publications describing data extraction for interventional reviews.  Publications extracting epidemiological or diagnostic accuracy data were limited.

 Main important issues have been addressed in the systematic review:

- This living systematic review has been justified. The field of systematic review (semi) automation is evolving rapidly along with advances in language processing, machine learning, and deep learning.

- Searching and update schedules have been clearly defined, shown in Figure 1.
- There are sufficient details of the methods and analysis provided to allow replication.
- Conclusions are drawn adequately supported by the results presented in the review.

A minor consideration is suggested:

-  An incomplete sentence in Methods: ‘We included reports published from 2005 until the present day, similar to’.

Are the rationale for, and objectives of, the Systematic Review clearly stated?

Yes

Is the statistical analysis and its interpretation appropriate?

Not applicable

Have the search and update schedule been clearly defined and justified?

Yes

Is the living method justified?

Yes

Are sufficient details of the methods and analysis provided to allow replication by others?

Yes

Are the conclusions drawn adequately supported by the results presented in the review?

Yes

Reviewer Expertise:

NA

I confirm that I have read this submission and believe that I have an appropriate level of expertise to confirm that it is of an acceptable scientific standard.

# Reviewer report: Reviewer response for version 1

**DOI:** 10.5256/f1000research.54235.r89348

**Published:** 2021-08-12

**Authors:** Kathryn A. Kaiser

**Affiliations:** 1 Department of Health Behavior, School of Public Health, University of Alabama at Birmingham, Birmingham, AL, USA

The authors have undertaken and documented the steps taken to monitor an area of research methods that is important to many around the world by use of a “living systematic review”. The specific focus is on automated or semi-automated data extraction around the PICO structure often used in biomedicine, whether it be to summarize a body of literature narratively or using meta-analysis techniques. A significant irony about the body of papers included in this review is that there is a large amount of missingness related to the performance of such methods. Those who conduct systematic reviews know well the degree of missing information sought to summarize a group of studies.

 Readers who will be most interested in this ongoing work can maintain an eye on the authors’ progress in identifying activities in this space. It is not clear, however, how long the funding will support this effort or how long the authors will remain engaged in advancing this project. The data represented in this paper does not give readers confidence that the community is approaching acceptable methods that are superior to other, less automated methods (the latter of which are not well-discussed).

 Some aspects of the paper would benefit from additional detail (in no particular order of importance):

1. The end game for the tracking of this area of literature is not explicitly described in the abstract, nor is it discussed to a great extent at the end of the paper. Much of the results presented do not paint a bright future for this area of research as conditions presently are. While the aim is laid out well in section 1.2, the large amount of missing performance data (reported to be 87%) is unable to address the “Is it reliable?” question. One might suspect that if particularly stellar performance were demonstrated by a project, those data would be prominently advertised. Thus, the yet-to-be-done contacting of authors step would be enlightening if either performance data can be obtained, or if authors remain silent on that request. This follow-up task will be a major point of interest for many who will follow updates to this paper. It is likely that the particular research context (e.g. see Pham *et al*., 2021 ^1^) will have a large degree of influence on the performance metrics to be had if they can be determined.
2. The description of how the 17 “Key items of interest” were determined and if there is a plan to put these forth as methodological guidelines or a reporting checklist would be helpful. Either of these would help to advance the field further.
3. On Page 5, the exclusions listed have the use of pre-processing of text, yet the results discuss the many papers that appear to have used that in their methods. Perhaps this is a deviation from the original protocol after the review began (an understandable decision)?
4. In section 2.4 about searching Pubmed, can the authors clarify that the Pubmed 2.0 API or GUI will be used to access candidate literature?
5. Also relevant to section 2.4 on searching, since GITHUB is so popular, might this also be a fruitful place to routinely search?
6. Clarification of the ability to obtain cited software packages (whether for no cost or at some cost) would be helpful.
7. Figure 3 explanation of PICO is a typo – “PCIO”.
8. Table 5 is shown before Table 1. Please check and correct flow and references to table numbers (5,1,4,2,3 is the flow now).
9. One of the major limitations to be noted is the unfortunate issue of the lack of specific data in abstracts about interventions and comparators.

Are the rationale for, and objectives of, the Systematic Review clearly stated?

Yes

Is the statistical analysis and its interpretation appropriate?

Yes

Have the search and update schedule been clearly defined and justified?

Yes

Is the living method justified?

Yes

Are sufficient details of the methods and analysis provided to allow replication by others?

Yes

Are the conclusions drawn adequately supported by the results presented in the review?

Yes

Reviewer Expertise:

Systematic reviews in biomedicine topics, issues with time and effort required to complete reviews with generally available tools.

I confirm that I have read this submission and believe that I have an appropriate level of expertise to confirm that it is of an acceptable scientific standard.

## References

[1] : Text mining to support abstract screening for knowledge syntheses: a semi-automated workflow. *Systematic Reviews* .2021;10(1) : 10.1186/s13643-021-01700-x 10.1186/s13643-021-01700-x 34039433 PMC8152711

# Reviewer report: Reviewer response for version 1

**DOI:** 10.5256/f1000research.54235.r85692

**Published:** 2021-06-08

**Authors:** Emma McFarlane

**Affiliations:** 1 Centre for Guidelines, National Institute for Health and Care Excellence, London, UK

This is a living systematic review of published methods and tools aimed at automating or semi-automating the process of data extraction in the context of a systematic review. Automating data extraction is an area of interest among evidence-based medicine. 

 The methods are sufficiently described to be replicated, but further details of analysis to determine the items of interest would be helpful to link into the results. Additionally, the authors may want to consider commenting on the topic areas covered by the included studies and whether that has an impact on any of the metrics measured. 

 In the discussion section, it's interesting that fewer studies extracted data from the full text. Could the authors comment on the implications of this in terms of using tools in a live review as it's not common to manually only extract data from an abstract.

Are the rationale for, and objectives of, the Systematic Review clearly stated?

Yes

Is the statistical analysis and its interpretation appropriate?

Not applicable

Have the search and update schedule been clearly defined and justified?

Yes

Is the living method justified?

Yes

Are sufficient details of the methods and analysis provided to allow replication by others?

Partly

Are the conclusions drawn adequately supported by the results presented in the review?

Yes

Reviewer Expertise:

Evidence-based medicine, systematic reviews, automation techniques.

I confirm that I have read this submission and believe that I have an appropriate level of expertise to confirm that it is of an acceptable scientific standard.
